# Catalyst-free, microdroplet-mediated waste plastic conversion to diacids

**DOI:** 10.1038/s41586-026-10746-7

**Published:** 2026-07-15

**Authors:** Ruiliang Gao, Liwei Zhang, Richard J. Lewis, Hao Wang, Zhiyan Pan, Yage Zhang, Zekai Yu, Zhiqiang Liu, Xiaolin Guo, Xiangbowen Du, Wencong Liu, Minghang Li, Shipan Liang, Bing Lu, Ichiro Daigo, Shanjun Mao, Graham J. Hutchings, Yong Wang

**Affiliations:** 1 Advanced Materials and Catalysis Group, Zhejiang Key Laboratory of Low-Carbon Synthesis of Value-Added Chemicals, State Key Laboratory of Clean Energy Utilization, Institute of Catalysis, Department of Chemistry, Zhejiang University, Hangzhou, People’s Republic of China; 2 Max Planck Cardiff Centre on the Fundamentals of Heterogeneous Catalysis (FUNCAT), Cardiff Catalysis Institute, School of Chemistry, Cardiff University, Cardiff, UK; 3 Research Center for Advanced Science and Technology (RCAST), The University of Tokyo, Meguro, Japan; 4 Institute of Environmental-Chemical Engineering, College of Environment, Zhejiang University of Technology, Hangzhou, People’s Republic of China; 5 College of Materials and Chemistry, China Jiliang University, Hangzhou, People’s Republic of China

**Keywords:** Synthetic chemistry methodology, Pollution remediation

## Abstract

Plastic waste accumulation poses a global threat to both the environment and public health^1–3^. Although catalytic upcycling to value-added chemicals holds promise, its industrial adoption is hindered by additive-induced catalyst deactivation, feedstock heterogeneity, process inflexibility and limited economic viability^4^. Here we report a catalyst-free upcycling strategy that makes use of in situ generation of hydroxyl radicals at microdroplet interfaces^5–8^ to enable oxidative cleavage of diverse waste plastics—from polyolefins to rubbers—into carboxylic acids under mild conditions. By eliminating catalyst-dependent pathways, this approach circumvents key challenges of catalyst design and poisoning, while substantially lowering technical barriers and costs^9,10^. Our method achieves complete conversion of polyethylene (PE) with selectivity to short-chain diacids approaching 69% under relatively mild conditions and demonstrated broad applicability to mixed commercial plastics, with scalability demonstrated up to the 300-g scale. Radical intermediate analysis reveals the crucial role of H_2_O in mediating a unique oxidative degradation mechanism: sequential hydroxyl radical addition to alkyl radicals, distinct from classical liquid-phase aerobic oxidation of alkane^11^. This interfacial radical-mediated strategy enables sustainable polymer upcycling with minimal infrastructure. More broadly, this work provides a scalable blueprint for the first, to our knowledge, industrial implementation of microdroplet chemistry, with transformative implications for oxidation processes in organic acid synthesis and beyond.

## Abstract (web summary)

A catalyst-free upcycling strategy based on in situ generation of hydroxyl radicals at microdroplet interfaces is described for efficient and scalable processing of polymers with minimal infrastructure, using only water and oxygen under mild conditions.

## Main

Global plastic waste reached 353 million tonnes in 2019, with nearly half landfilled^12–14^. If present trends persist, as much as 12 billion tonnes could accumulate in landfills or leak into the environment by 2050 (ref. ^15^). With growing environmental^1–3^ and health concerns^16^, associated with the incomplete degradation of polyolefins^13,16^ (which account for >60% of plastic production), efficient approaches to upcycle polyolefin wastes are urgently needed^17,18^. To achieve this, catalytic strategies such as catalytic cracking^19^, hydrogenolysis^20–22^, tandem hydrogenolysis aromatization^23^, dehydro-metathesis^24^, low-temperature tandem aromatization^25^ and oxidation upcycling^26^ have been developed for the cleavage of polyolefin chains. However, accumulation of miscellaneous additives and contaminants inevitably leads to catalyst deactivation^27,28^, whereas coke formation, high energy consumption and complex downstream processing make these approaches ineffective for complex real-world waste, thereby limiting industrial-scale implementation. Non-catalytic thermal cracking is a mature route that produces light olefins (for example, ethylene, propylene and butenes) through catalyst-free, rapid, continuous processing. However, high-temperature radical chemistry limits reaction control and often leads to unstable product distributions, coke formation, reactor fouling and high energy consumption (Supplementary Table 1). Therefore, developing a catalyst-free, sustainable approach to transform plastic wastes to more valuable chemicals is desirable.

Microdroplets have recently emerged as unique chemical reactors owing to their distinctive interfacial properties^6,7,26,29–35^. Intense electric fields (approximately 10^9^ V m^−1^) at the microdroplet interface^34,36–39^ can induce electron abstraction from hydroxide ions (OH^−^), leading to the formation of hydroxyl radicals (•OH) and solvated electrons^40^. These highly reactive species enable catalyst-free oxidation of small organic molecules such as toluene^41^ and styrene^42^ typically requiring co-solvents, beyond water. Interfacial properties also drive spontaneous urea formation from CO_2_ and ammonia^7^. However, present applications remain confined to microscale (millimolar) processes^42^, hindering industrial translation.

**Fig. 1:**
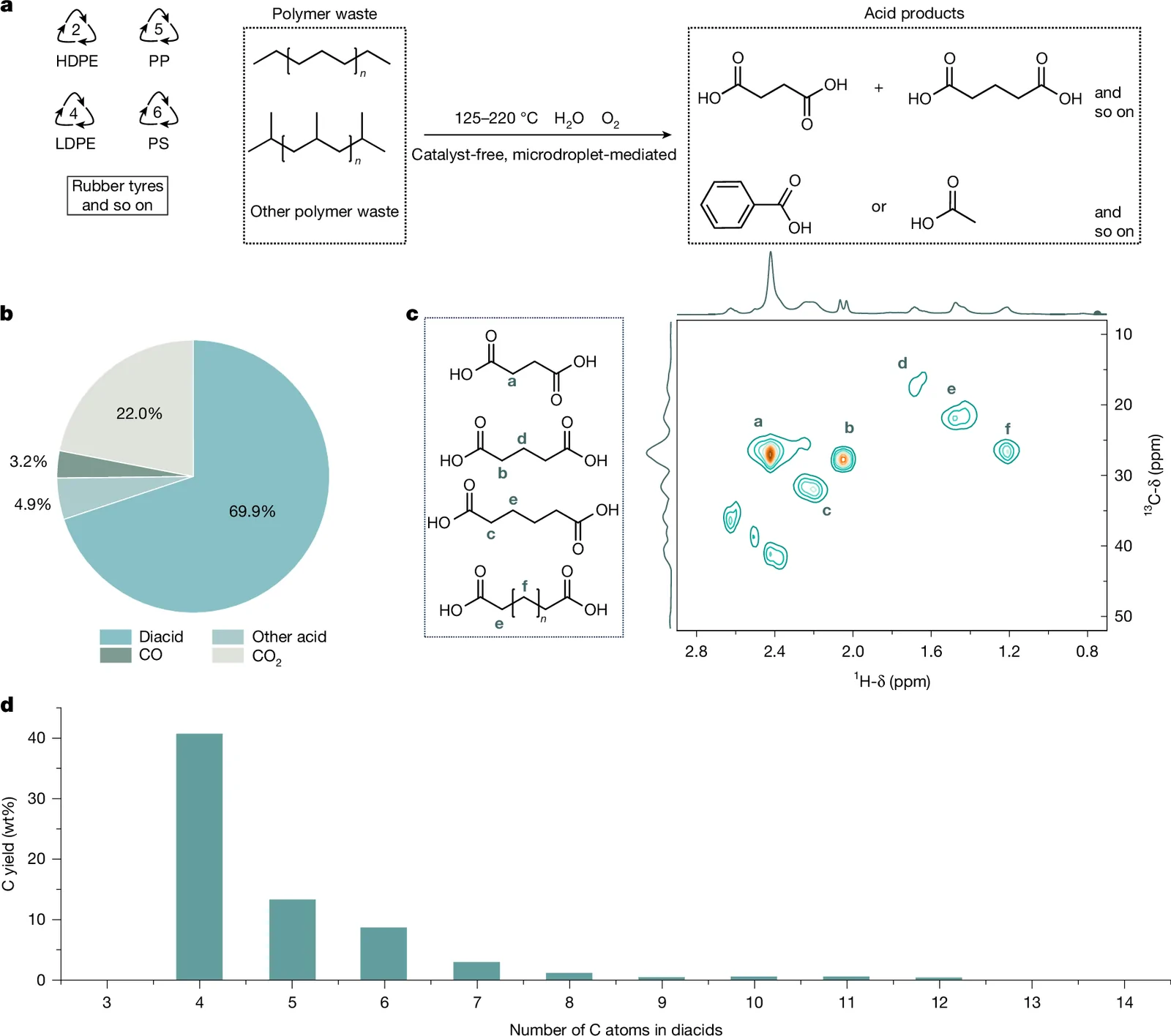
The oxidation degradation of PE in water under catalyst-free conditions. **a**, Reaction schemes of the proposed strategy for catalyst-free oxidation of plastic wastes. **b**, The degradation product composition of diacid produced under 125 °C and 2 MPa O_2_ at 18 h reaction time, in which ‘Other acid’ refers to monocarboxylic acid and unsaturated dicarboxylic acid identified by GC–MS (Extended Data Fig. 1b). Values are expressed as the mass fraction of each product relative to all isolated products (wt% C basis). **c**, HSQC of degradation product. **d**, The distribution of diacid produced under 125 °C and 2 MPa O_2_ at 18 h reaction time.

**Extended Data Fig. 1:**
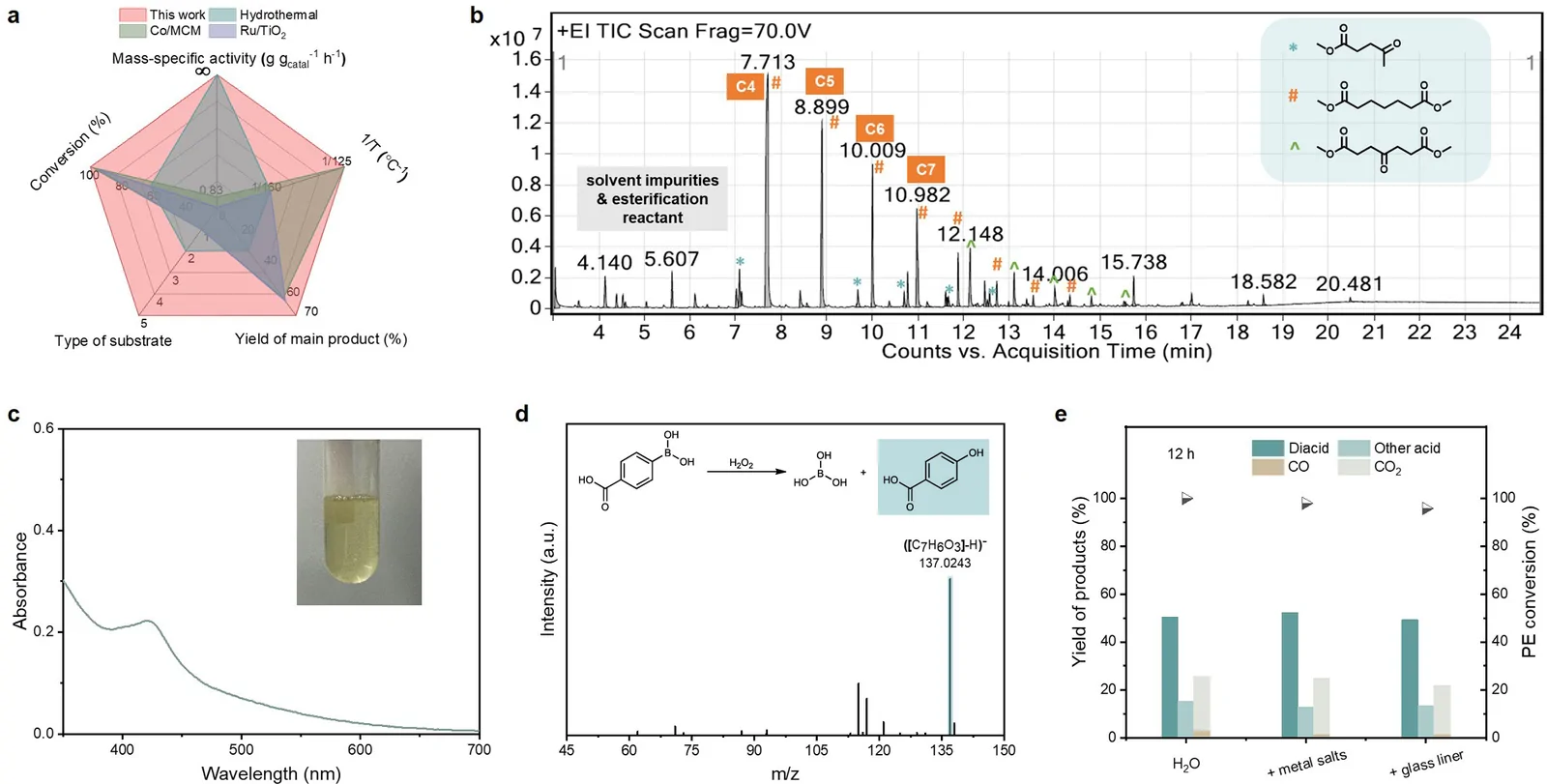
Performance comparison and hydroxyl radical validation of catalyst-free PE oxidation. **a**, The comparison of previously reported oxidation degradation routes for PE. Compared with the routes of catalytic and catalyst-free hydrothermal oxidation of PE, this work has notable comprehensive advantages in reaction conditions, application width and product yields. **b**, GC-MS analysis of the products (after esterification) obtained from PE oxidative degradation (under 125 °C and 2 MPa O_2_ at 18 h reaction time). Apart from solvent peaks and esterification reagents, the main peaks corresponded to saturated dicarboxylic acid, unsaturated dicarboxylic acid with keto on the carbon chain and monocarboxylic acid. **c**, The UV absorption spectrum of TMB oxidation-induced colour change caused by hydroxyl radicals. 0.5 ml 2 mM TMB/DMSO solution was added to 5 ml of pure water. After 12 h under 2 MPa O_2_ at 125 °C, the solution was detected by UV–Vis spectroscopy. **d**, Mass spectra of the pure water under 2 MPa O_2_ containing 4-CPB (illustrated as the reaction scheme of 4-CPB and H_2_O_2_ generated spontaneously to generate 4-HB and boric acid). **e**, The PE conversion and product distribution of deliberate addition of trace metal impurities (2.4 ppb Ni(NO_3_)_2_, 0.8 ppb Mo(NO_3_)_3_ and 0.77 ppb Fe(NO_3_)_3_) and elimination by adding a glass liner between pure water and the autoclave (2 MPa O_2_ at 125 °C after 12 h). No marked changes were observed, indicating negligible impact and ruling out homogeneous metal catalysis.

Inspired by these considerations, we introduce a catalyst-free oxidative strategy that converts diverse polymer wastes—including polyolefins, rubbers and mixed plastics—into value-added organic acids using only water and O_2_ under mild conditions (Fig. 1a). The hydrophobic, long-chain nature of molten polymers is used to generate dynamic water–oil interfacial microdroplets on stirring, in which hydroxyl radicals form spontaneously during oxidation. Continuous in situ •OH generation enables efficient C–H and C–C bond scission across chemically diverse polymers, while tolerating commercial additives and heterogeneous waste streams, offering a scalable and low-cost route for plastic upcycling. Using chemically inert PE, the highest-volume plastic, as a model substrate, our system achieves competitive degradation efficiency^26,43,44^ (Extended Data Fig. 1a), converting PE predominantly to C_4_–C_8_ diacids (125 °C, 2 MPa oxygen). The strategy was extended to diverse polyolefins and, in the case of post-consumer rubber tyres, scaled to 300-g batches.

In a proof-of-concept experiment, a low-molecular-weight PE sample (0.20 g, *M*_w_ = 4.6 × 10^3^ g mol^−^^1^) was mixed with 5 ml water in a 25-ml stirred autoclave. The reaction was carried out at 125 °C under 2 MPa oxygen. Under catalyst-free conditions, saturated dicarboxylic acids were synthesized with a 69.6% carbon yield (Fig. 1b), alongside 4.9% partially oxidized intermediates comprising monocarboxylic and unsaturated dicarboxylic acids. Structural characterization using nuclear magnetic resonance (NMR) and 2D ^13^C–^1^H heteronuclear single quantum coherence (HSQC) spectroscopy^45–47^ (Fig. 1c) revealed that the product spectrum was dominated by signals corresponding to short-chain diacids (peaks a, b and c), with only minor signals from longer alkyl chains (peak f) and other by-products. Gas chromatography (GC) and gas chromatography–mass spectrometry (GC–MS) analysis of esterified products revealed succinic acid as the dominant diacid species (58.4% of total diacids; Fig. 1d). The carbon number distribution of the other acids closely paralleled that of the saturated diacids, showing a declining yield with increasing carbon chain length (Extended Data Fig. 1b).

**Fig. 2:**
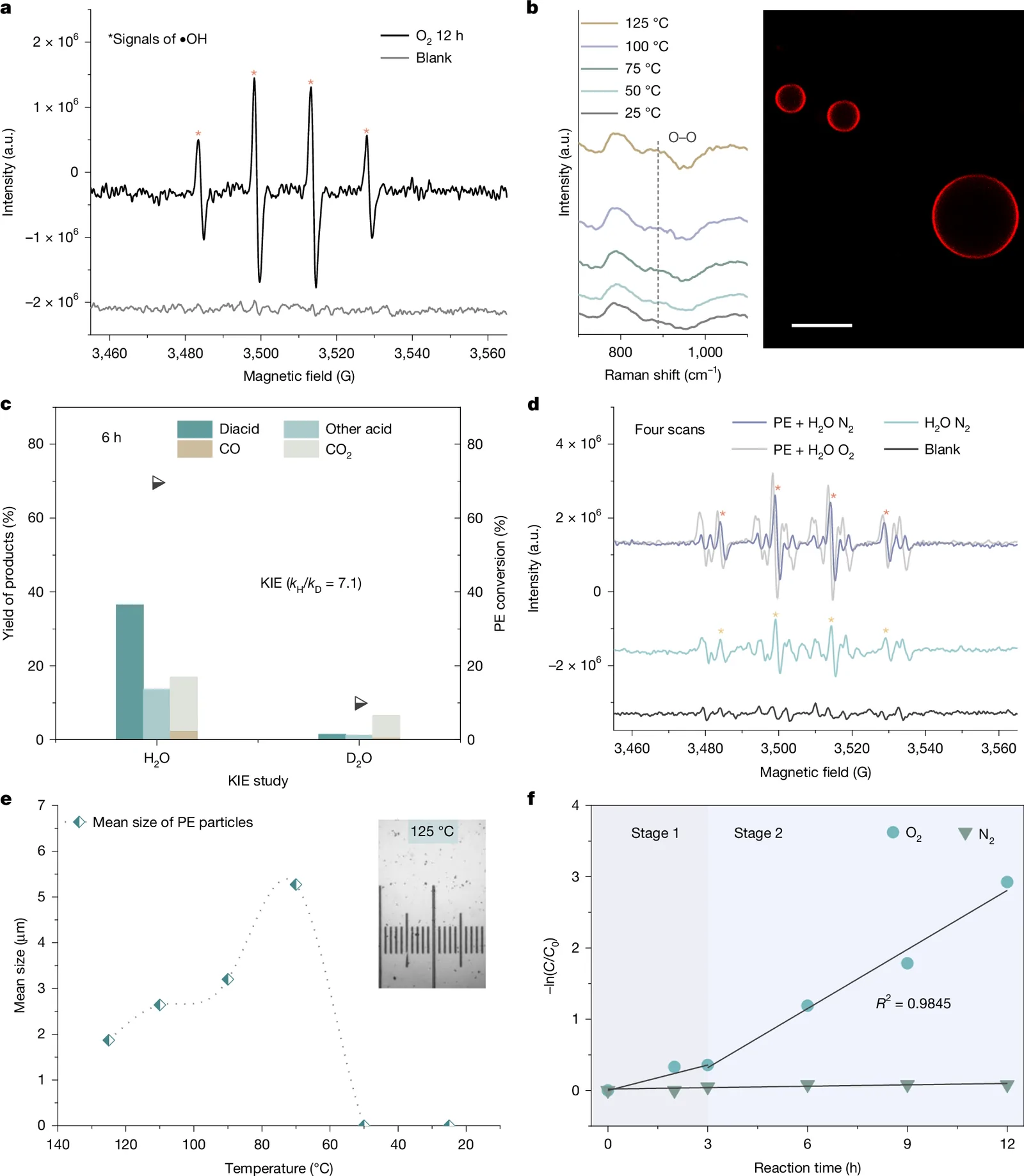
The generation and detection of hydroxyl radicals. **a**, EPR spectrum under 125 °C and 2 MPa O_2_ at 12 h reaction time. **b**, The O–O vibrational bands evolution for water confined in a sealed capillary from 25 to 125 °C and confocal fluorescence imaging on heptadecane microdroplets in Rhodamine 800-doped water. Scale bar, 5 μm. **c**, The yield of products and PE conversion using H_2_O and D_2_O at 6 h. KIE, kinetic isotope effect. **d**, EPR spectra of the system under 2 MPa N_2_ and 2 MPa O_2_, recorded with and without PE. **e**, Mean size of PE particles by means of intermediate sampling under different temperatures. **f**, Kinetic curve fitting under O_2_ and N_2_. a.u., arbitrary units.

**Extended Data Fig. 2:**
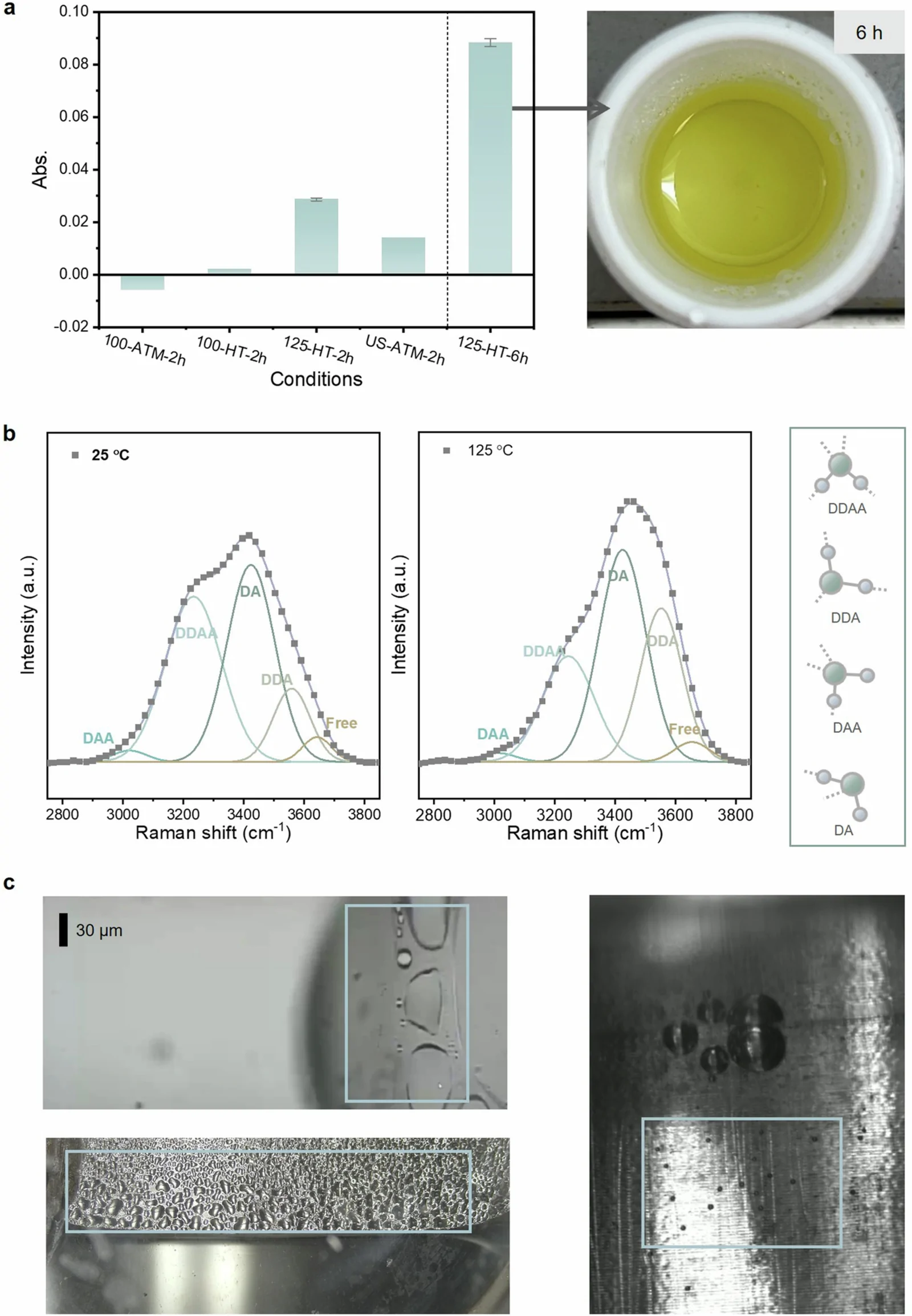
Identification of the microdroplet interface for spontaneous hydroxyl radical generation. **a**, UV absorbance of the reaction solution under different conditions: boiling at 100 °C and ambient pressure for 2 h; hydrothermal treatment at 100 °C in a PTFE autoclave for 2 h; hydrothermal treatment at 125 °C in a PTFE autoclave for 2 h; ultrasonic irradiation in water at ambient pressure for 2 h; hydrothermal treatment at 125 °C in an autoclave for 6 h. The colour of the water solution containing 2 mM TMB/DMSO under 125 °C in a hydrothermal reactor (6 h, photograph, right). To further validate the conditions required for hydroxyl radical (•OH) generation, we performed TMB colorimetric assays under three conditions. •OH formation was only observed under sealed conditions and its generation increased with both temperature and reaction time. Furthermore, when high-frequency ultrasound was applied under the beaker to drive rapid transitions among microdroplets, water vapour and bulk water phases, •OH was also detected under atmospheric pressure. This aligns with previous reports, suggesting that •OH originates from the microdroplet interface. **b**, The Raman OH stretching band for water confined in a sealed capillary at 25 and 125 °C, which can be deconvoluted into five sub-bands. **c**, The micron-sized condensed water droplets at the water surface in the sealed capillary at 125 °C and the condensed droplets and microbubbles grown on the reactor walls in the windowed reactor at 125 °C.

It is well known that the activation of C–H bonds involves a high energy barrier and the pyrolysis of PE typically requires temperatures exceeding 400 °C (ref. ^48^). Therefore, the degradation of PE under mild conditions (125 °C) is unlikely to proceed simply through thermal cracking. Control experiments in solvent-free conditions and various organic media (including methanol, acetonitrile and dichlorobenzene) confirmed the essential role of aqueous conditions, with the use of the organic solvents unable to offer conversion efficiency comparable with that observed in H_2_O (Supplementary Table 2). Instead, the reaction is most probably driven by the spontaneous generation of •OH, as evidenced by a strong electron paramagnetic resonance (EPR) signal observed when the freshly reacting mixture was directly injected into a 5,5-dimethyl-1-pyrroline *N*-oxide (DMPO) solution through the sample valve on the autoclave after 12 h of reaction (Fig. 2a). The presence of •OH was further confirmed by the colorimetric change of 3,3′,5,5′-tetramethylbenzidine (TMB) and the corresponding ultraviolet–visible absorption features (Extended Data Fig. 1c). MS also revealed •OH generation by means of conversion of 4-CPB to 4-hydroxybenzoic acid (*m*/*z* = 137.0243, deprotonated; Extended Data Fig. 1d). Inductively coupled plasma mass spectrometry analysis of post-reaction solutions revealed only negligible metal concentrations at the parts per billion (ppb) level (Supplementary Table 3). On the basis of this observation, and using a glass liner to prevent further contributions from the steel autoclave, we deliberately introduced metal species at concentrations identical to that observed during our inductively coupled plasma mass spectrometry analysis (2.4 ppb Ni(NO_3_)_2_, 0.8 ppb Mo(NO_3_)_3_ and 0.77 ppb Fe(NO_3_)_3_) into the reaction system (Extended Data Fig. 1e). However, no marked changes in PE conversion and product distribution were observed, ruling out contributions from homogeneous metals. Furthermore, to exclude reactor metal interference, we conducted the experiment in an all-polytetrafluoroethylene (PTFE) hydrothermal autoclave, with 20 ml of water and 2 ml of TMB in dimethyl sulfoxide (DMSO). After 6 h at 125 °C, vivid yellow colouration (Extended Data Fig. 2a) indicated •OH formation. In situ Raman spectroscopy of water confined in a sealed capillary showed temperature-dependent weakening of hydrogen bonding, shifting from ordered double-donor and double-acceptor (DDAA) to disordered double-donor and single-acceptor (DDA) and single-donor and single-acceptor (DA) modes^49^ (Extended Data Fig. 2b). Concurrent emergence of O–O vibrational bands confirmed in situ aqueous-phase H_2_O_2_ formation at elevated temperatures (Fig. 2b). We also observed dynamic behaviour of condensed micron-sized water droplets above the liquid level in the sealed capillary (Supplementary Video 1), alongside microbubbles and droplets forming on reactor walls (Extended Data Fig. 2c and Supplementary Video 2), all identified as potential •OH generation sites^6,33^. These results indicate metal-independent •OH formation, suggesting a route by means of surface-condensed droplets and microbubble interfaces.

**Extended Data Fig. 3:**
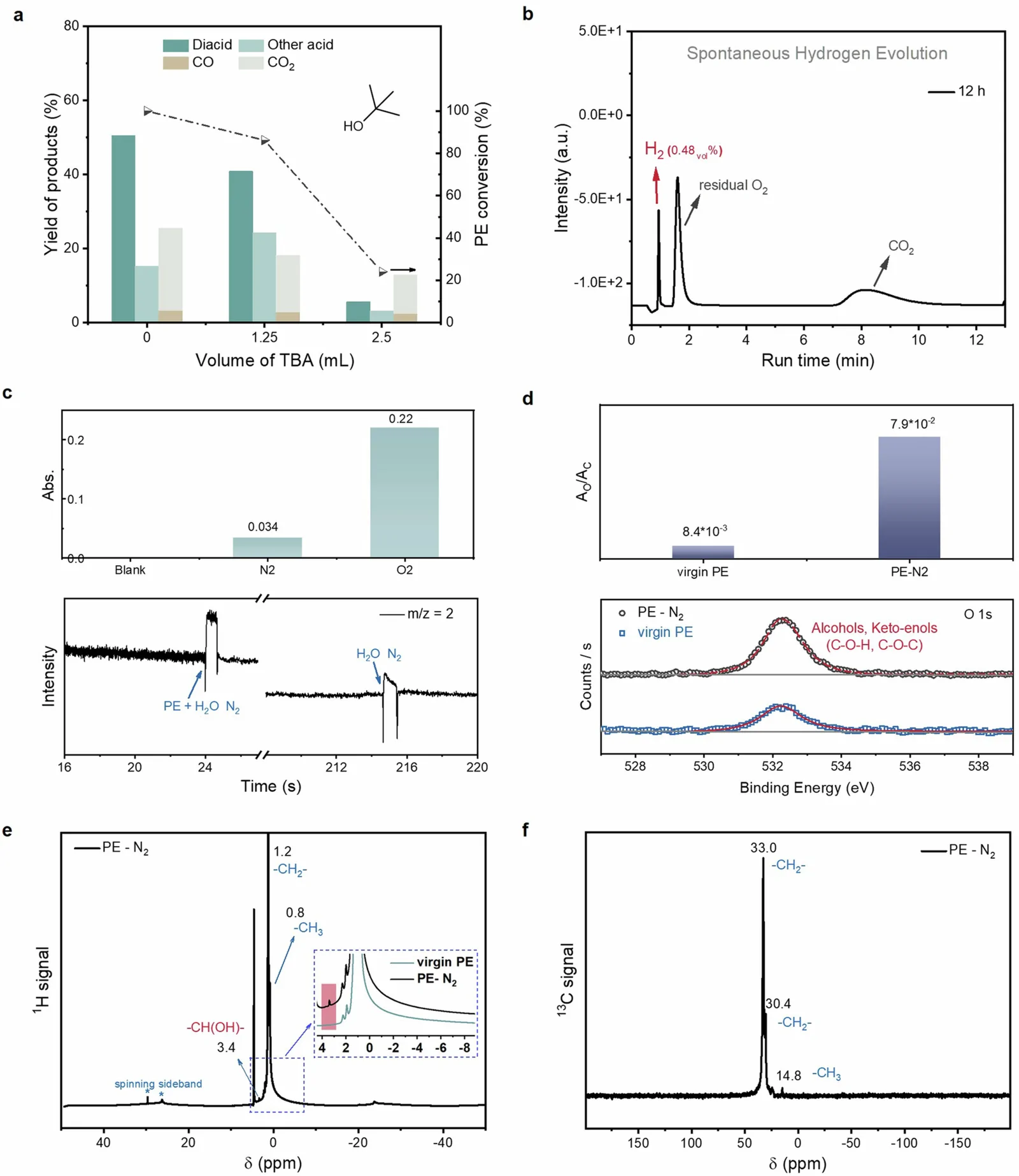
Role of hydroxyl radicals in the catalyst-free oxidative degradation of PE. **a**, The yield of products and PE conversion under different amounts of *tert*-butyl alcohol. PE (0.2 g), 5 ml of deionized water and *tert*-butyl alcohol (0, 0.5 or 2.5 ml) were loaded into the autoclave. **b**, The GC–thermal conductivity detection of gas products after 12 h. PE (0.2 g) and 5 ml of deionized water were loaded into the autoclave. **c**, UV absorbance of the solution (0.5 ml 2 mM TMB/DMSO + 5 ml H_2_O) under N_2_ and O_2_ after 12 h at 125 °C and the gas-phase online MS of the PE + H_2_O and pure H_2_O systems. **d**, X-ray photoelectron spectroscopy O 1s spectra of virgin PE and PE residual solid after reaction under N_2_ for 12 h. **e**,**f**, The solid-state ^1^H-NMR spectrum (**e**) and solid-state ^13^C NMR spectrum (**f**) of virgin PE and recovered PE-N_2_ residue. To corroborate •OH generation under N_2_, TMB probe experiments showed a UV–Vis absorbance of 0.034 under N_2_ versus 0.22 under O_2_ after 12 h, confirming interfacial •OH production independent of oxygen. Gas-phase MS detected enhanced H_2_ (*m*/*z* = 2) in the PE + H_2_O headspace relative to pure H_2_O, consistent with the proposed reduction half-reaction. X-ray photoelectron spectroscopy O 1s analysis revealed an O/C ratio of 7.9 × 10^−2^ for recovered PE versus 8.4 × 10^−3^ for virgin PE, evidencing •OH-mediated surface oxidation. Solid-state NMR further identified a new resonance at 3.4 ppm assignable to –CH(OH)– intermediates.

The critical role of •OH in the degradation process was validated by adding *tert*-butyl alcohol (a known •OH scavenger), which substantially reduced both PE conversion and diacid yield (Extended Data Fig. 3a). Replacing H_2_O with D_2_O revealed a kinetic isotope effect (*k*_H_/*k*_D_ = 7), confirming water-derived •OH formation as the rate-determining step (Fig. 2c). These findings align with previous reports of •OH generation at microdroplet interfaces^39,42^. Meanwhile, hydrogen gas detected in gaseous products (0.48 vol%; Extended Data Fig. 3b) may originate from H^+^ reduction coupled to •OH generation^29^. This concentration remains below the lower explosive limit, posing no safety risk.

**Extended Data Fig. 4:**
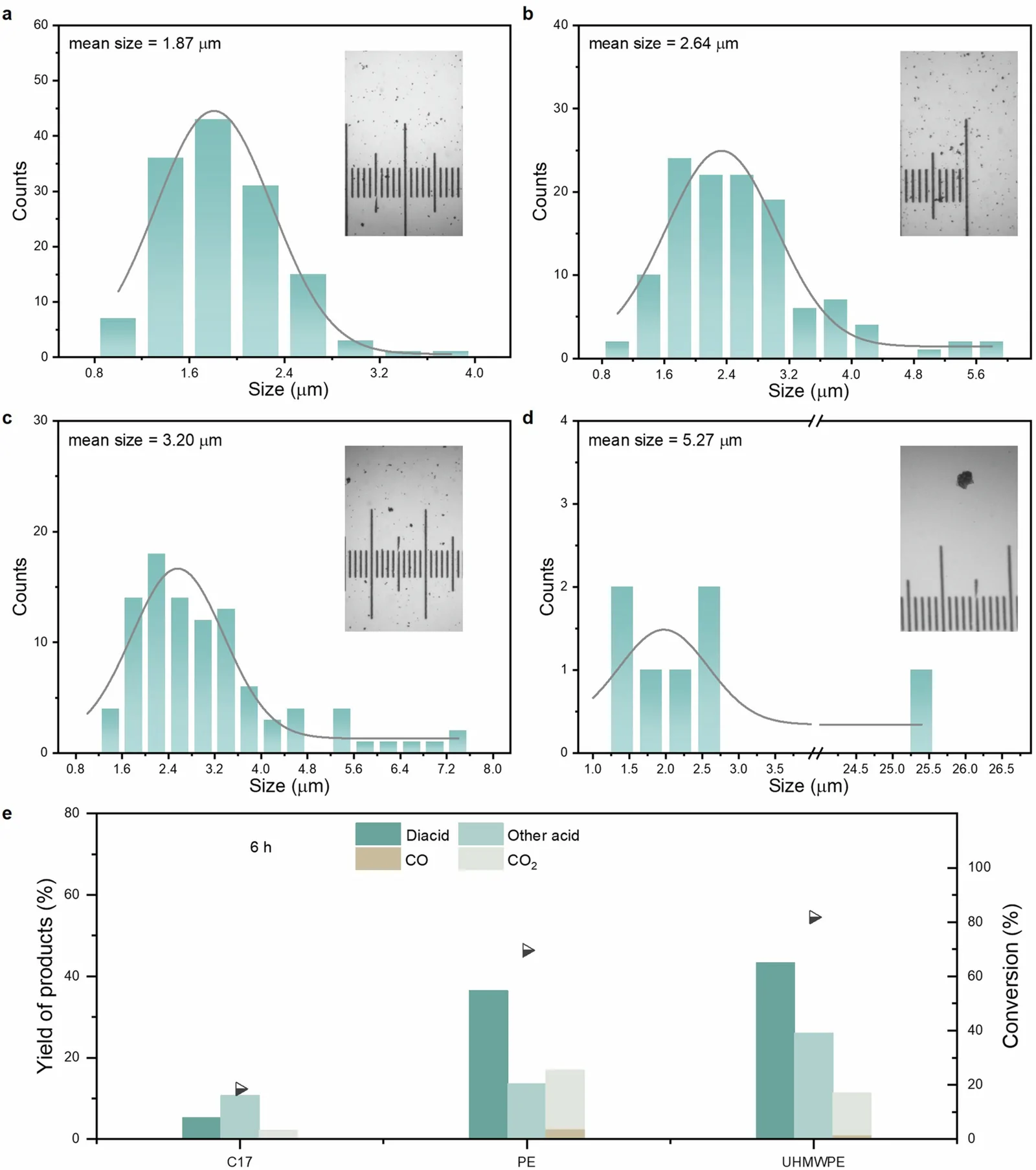
Optical microscopy characterization of PE microdroplets and chain length effect on interfacial reaction. **a**–**d**, Particle size distributions of PE droplets collected by intermediate sampling at 125 °C under 2 MPa O_2_ with 600 rpm stirring and after cessation of stirring and stepwise cooling to 110 °C, 90 °C and 70 °C. **e**, The conversion and product yields after 6 h of oxidation of substrates with different carbon chain lengths. 0.2 g PE/heptadecane/UHMWPE and 5 ml of deionized water were loaded into the autoclave.

Further EPR analysis of samples derived from deoxygenated water (125 °C, 2 MPa N_2_) also confirmed •OH signals regardless of PE presence (Fig. 2d and Extended Data Fig. 3c–f), indicating that •OH generation requires neither O_2_ nor substrate. Notably, the reaction mixture became turbid with PE on sampling, exhibiting Tyndall scattering and a zeta potential of −28.3 mV (25 °C), alongside intensified •OH signals (Fig. 2d). Also, post-cooling transparency suggested reversible PE dispersion into microdroplets during heating and stirring (Supplementary Video 3). Optical microscopy confirmed the formation of PE particles with a narrow size distribution and an average diameter of 1.87 μm (Fig. 2e and Extended Data Fig. 4a–d), aligning with microdroplet sizes previously reported to generate •OH (refs. ^6,33^). Furthermore, testing substrates with different carbon chain lengths revealed that longer carbon chains enhanced degradation (ultrahigh-molecular-weight polyethylene (UHMWPE) > PE > heptadecane; Extended Data Fig. 4e), probably because of enhanced interfacial water orientation and stronger electric fields^41^. This system enables thermoplastic recycling by means of polymer–water interfaces that promote radical generation while limiting overoxidation on diacid dissolution.

**Fig. 3:**
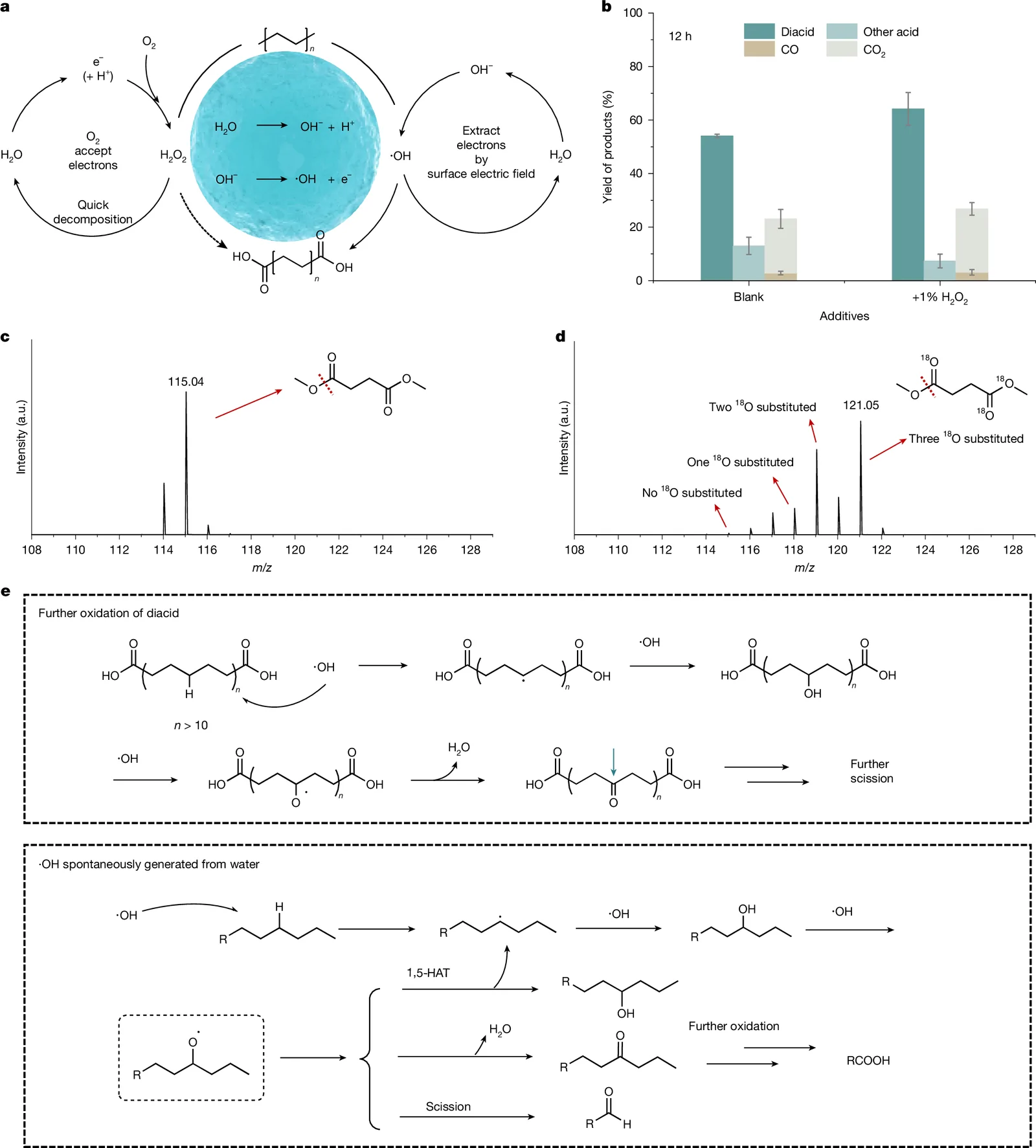
Time course and mechanisms of the catalyst-free PE oxidation. **a**, Mechanism diagram of the spontaneous oxidation of PE to diacid on the surfaces of microdroplets. **b**, Comparison of product yields between the PE oxidation system and the PE oxidation system with an extra 1% H_2_O_2_. **c**,**d**, GC–MS of esterified succinic acid generated in H_2_^16^O and H_2_^18^O under ^16^O_2_ after 12 h. **e**, Mechanism diagram of the further oxidation of diacid and the spontaneous oxidation of PE.

**Extended Data Fig. 5:**
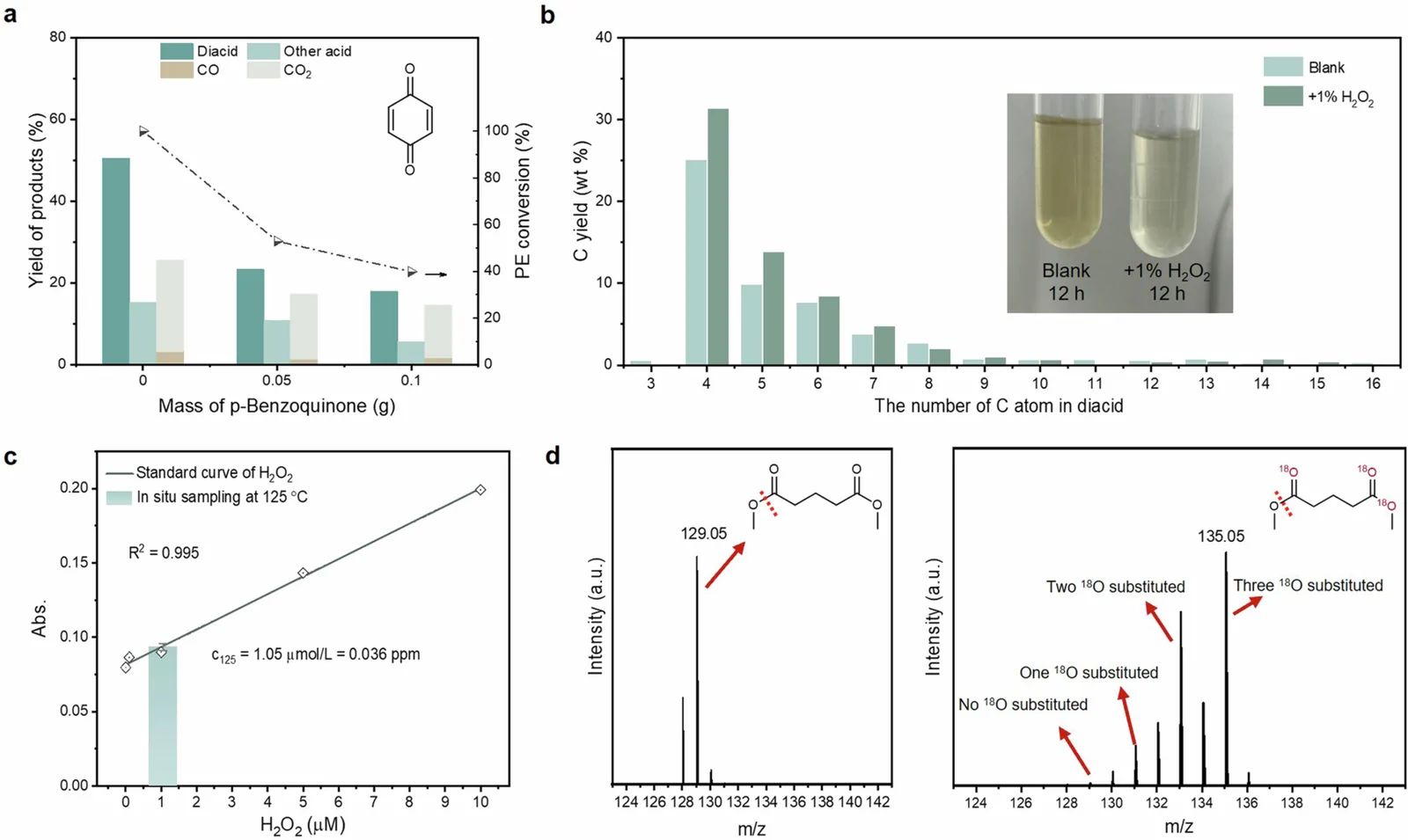
Role of O_2_, water and H_2_O_2_ in the catalyst-free oxidative degradation of PE. **a**, The yield of products and PE conversion under different amounts of benzoquinone. PE (0.2 g), 5 ml of deionized water and benzoquinone (0, 0.02, 0.05 or 0.10 g) were loaded into the autoclave. **b**, Distribution of diacid generated in pure water and water with an extra 1% H_2_O_2_ and the colour of the solution after the reaction in pure water and water with an extra 1% H_2_O_2_ after 12 h. **c**, The concentration of in situ-generated H_2_O_2_ as measured by the assay kit (only for H_2_O_2_). This value is very likely underestimated because H_2_O_2_ decomposes rapidly at 125 °C. **d**, The GC-MS analysis of the esterified glutaric acid in H_2_^16^O and H_2_^18^O. The observed trend mirrors that of esterified succinic acid, underscoring the pivotal role of water in PE oxidation and the synergistic incorporation between water and oxygen.

Notably, the two-stage PE degradation kinetics under O_2_ (Fig. 2f) correspond to the melting–dispersing stage and the accelerated degradation stage that occurs after the complete melting of PE. In the presence of oxygen, the second stage follows first-order kinetics, indicating that radical generation depends on PE concentration. This aligns with our hypothesis that •OH production occurs mostly at the PE–water interface. Confocal fluorescence imaging further confirmed the localization of a charged fluorescent probe along oil-in-water microdroplet interfaces (Fig. 2b), evidencing the critical role of the water–oil interfacial electric fields^37^. It should be noted that negligible PE degradation occurred in the absence of O_2_ (Fig. 2f), although •OH signals were detected through EPR under N_2_, confirming the essential role of O_2_. Further, benzoquinone quenching of superoxide radicals (•O_2_^−^) suppressed PE conversion (Extended Data Fig. 5a), verifying •O_2_^−^ participation in the oxidative degradation of PE. This indicates a sequential process in which interfacial electric fields generate •OH and e^−^ from H_2_O (Fig. 3a). Then O_2_ reacts with e^−^ to form •O_2_^−^, which further protonates to H_2_O_2_, sustaining oxidation of PE (ref. ^42^). In N_2_, on the other hand, accumulated electrons from OH^−^ consumption prevent sustained •OH production, halting degradation.

To further validate our hypothesis, we added 1% H_2_O_2_ and shortened the reaction time to 12 h, inducing a distinct colour change (Extended Data Fig. 5b) and boosting diacid yield by 10% through reducing the content of other acids (Fig. 3b). Increased short-chain diacid production also confirms accelerated oxidation driven by in situ H_2_O_2_ or •OH. However, rapid H_2_O_2_ decomposition at 125 °C precludes its role as the primary oxidant in PE oxidation. In situ sampling under pure water and 2 MPa O_2_ detected only 0.036 parts per million (ppm) H_2_O_2_ (Extended Data Fig. 5c). Although minimal, this observation directly verifies its transient formation. The synergistic roles of O_2_ and water were further confirmed by H_2_^18^O isotope labelling experiments (Fig. 3c,d and Extended Data Fig. 5d), with GC–MS analysis of esterified succinic acid showing a clear +6 *m*/*z* base peak shift in the presence of H_2_^18^O, indicating water-derived oxygen atoms in most products. Although previous studies report that microdroplet H_2_O_2_ originates from O_2_ (refs. ^29,31^), this still aligns with our system given rapid H_2_O_2_ decomposition. Instead, our results highlight water-derived •OH as the dominant active oxygen species. Furthermore, detection of single- and double-^18^O fragment ions confirms concurrent O_2_ and H_2_O incorporation. Critically, absent all-^16^O fragments further underscore the indispensable mechanistic role of H_2_O.

**Extended Data Fig. 6:**
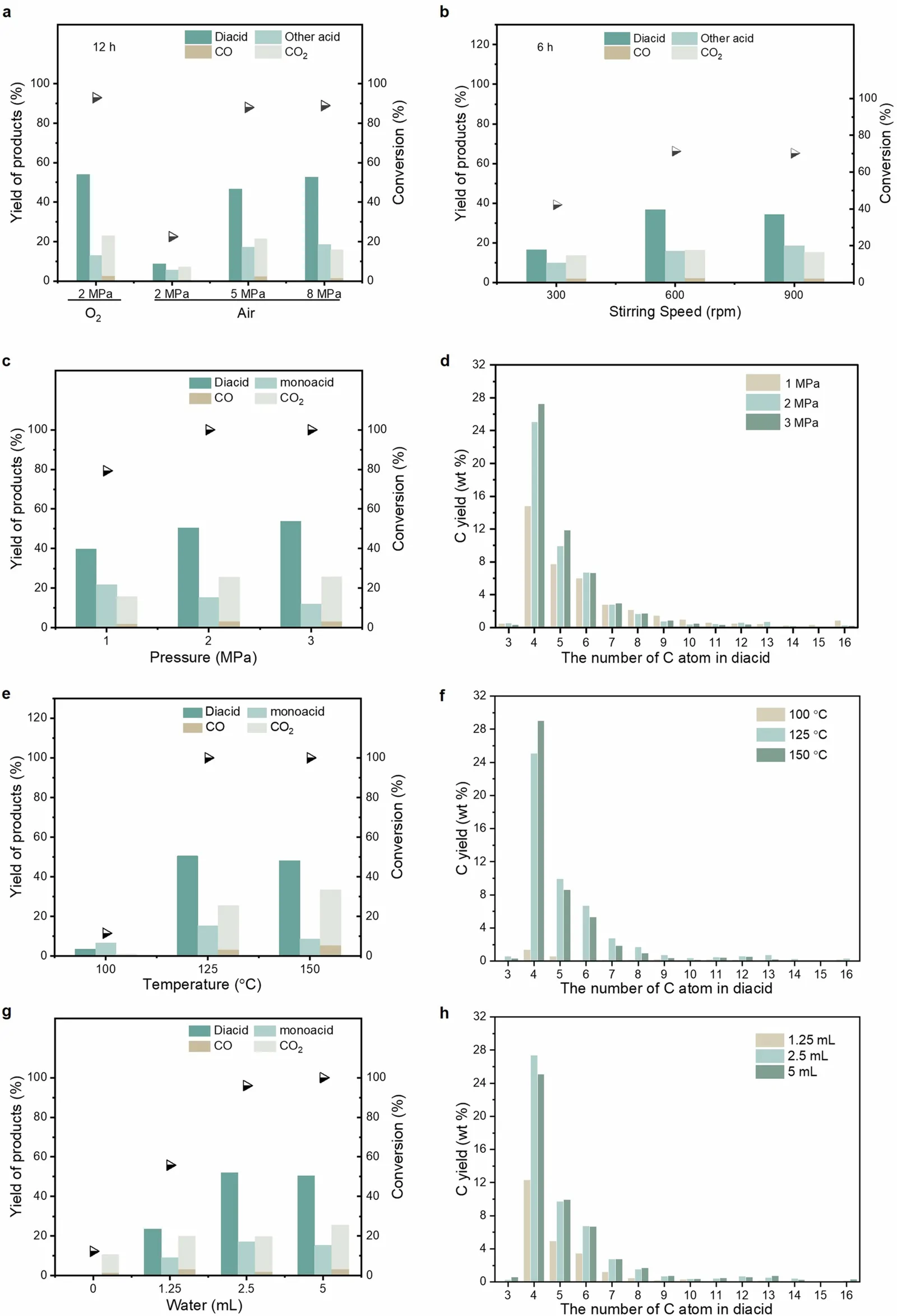
Effects of reaction conditions on PE oxidation and product distribution. **a**, Product yield and PE conversion under different atmospheres. **b**, Product yield and PE conversion at different stirring speeds under 2 MPa O_2_ and 125 °C after 6 h. At 600 rpm, droplet formation and interfacial residence time are balanced, giving the highest conversion and diacid selectivity. Slower stirring limits droplet/fragment generation, whereas faster stirring (900 rpm) promotes convective stripping of partially oxidized monoacids and oligomers into bulk water, reducing diacid selectivity. **c**,**d**, Product yield and diacid distribution at different O_2_ pressures at 125 °C after 12 h. **e**,**f**, Product yield and diacid distribution at different temperatures under 2 MPa O_2_ after 12 h. **g**,**h**, Product yield and diacid distribution at different water volumes after 12 h.

PE degradation was investigated under varied conditions by adjusting temperature, stir speed and oxygen pressure to control oxidation and optimize diacid yield (Extended Data Fig. 6). Optimal performance was achieved at 125 °C and 2 MPa O_2_ with vigorous stirring (600 rpm), which promotes polymer melting and interfacial dispersion, thereby enhancing •OH generation while avoiding excessive overoxidation. Reinforcing the role of H_2_O, sufficient volume proved essential: diacid yields dropped sharply at low water levels, indicating that optimal water-to-oil ratios and direct liquid water–molten PE contact drive efficient interface-dependent oxidation.

**Extended Data Fig. 7:**
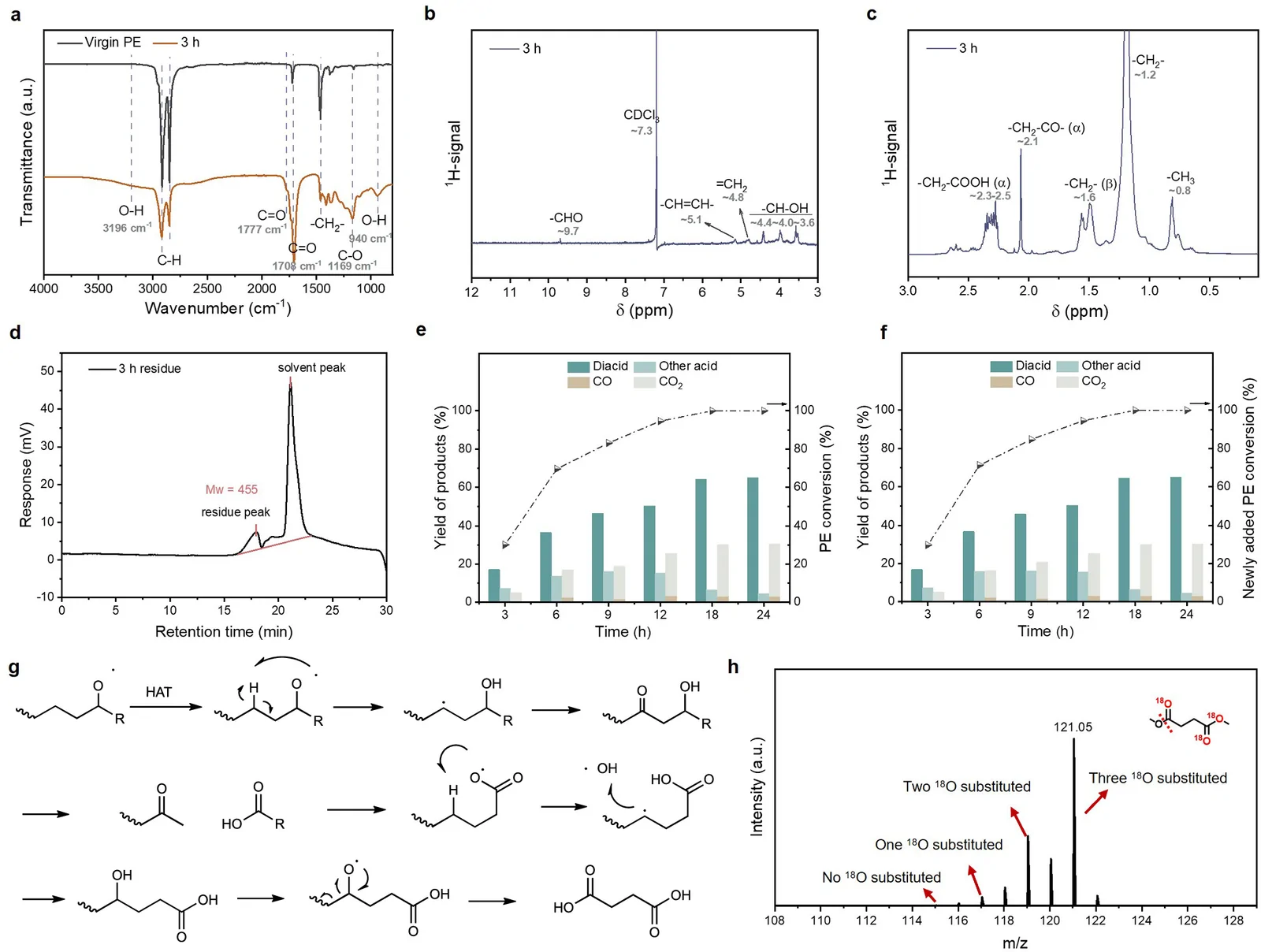
Characteristics of PE degradation and products yield over time. **a**, The infrared spectrum of virgin PE and the solid residue after 3 h. **b**,**c**, The ^1^H-NMR spectrum of the solid residue after 3 h. **d**, GPC of the residue after 3 h of oxidation of PE. After 3 h of reaction, the molecular weight of the residual polymer was only 455 g mol^−1^ (virgin PE: 4,700 g mol^−1^), indicating that the degradation efficiency of PE is considerable. **e**, Evolution of the yield of products and the conversion of PE. **f**, Evolution of product yields and conversion of newly added PE in the 24 h post-reaction solution. The products derived from the initial PE were excluded when calculating the yields for the second-round reaction, indicating that the presence of products will not act as a radical sink to inhibit the process. **g**, The mechanism of the high selectivity of C_4_–C_6_ diacids (taking succinic acid as an example). The preferential formation of C_4_–C_6_ diacids occurs owing to low-strain, kinetically favoured 5-/6-membered cyclic peroxide intermediate. **h**, GC-MS of esterified succinic acid generated in H_2_^18^O under ^16^O_2_ after 3 h. This indicates that •OH is always the main inducer of the whole reaction. The oxidized polymer shows several carbonyl bands: C=O stretching at 1,777 cm^−1^ (anhydride), 1,735 cm^−1^ (aldehyde) and 1,708 cm^−1^ (carboxylic acid/ketone), alongside a broad O–H band at 3,196 cm^−1^ and O–H out-of-plane bending at 940 cm^−1^, collectively confirming the introduction of oxygenated functionalities along the polymer backbone. The C–O stretching band at 1,169 cm^−1^ further indicates the formation of alcohol intermediates, whereas attenuation of the –CH_2_– scissoring band at 1,462 cm^−1^ relative to virgin PE is indicative of backbone oxidation and chain scission. These FT-IR assignments are fully consistent with the ^1^H-NMR result. Notably, the olefinic signals are of very low intensity, indicating that it is a minor pathway.

Gel permeation chromatography with refractive index detection (GPC-RI) revealed a notable molecular weight reduction of PE residues after 3 h, whereas Fourier transform infrared spectroscopy and ^1^H-NMR showed emerging carbonyl peaks and weakened C–H stretches (Extended Data Fig. 7a–d), confirming the introduction of oxygenated functional groups to inner carbon chains. Time-course analysis indicated near-complete PE conversion at 12 h, with a diacid yield of 50.5%. Extending to 18 h increased diacid yields while reducing monocarboxylic and unsaturated dicarboxylic acids. Product yields plateaued beyond 24 h (Extended Data Fig. 7e). When fresh PE was added to the post-reaction mixture, followed by recharging with O_2_ and continuing the reaction for another 24 h, the product distribution showed a similar profile (Extended Data Fig. 7f), indicating that the presence of products does not inhibit the process. Notably, C_4_–C_6_ diacids exhibited higher selectivity than longer-chain analogues across all tested reaction conditions, consistent with 1,5-hydrogen atom transfer (HAT) pathways proceeding through thermodynamically favoured 6–8-membered transition states (Extended Data Fig. 7g). As the reaction progressed, longer-chain diacids and unsaturated dicarboxylic acids underwent further oxidation and cleavage, forming short-chain diacids as the major products, evidenced by rising C_4_–C_6_ yields alongside CO_2_ evolution. Further oxidation of short-chain diacids was minimal, as demonstrated by control experiments using commercial diacids with chain lengths of C_4_–C_8_, confirming that the increased CO_*x*_ yields at higher temperatures originate from the initial radical-mediated scission pathways rather than the secondary overproduction of CO_2_.

By contrast, longer-chain diacids could undergo further oxidation. We propose that electron-withdrawing carboxyl groups direct •OH to abstract H from electron-rich distal C–H bonds, initiating sequential radical reactions (Fig. 3e) that form unsaturated diacids with central ketones-observed intermediates. Subsequent oxidative cleavage yields shorter-chain diacids. Crucially, this cleavage occurs only for chains >C_12_, indicating that ketone functionalities (for example, 4-ketoheptanedioic acid; Extended Data Fig. 1b) must form before diacid generation, as inferred from product distributions. Collectively, we propose a catalyst-free PE oxidation pathway mediated by interfacial •OH (Fig. 3e). Initially, interfacial •OH abstracts hydrogen atoms from the PE chains, generating alkyl radicals. Alkyl radicals are then captured by •OH to produce alcohol intermediates (Extended Data Fig. 7h). Further •OH attack forms alkoxyl radicals that undergo β-scission or HAT, producing aldehyde and ketone intermediates. These intermediates are further oxidized into carboxylic acids, completing the degradation process.

**Fig. 4:**
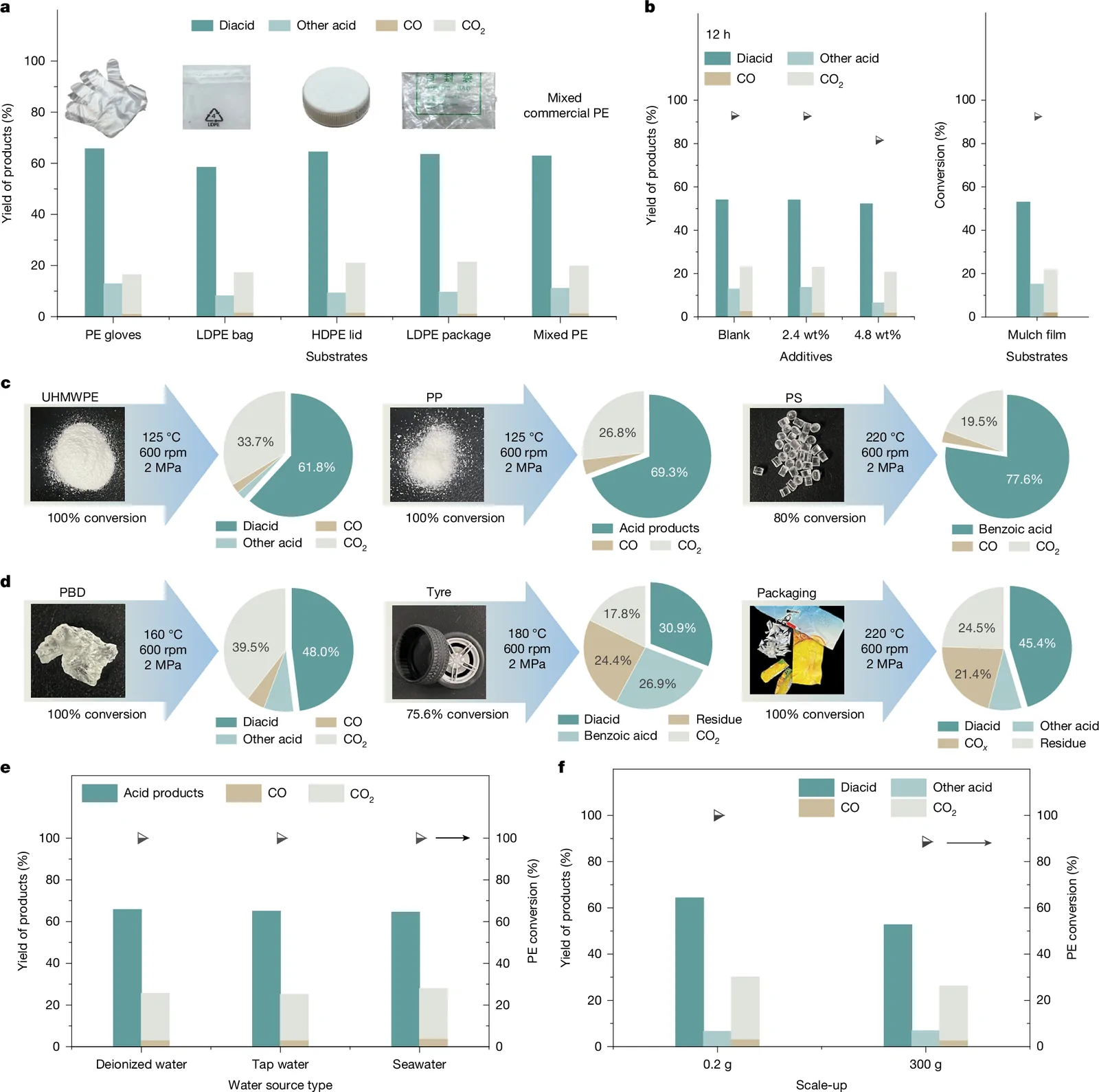
The substrate expansion and application of the catalyst-free PE oxidation strategy. **a**–**d**, Performance evaluation using different commercial PE: PE gloves, LDPE bag, HDPE lid, LDPE package and mixed commercial PE (**a**); PE under different conditions of additives (2.4 wt%: manually add 0.5 wt% butylated hydroxytoluene (BHT), 0.5 wt% bis(2,2,6,6-tetramethyl-4-piperidyl)sebacate (HALS), 0.4 wt% Irganox 1010 and 1 wt% *N*-(1,3-dimethylbutyl)-*N*′-phenyl-*p*-phenylenediamine (6PPD); 4.8 wt%: twice the manually added amount of *1; mulch film: agricultural film containing HALS) (**b**); different polyolefins: UHMWPE, PP, PS (**c**); (**d**) rubber products: PBD, real-life tyre and multilayer packaging films (**d**). **e**, Performance evaluation in seawater matrices. **f**, The scale-up experiment on catalyst-free oxidation of PE.

**Extended Data Fig. 8:**
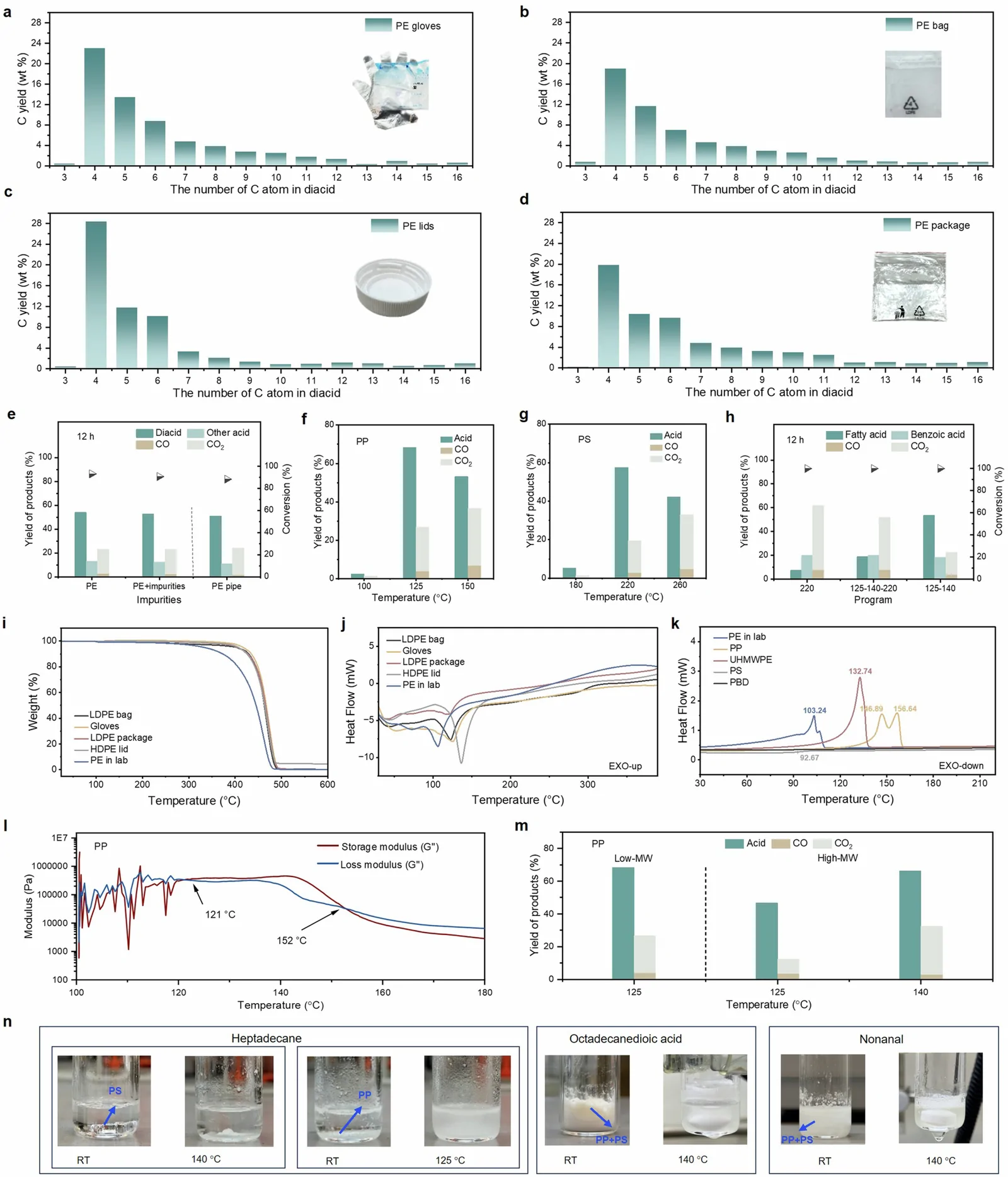
Application to commercial, mixed polyolefins and impurity-containing PE. **a**–**d**, The diacid distribution of commercial PE gloves (**a**), PE bag (**b**), PE lids (**c**) and PE package (**d**) under 125 °C and 2 MPa O_2_ after 12 h. **e**, The yield of products and PE conversion under different conditions of inorganic impurities (manually add: 5 wt% TiO_2_ + 5 wt% CaCO_3_ + 0.1 wt% ZnO; PE pipe: containing TiO_2_). **f**,**g**, Product yields from PP (**f**) and PS (**g**) oxidation at different temperatures under 2 MPa O_2_. **h**, The product yields from mixed polyolefins (PE, PP, PS) under different heating protocols. In mixed polyolefin blends, PE melts first on heating and undergoes partial oxidative degradation to generate low-molecular-weight wax-like oligomers, which wet and partially swell the surfaces of higher-melting components such as PP and PS, facilitating their co-dispersion into the aqueous phase as polymer-in-water microdroplets under mechanical agitation. **i**,**j**, Thermogravimetric curves (**i**) and differential scanning calorimetry curves (**j**) of commercial PE and PE standard sample in the lab. **k**, Differential scanning calorimetry curves of various polymers in the lab. **l**, Dynamic mechanical analysis of PP. **m**, The yield of products of low-molecular-weight PP and high-molecular-weight isotactic PP oxidation under different temperatures after 12 h. PP with different molecular weights and tacticity require different operating temperatures for complete conversion, owing to their differing thermal softening behaviour. For higher-molecular-weight isotactic PP, a higher reaction temperature is required to achieve high conversion. **n**, Photographs showing the interaction of PS and PP with oxidation-product analogues at room temperature (RT) and 140 °C/125 °C.

This catalyst-free strategy also demonstrated broad applicability for the degradation of post-consumer polyolefins. Post-consumer polyolefin materials were roughly cut and subjected to degradation without any catalyst. Consumer-grade PE waste including gloves, low-density polyethylene (LDPE) bags, high-density polyethylene (HDPE) caps and their mixtures achieved 100% conversion with diacid yields exceeding 60 wt% (Fig. 4a and Extended Data Fig. 8a–d). We also evaluated the effects of excess antioxidants, hindered amine light stabilizers (HALS) and representative inorganic impurities commonly found in commercial plastics (Fig. 4b, Extended Data Fig. 8e and Supplementary Tables 4 and 5). Under typical formulation loadings, these additives had no detectable effect on either the reaction conversion or diacid yield, underscoring the robustness of the process. The process was extended to other polymers by tuning the temperature to match their dispersion behaviour (Fig. 4c,d). Because the oxidation is exothermic, the operating temperature can be effectively set near or below the melting point of the polymer (Extended Data Fig. 8i–m), as thermal softening combined with mechanical agitation ensures sufficient interfacial contact to initiate •OH-mediated oxidation. For example, UHMWPE was fully converted at 125 °C, yielding 61.8% diacids. Polypropylene (PP) and polystyrene (PS) were selectively converted to acetic acid and benzoic acid at 125 °C and 220 °C, respectively, driven by the reactivity of tertiary sp^3^ carbons (Extended Data Fig. 8f,g). The method also degraded challenging rubber-based materials and multilayer packaging films (Fig. 4d). Waste polybutadiene (PBD) processed at 160 °C yielded predominantly succinic acid, whereas real tyres processed at 180 °C produced 33.9% diacid and 26.9% benzoic acid, along with 24.4% inorganic solids, despite the presence of vulcanizers that would severely poison conventional metal or acid catalysts. Post-consumer packaging bags were similarly processed at 220 °C for 12 h, yielding diacids as the dominant products (45.4%), with the metallized barrier layer recovered as an Al-rich solid residue confirmed by X-ray diffraction. Mixed real-world polyolefin waste streams were also co-processed under optimized conditions (Extended Data Fig. 8h,n), achieving satisfactory conversion and diacid yields, further confirming the practical applicability of the approach. The system also showed strong compatibility with natural water sources such as tap water and seawater (Fig. 4e), indicating halogen resistance. Biodegradable products and minimal infrastructure demands make it particularly suitable for marine waste treatment on remote islands, offering a sustainable vision for global waste management. Scalability was confirmed in a 5-l reactor (300 g PE, 3 l H_2_O, 2 MPa O_2_), achieving 89% conversion with saturated diacid yields of more than 52% after 48 h (Fig. 4f).

**Extended Data Fig. 9:**
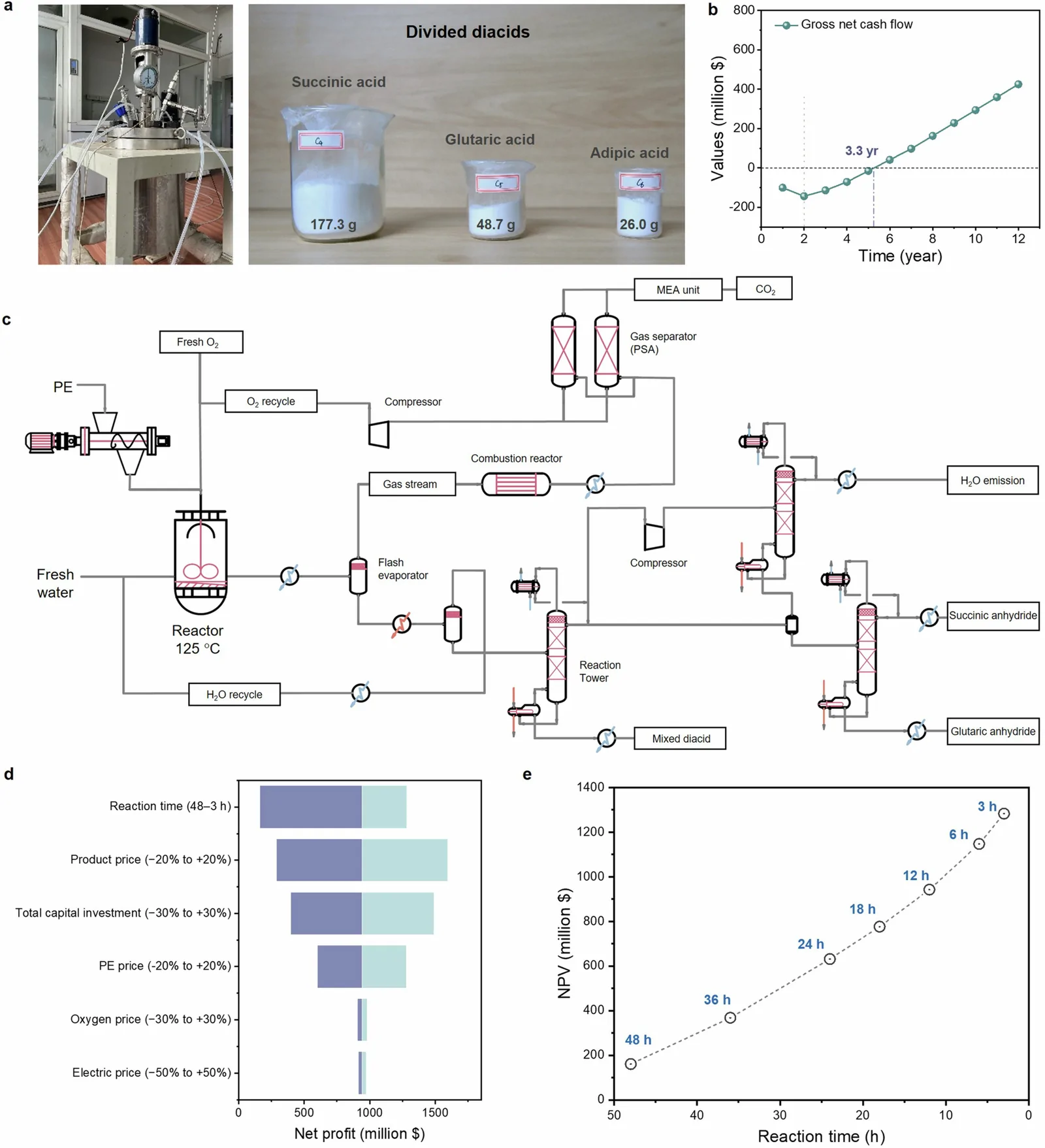
Scale-up experiments and TEA of catalyst-free PE oxidation. **a**, Reactor and separated products of the scale-up experiment. **b**, The static payback period of this technology model (60 kt). The construction period was set to 2 years. **c**, Proposed process flow diagram to produce diacid from waste PE through catalyst-free oxidation. **d**, One-way sensitivity analysis of NPV. **e**, Sensitivity analysis of NPV as a function of reaction time.

Techno-economic analysis underscores the robust economic viability of this catalyst-free polyolefin upcycling process (Extended Data Fig. 9). The PE feedstock was conservatively priced at roughly US $0.83 per kg, reflecting sorting, cleaning and transport costs^50^. The reactor effluent undergoes cooling and flashing to segregate the gaseous (O_2_, CO_2_ and CO) and liquid phases. CO is combusted for energy recovery with monoethanolamine (MEA)-based flue gas capture, whereas process water is reclaimed for internal recycling. The heavier liquid fraction is upgraded through dehydration and fractional distillation to yield diacids and their corresponding anhydrides. At a representative capacity of 60 kt year^−1^, the plant requires a total capital investment of $144 million (Supplementary Tables 6–10), yielding an annual after-tax net profit of $72.1 million and a payback period of about 3.3 years without government subsidies (Extended Data Fig. 9b). The industrial model assumes a 12 h residence time in a continuous plug-flow reactor, using a heated screw feeder and distributed gas injection to maximize interfacial renewal and radical flux. Sensitivity analysis (Extended Data Fig. 9d,e) confirms that net present value (NPV) remains positive even if the residence time extends to 48 h. The break-even capacity is approximately 9.0 kt year^−1^, demonstrating operational resilience to feedstock volatility and supporting decentralized deployment at sub-nominal scales. Although product pricing and capital expenditure remain the main drivers of profitability, the strong influence of residence time on NPV highlights the importance of reactor engineering in minimizing capital intensity.

**Extended Data Fig. 10:**
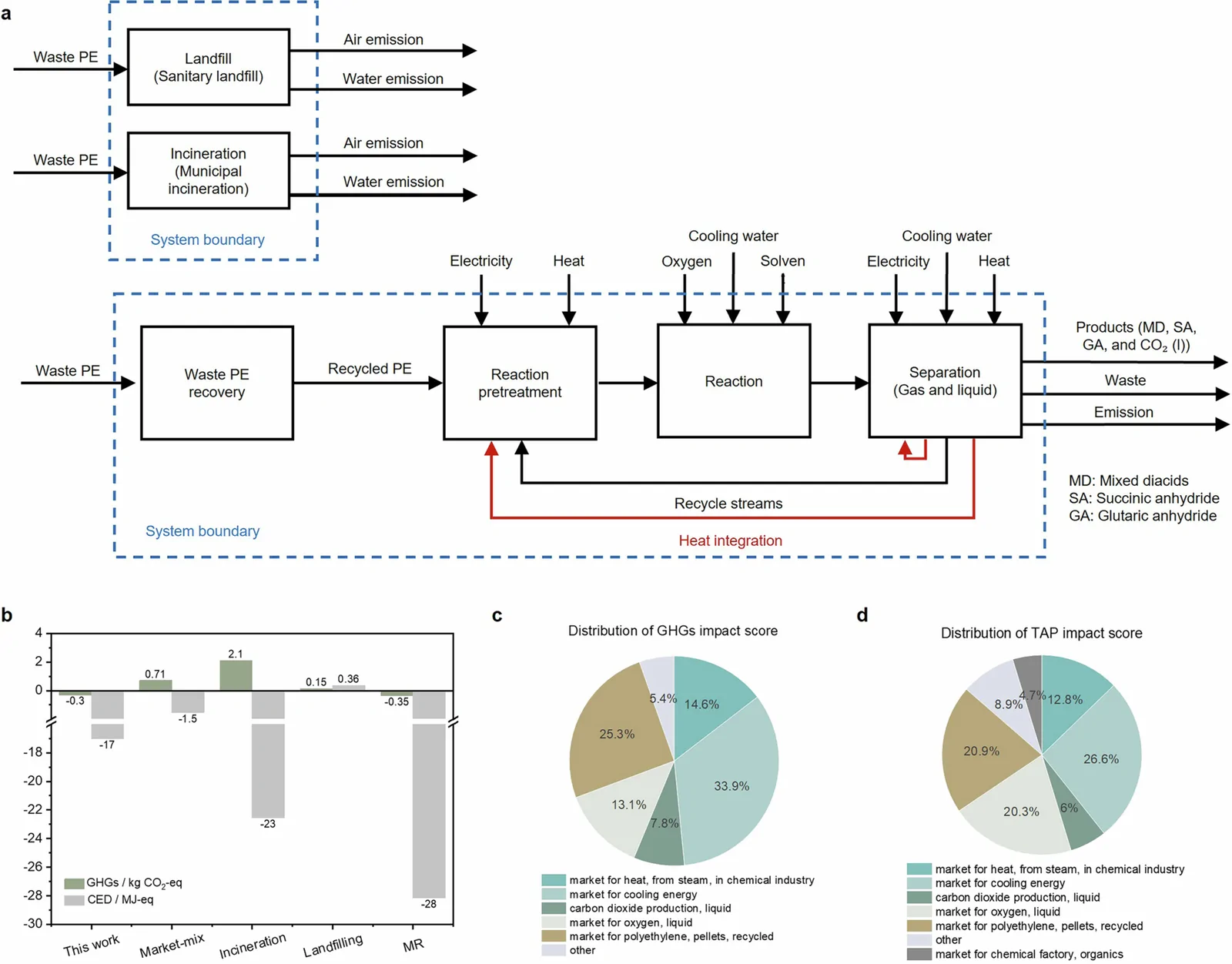
LCA of waste PE upcycling. **a**,**b**, System boundary for conventional waste PE treatment scenarios (sanitary landfilling and municipal incineration) (**a**) and the proposed chemical upcycling route (**b**). **c**, GHG emissions and CED for the treatment of 1 kg of plastic through: the proposed upcycling technology, incineration, landfilling, the conventional market-mix technology and MR. **d**, Contribution analysis of the upcycling technology to GHG emissions and TAP.

Life-cycle assessment benchmarks environmental performance against conventional PE waste management routes and mechanical recycling. Using a gate-to-gate framework (Extended Data Fig. 10a), the analysis shows that our oxidative upcycling is a net carbon sink (greenhouse gas emissions: −0.30 kg CO_2_-eq), outperforming incineration (2.11 kg CO_2_-eq) and landfilling (0.15 kg CO_2_-eq) while matching mechanical recycling (−0.35 kg CO_2_-eq), with substantial cumulative energy savings (Extended Data Fig. 10b). End-point indicators for human health, ecosystem quality and resource scarcity consistently favour the upcycling route over incineration and landfilling (Supplementary Tables 11–13).

## Conclusion

By activating radical reactions using electric-field manipulation at water–molten plastic microdroplet interfaces, we report a catalyst-free oxidative cleavage strategy for inert polyolefins and diverse plastic wastes using only water and O_2_ under mild conditions (125 °C, 2 MPa). PE is fully converted into biodegradable short-chain dicarboxylic acids (69% yield, predominantly succinic acid) without any microplastic residues through a distinct •OH-mediated mechanism, diverging from conventional alkane aerobic oxidation systems. This approach exhibits exceptional substrate compatibility, tolerance to contaminants and economic efficiency. Further, the minimal infrastructure requirements demonstrate facile scalability (300 g level), making the process particularly suitable for pollution-vulnerable regions, including developing nations, remote islands and open-ocean ecosystems. Our work pioneers industrial application of microdroplet chemistry and holds promise in revolutionizing the conventional industrial oxidation processes beyond plastic upcycling.

## Methods

### Materials

PE (*M*_n_ = 1,700 Da, *M*_w_ = 4,700 Da by GPC) was purchased from Sigma-Aldrich. PP (*M*_n_ = 5,000 Da), UHMWPE (*M*_n_ = 6,000,000 Da) and 3,3′,5,5′-TMB were purchased from Macklin. (Trimethylsilyl)diazomethane (TMS-CHN_2_), PBD and all of the standard diacids were purchased from Aladdin. 4-Carboxyphenylboronic acid (4-CPB) was purchased from Shanghai Meryer Scientific. CH_3_OH (as received), ether (as received) and 30% H_2_O_2_ (as received) were purchased from Sinopharm Chemical Reagent. PS (as received) was purchased from Shanghai Titan Scientific. H_2_^18^O (97% ^18^O) was purchased from Shanghai Xinbo Industrial. Deionized water with the specific resistance of 18.2 MΩ cm was used in all experiments.

### Characterization

The molecular weight of the polymers was recorded using an Agilent PL-GPC 220 system. 1,2,4-trichlorobenzene (TCB) was used as mobile phase at 150 °C. High-temperature gel permeation chromatography (HT-GPC) measurements were calibrated using narrow PS standards. The effective calibration range of the system spans approximately 500 to 1 × 10^7^ g mol^−1^. ^1^H-NMR, ^13^C-NMR and HSQC were performed on a Bruker AVANCE III 500. Fourier transform infrared (FT-IR) spectra were recorded on a Nicolet Nexus 470 in the spectral range 400–4,000 cm^−1^ using the KBr disc method. Ultraviolet–visible (UV–Vis) spectra were recorded on a Hitachi UH5300 in the spectral range 190–1,100 nm. EPR signals were carried out on a Bruker A300 X-band EPR spectrometer. The radical scavenger used was 5,5-dimethylpyrroline-*N*-oxide (DMPO). The H_2_O_2_ concentration was measured by a catalase assay kit (Beyotime Biotech) on a microplate reader (Thermo Fisher Scientific Varioskan LUX). Confocal fluorescence detection was performed on a Zeiss LSM 980 with Airyscan. We conducted thermal analysis using a METTLER-TOLEDO TGA/DSC 1 or an equivalent synchronous thermal analyser. All measurements were performed under a nitrogen atmosphere with a heating rate of 10 K min^−1^. We carried out proximate analysis using a 5E-MAG6700 Fully Automatic Industrial Analyzer. Before testing, the commercial PE samples were cooled with liquid nitrogen and ground into powder.

### Catalyst-free PE oxidation

This one-pot reaction was performed in the Parr autoclave (Anhui CHEM^N^ Instrument) equipped with an electronic pressure gauge. PE (0.2 g) and 5 ml of deionized water were loaded into the autoclave. The sealed reactor was purged using alternating vacuum and oxygen cycles (3×), then charged to 2 MPa with oxygen and heated to 125 °C at a heating rate of 5 °C min^−1^ under stirring at 600 rpm. After the reaction, gas products were collected by a 0.5-l Teflon bag. Then, the liquid products were collected and the autoclave was washed using 5 ml of methanol. Finally, the product dissolved in liquid phase and unreacted solid residue is separated by centrifugation. Unless otherwise specified, all other reaction conditions are based on this. We noted a similar work during our revision. Although both studies share the same phenomenon of catalyst-free oxidative upcycling of PE, the reaction mechanisms and conclusions differ substantially^51^.

### Applicability of other polymers oxidation

The post-consumer plastics were derived from PE gloves, LDPE bags, HDPE caps, LDPE packaging and tyres, which were cut into small pieces without any other operation. Then, 0.2 g of post-consumer polyolefin and 5 ml of deionized water were loaded into the autoclave. The sealed reactor was charged to 2 MPa with oxygen and heated to 125 °C at a heating rate of 5 °C min^−1^ under stirring at 600 rpm.

### Measurements for •OH detection

4-CPB generates 4-hydroxybenzoic acid (4-HB) under the action of •OH/H_2_O_2_. 0.60 g of 4-CPB and 5 ml of H_2_O were added in the reactor. The sealed reactor was charged to 2 MPa with oxygen and heated to 125 °C at a heating rate of 5 °C min^−1^ under stirring at 600 rpm. The cooled solution was detected by liquid chromatography–mass spectrometry (LC–MS).

### Confocal fluorescence on microdroplets

An oil-in-water emulsion was prepared by ultrasonically emulsifying 0.2 g of heptadecane in 5 ml of water to form oil microdroplets. Subsequently, Rhodamine 800 was added to the aqueous phase to a final concentration of 10 μM. The distribution of the fluorescent probe was then observed using confocal fluorescence microscopy with a wavelength of 682 nm.

### Products analysis

The products analysis can be divided into three parts according to the phase state, namely, gas, liquid and solid products.

#### Gas products

The gas products from the Parr autoclave headspace could be quantified by the state equation for ideal gas. The temperature and pressure of the system after reaction could be directly read out by the temperature indicator and electronic pressure gauge. The internal volume of the Parr autoclave was measured by filling it with water. Then gas products were collected by a 0.5-l Teflon bag. The concentrations of CO and CO_2_ were determined using a gas chromatography–flame ionization detector (GC-FID) equipped with a methanization converter, which reduces CO and CO_2_ to CH_4_ over a Ni catalyst for subsequent FID detection, and a packed column (TDX-01, 2 m × 3 mm × 2 mm) with N_2_ as the carrier gas at a column temperature of 50 °C. The standard curves of the concentrations and their peak areas were obtained by an external standard method.

#### Liquid products

The liquid products are dominated by strongly polar, saturated dicarboxylic acids (predominantly C_4_–C_8_ diacids). To enable more accurate quantification of the individual products, the carboxylic acids were derivatized into their corresponding methyl esters using TMS-CHN_2_ under mild conditions before GC analysis. First, the solvents (water and methanol) were evaporated at 80 °C. Then, 14 ml of ether and 4 ml of methanol were added. While stirring in an ice-water bath, 1.6 ml of 1.5 mol l^−1^ TMS-CHN_2_ (in *n*-hexane) was added dropwise, followed by stirring for 2 h. Subsequently, an extra 0.16 ml of 1.5 mol l^−1^ TMS-CHN_2_ (in *n*-hexane) was added and the reaction was stirred for another 3 h. Finally, the reaction was quenched with 0.14 ml of acetic acid. After centrifugation, the liquid products were analysed on a GC-FID equipped with a capillary column (SH-Stabilwax, 30 m × 0.25 mm × 0.25 μm). The temperature ramp programme was: 40 °C (hold 6 min) and ramp 10 °C min^−1^ to 260 °C (hold 20 min) to ensure that all of the liquid products could be measured. The liquid products were also identified by a gas chromatography–mass spectrometer equipped with a capillary column (SH-Rtx-1, 30 m × 0.25 mm × 0.25 μm). The temperature ramp programme was: 40 °C (hold 10 min) and ramp 10 °C min^−1^ to 270 °C (hold 10 min). The standard samples were esterified for the standard curve using the same procedure. Before esterification, a certain amount of diacid standard (dibasic acid standard for C_3_–C_16_) was dissolved in methanol to obtain 5.00 mg ml^−1^, 2.50 mg ml^−1^, 1.00 mg ml^−1^, 0.50 mg ml^−1^ and 0.10 mg ml^−1^ solution, respectively. The concentration of the product is calculated using the standard curve.

#### Solid products

After centrifugation, the insoluble solid was dried at 80 °C overnight and then weighed to determine the mass of the solid residue. The conversion was calculated by comparing the mass of the residue with the initial mass of the polymer. The molecular weight of the residual hydrocarbons was measured by HT-GPC (dissolved in trichlorobenzene, 150 °C).

The yield of the different products was calculated as

$${Y}_{{\rm{carbon}}}=\frac{\frac{c\times V}{{M}_{{\rm{DC}}}}\times n\times {M}_{{\rm{C}}}}{{m}_{\mathrm{C-PE}}}$$

in which *c* is the concentration of diacid quantified by standard curve, *V* is the volume of solution, *n* is the number of carbons in a dibasic acid, *M*_C_ is the atomic mass of C element, *M*_DC_ is molar mass of dibasic acid and *m*_C-PE_ is the carbon mass of PE.

The carbon balance (C.B.) was calculated as

$${\rm{C.B.}}=\frac{\sum \frac{c\times V}{{M}_{{\rm{DC}}}}\times n\times {M}_{{\rm{C}}}{+m}_{{\rm{CO}}}{+m}_{{{\rm{CO}}}_{2}}}{{m}_{\mathrm{c-PE}}}$$

in which *m*_CO_ and ${m}_{{{\rm{CO}}}_{2}}$ are the masses of CO and CO_2_, respectively.

### Scale-up experiments

The plastic substrates used for scale-up experiments were derived from commercially available PE plastic bags. A total of 300 g of shredded PE plastic was loaded into a 5-l semi-batch reactor together with 3 l of deionized water. The reactor was then pressurized with 2 MPa of O_2_ and heated to 125 °C under stirring at 600 rpm for 48 h. After the reaction, the system was cooled to room temperature and depressurized and the resulting liquid product was collected (Supplementary Video 4). The reaction solution was concentrated to 800 ml by heating at 80 °C, cooled to 40 °C and acidified to pH 2.5–3.0 using HCl. The solution was then extracted with ethyl acetate (EtOAc) in three steps (300 ml ×1, 200 ml ×2). The combined organic phases were concentrated by slow evaporation of the oil bath at 60 °C for further processing. The aqueous phase was reheated to 80 °C, treated with 5 g of activated carbon (Brunauer–Emmett–Teller (BET) surface area about 900 m^2^ g^−1^, purchased from Jiangsu Zhuxi Activated Carbon) under vigorous stirring for 20 min and hot-filtered. The filtrate was then concentrated by slow evaporation of the oil bath to 400 ml at 60 °C and slowly cooled at a rate of 0.5 °C min^−1^ to 0–5 °C in ice water. The resulting precipitate was collected by filtration, washed with 2 × 50 ml ice-cold water and identified as crude adipic acid. The mother liquor was further concentrated to 150 ml, held at 40 °C for 30 min and then cooled to 0–5 °C to yield crude glutaric acid on filtration and 2 × 30 ml cold ethanol (as received) washing. The remaining mother liquor was concentrated to 50 ml, mixed with 100 ml of ethanol, dissolved at 60 °C and subsequently cooled to 0–5 °C to obtain crude succinic acid by filtration. Each crude product was then subjected to recrystallization by heating to dissolve and cooling in an ice bath to obtain purified products.

### In situ Raman analysis

We conducted confocal Raman microscopy using a LabRAM HR800 system (Horiba Jobin Yvon) equipped with a 531.95-nm laser light source (frequency-doubled Nd:YAG laser 20 mW) and a charge-coupled device (CCD) detector. The system used an Ar+ 532-nm laser excitation source, a 400-μm pinhole and a 100-μm entrance slit, along with a 50× long-working-distance objective lens. Two types of diffraction grating (1,800 grooves mm^−1^ and 600 grooves mm^−1^) were used to obtain spectra at different resolutions. Before each set of measurements, the system was calibrated using a silicon wafer, with the silicon peak set to 520.7 cm^−1^.

A fused silica capillary reactor containing deionized water was mounted on the microscope stage and the stage height and orientation were adjusted to clearly visualize the capillary. The optical path was then switched for Raman spectral acquisition. Spectra were recorded over the 400–4,000-cm^−1^ range with an exposure time of 30 s and two accumulations per measurement, enabling analysis of the chemical bonding features.

### Industry upscaling framework

The upcycling process operates under mild aqueous conditions without the use of precious metal catalysts or complex solvents, indicating strong potential for industrial scale-up and subsequent techno-economic analysis (TEA). The key financial metrics are reported using a 15-year project horizon. It should be noted that this TEA represents a conceptual-level analysis based on laboratory-scale experimental data and standard engineering correlations. Key assumptions, including the 12 h residence time, product price and capital cost estimates, carry inherent uncertainties that are typical of early-stage process evaluations. The sensitivity analysis provided in Extended Data Fig. 9d addresses these critical uncertainties.

#### Reactor design and throughput optimization

The TEA uses a 12 h residence time assumption based on a continuous plug-flow reactor (PFR) configuration. This design addresses the limitations of batch reactors by enabling: (1) continuous microdroplet generation; (2) segmented thermal management; and (3) distributed oxygen supply. These features are engineered to substantially improve kinetic performance relative to non-optimized batch systems. The economic effect of varying residence times from 3 h to 48 h is detailed in the sensitivity analysis in Extended Data Fig. 9e. At larger scales, established industrial mixing and contacting strategies can be used.

*Continuous plug-flow feeding*. A plug-flow configuration with a melt/feeding module and inline emulsification stabilizes droplet formation, limits solids inventory and allows precise residence-time control, facilitating scale-up.

*High-shear and turbulent agitation*. A combined impeller strategy can be used: (1) a high-shear impeller (for example, serrated turbine) in the lower reactor zone fragments molten polymer into fine droplets; (2) an upper axial-flow impeller circulates material to the shear zone, minimizing dead zones; (3) gas entrainment along the shaft enhances O_2_ or air distribution, improves gas–liquid mixing, reduces slurry density and promotes sustained oil–water contact.

*Further interfacial intensification*. If required, Venturi or other hydrodynamic mixers can generate intense shear and turbulence to produce fine droplets and renew interfacial area. Small amounts of emulsifiers can further stabilize dispersed droplets. Such approaches are widely applied in industrial gas–liquid and solid–liquid–gas processes and are directly compatible with molten-polymer-in-water systems.

Operationally, the key controllable parameters are polymer melting/softening temperature, shear intensity, stirring rate and oxygen (or gas) management. Collectively, these measures allow replication and intensification of the microdroplet interfacial environment observed in the laboratory at larger throughput, while maintaining energy efficiency and operational safety using established engineering practices.

#### Feedstock logistics

Establishing robust preprocessing systems compatible with heterogeneous commercial plastic waste streams, including advanced spectroscopic sorting (for example, AUTOSORT FLAKE integrates near-infrared spectroscopy, high-resolution imaging, metal detection and AI-assisted classification) for polymer identification and separation, followed by cleaning. Molten or softened polymers can then be continuously delivered using heated screw feeders or extruders, enabling stable feeding, composition control and seamless integration with downstream oxidative upcycling units. The framework applies a 20% relative solvent reduction on scale-up, with reactant and auxiliary stream flow rates scaled linearly^52^.

#### Material recycling and energy recovery

Heat integration strategies were applied to reduce steam and cooling water requirements. Oxygen and water were internally recycled to minimize fresh feedstock inputs. The Calculator module in Aspen was used to ensure constant feed ratios under steady-state conditions, with water-to-PE and oxygen-to-PE mass ratios fixed at 8:1 and 3.15:1, respectively.

#### Product separation

After the reaction, the cooled gas–liquid effluent was subjected to a flash tank for separation. The gaseous fraction (containing CO) was combusted for safety; the resulting flue stream was then processed using a MEA-based CO_2_ capture unit (assumed CO_2_ capture efficiency = 50% (ref. ^53^)). This separation step was not explicitly modelled in Aspen but was treated using representative industrial data. The liquid fraction was flashed to separate water from higher-boiling products. The recovered water was recycled to the process, with a 1% purge stream to prevent impurity accumulation. The product stream was routed to a reactive distillation to convert succinic and glutaric acids to their corresponding anhydrides^54^. The anhydrides were separated from residual mixed diacids and the two anhydride products were further purified by two fractional distillations.

A full list of TEA assumptions (product yield composition, utility consumption rates, capital expenditure breakdown, operating expenditure items and key financial parameters) is provided in Supplementary Tables 6–10.

### Life-cycle assessment methods

To demonstrate our upcycling environmental benefit, a comprehensive and systematic life-cycle assessment (LCA) is used, followed four phases defined in the ISO 14040 standards^55^: (1) goal and scope definition; (2) inventory analysis; (3) impact assessment; and (4) interpretation. Choices about the study, including the intended application, the methodological framework, the system boundary, the functional unit and the approach to multifunctionality, are made in the goal and scope phase. Environmental flow for all inputs and outputs of each process are collected during the life-cycle inventory (LCI) phase, encompassing raw materials, energy streams, emissions and wastes. Both the foreground and background inventories are subsequently translated into quantified environmental impacts during the life-cycle impact assessment phase. This translation uses characterization methods grounded in scientifically established environmental mechanisms that trace cause–effect pathways through which elementary flows—emitted substances or consumed resources—contribute to specific environmental damage categories. Finally, the interpretation phase critically evaluates whether conclusions derived from the impact assessment are sufficiently substantiated and robust, examining key assumptions, performing sensitivity analyses across various scenarios and identifying limitations before formulating recommendations for decision-making.

The LCA follows the guidance of ISO 14040 standards and the International Reference Life Cycle Data System (ILCD) Handbook^56^, as detailed below. The nominal LCA (phases 2 and 3) is calculated using the Brightway 2.5 package^57^ with the ecoinvent 3.11 (ref. ^58^) database.

#### Goal and scope definition

The goal of this study is to compare the environmental impacts of a new chemical upcycling technology against established end-of-life management options for waste PE in Europe, namely, mechanical recycling (MR), incineration and landfilling, aiming to evaluate the potential sustainability benefit of chemical upcycling as an alternative pathway. This study follows an attributional LCA framework.

The system boundary is defined as a gate-to-gate waste treatment system, starting from the entry of waste PE into the treatment facility and ending at the completion of waste PE treatment and the production of valuable chemicals. Pretreatment requirements differ substantially across the three scenarios. For landfilling and incineration, only waste transport is included, as these processes accept mixed plastic waste with minimal preprocessing—a conservative assumption that does not disadvantage the baseline scenarios. For the chemical recycling process and the MR process, a full pretreatment chain comprising transport, sorting, washing, drying, shredding, grinding and pelletization is included, consistent with its feedstock purity requirements. System boundary diagrams for all scenarios are provided in Extended Data Fig. 10a.

The functional unit is defined as the treatment of 1 kg of waste PE and the corresponding reference flow is 1 kg of waste PE. As the upcycling process results in the joint production of several valuable chemical products, as well as the liquid CO_2_, the system exhibits multifunctionality. Subdivision of the process is not applicable owing to the integrated nature of the joint production system.

Therefore, multifunctionality was addressed through system expansion. For the upcycling scenario, avoided burdens were assigned for the virgin chemical products and liquid CO_2_ displaced by the upcycling system. As the recovered chemical products exhibited purities of more than 99%, a substitution factor of 1 was assumed. For the incineration and landfill scenarios, credits were assigned for recovered electricity and heat, assumed to displace grid electricity and industrial heat production, respectively. Landfill gas capture and use generate 0.006389 kWh of electricity and 0.01152 MJ of useful heat per functional unit, whereas incineration generates 1.38 kWh of electricity and 9.62 MJ of thermal energy per functional unit. A substitution factor of 1 was applied to both energy products. For the MR scenario, recycled PE was assumed to substitute virgin LDPE and HDPE according to their respective shares in the European plastics market, with LDPE and HDPE accounting for 58% and 42%, respectively^59^, which were used as proxies for the composition of the waste PE stream. To account for quality degradation and regulatory or market constraints limiting the use of mechanically recycled plastics in certain applications, substitution factors of 0.5 for LDPE and 0.65 for HDPE were applied on the environmental assessment of plastic waste recycling^60^.

#### Life-cycle inventory

Mass and energy flows for the waste PE treatment were modelled using Aspen Plus based on an optimized process flow diagram. These foreground inventories were subsequently integrated with background datasets from the ecoinvent database to quantify the LCIs for all scenarios. A detailed list of foreground flows for the upcycling technology, normalized to the functional unit, is provided in Supplementary Table 11. A conservative estimation approach was used during LCI construction to prevent favourable bias towards the upcycling scenario.

Owing to the lack of a directly applicable background dataset and the high process cooling demand, the cooling utility was modelled using two scenarios: a worst-case scenario using ecoinvent data and a standard scenario in which a wet cooling tower was sized on the basis of the estimated cooling heat duty. Inventory data for the cooling tower were adopted from the disaggregated LCA developed in ref. ^61^, in which fan and pump electricity consumption, decarbonized make-up water, blowdown wastewater and evaporative water losses were shown to account for more than 97% of total life-cycle impacts. Accordingly, only these flows were included in the present study.

The LCIs for conventional treatment options were proxied using the following ecoinvent datasets: ‘treatment of waste polyethylene, sanitary landfill {CH} | Cut-off’ and ‘treatment of waste polyethylene, municipal incineration FAE {CH} | Cut-off’, combined with ‘market for transport, freight, lorry, unspecified {RER} | Cut-off’ for logistics. The avoided products of electricity and heat recovered from incineration and landfill were modelled using ‘market group for electricity, medium voltage {RER} | Cut-off’ and ‘market group for heat, district or industrial, natural gas {RER} | Cut-off’, respectively. MR scenarios were modelled using the ecoinvent datasets ‘polyethylene production, low density, granulate {RER} | Cut-off’ and ‘polyethylene production, high density, granulate {RER} | Cut-off’ as proxies for recycled LDPE and HDPE production, respectively.

#### Life-cycle impact assessment

The LCI results were characterized into environmental impacts using the cumulative energy demand (CED)^62^ and ReCiPe 2016 methodology^63^. These impacts were first categorized into 18 midpoint indicators, including global warming, toxicity, ozone depletion and land use, and then further aggregated into three end-point categories: the damage areas of resources, human health and ecosystems quality. This multi-indicator approach ensured a holistic evaluation of the environmental trade-offs between the new upcycling process and conventional waste management options. The results, normalized to the functional unit, are presented in Supplementary Tables 12 and 13.

#### Interpretation

Contribution analyses were initially performed to identify the key parameters influencing the environmental impacts. We then conducted a scenario analysis to evaluate the effects of parameter variations on the overall environmental burdens. On the basis of these results, recommendations were formulated to inform future process optimization and industrialization pathways.

### LCA results

Extended Data Figure 10b presents the nominal LCA results for greenhouse gas (GHG) emissions and CED per kg of waste PE treated. The complete set of midpoint and end-point impact indicators is reported in Supplementary Tables 12 and 13. The comparison shows that, in the standard scenario, the proposed upcycling technology has lower GHG emissions than both pure incineration (−0.30 versus 2.11 kg CO_2_-eq) and pure landfilling (−0.30 versus 0.15 kg CO_2_-eq), turning the process from a source of emissions into a net carbon sink. Its GHG emissions are only slightly higher than those of MR (−0.30 versus −0.35 kg CO_2_-eq), showing that the proposed upcycling technology can achieve GHG emission performance comparable with MR. For CED, the upcycling technology provides clear energy savings (−17.00 MJ) compared with landfilling (0.36 MJ), although incineration (−22.54 MJ) and MR (−28.16 MJ) save more energy as a result of direct energy recovery and avoided production of new materials, respectively. In the worst-case scenario, these benefits become smaller—GHG emissions increase to 0.71 kg CO_2_-eq and the CED benefit reduces to −1.54 MJ—but the technology still outperforms incineration on GHG emissions and still saves energy overall. Landfilling has low GHG emissions but is limited by land availability and long-term environmental risks, whereas MR is limited by strict requirements on input quality and the degradation of material properties after repeated recycling.

At the end-point level under the standard scenario, the upcycling technology performs better than incineration and landfilling across the three damage categories—human health, ecosystem quality and resource scarcity—and only slightly worse than MR. At the midpoint level, the upcycling technology outperforms incineration in 8 out of 18 indicators and landfilling in 10 out of 18 indicators, whereas it falls slightly short of MR across all indicators. Although the upcycling technology does not outperform MR on any individual midpoint indicator and shows mixed results against incineration and landfilling at the midpoint level, it delivers a more favourable overall profile at the end-point level, ranking close to MR and clearly above incineration and landfilling across the three damage categories. Given the conservative credit assignment used here—and the omission of substitution effects for high-carbon diacids mixture—the actual environmental performance of the proposed technology is probably better than reported, which supports its potential as a complementary route for PE waste management.

To better understand the structure of impacts and identify opportunities for further improvement of the upcycling technology, contribution analyses were conducted under the worst-case scenario for GHG emissions and terrestrial acidification potential (TAP) (Extended Data Fig. 10c,d). The worst-case scenario was selected as the basis for this analysis because it represents the most burdensome configuration of the system, in which the underlying impact structure most clearly reveals where the largest improvement opportunities lie. GHG emissions and TAP were chosen as the representative impact categories because they reflect the two contrasting outcomes of the comparison with conventional waste treatment: the upcycling technology shows lower GHG emissions but higher TAP than incineration and landfilling. Analysing both therefore reveals the drivers behind its environmental advantages as well as its remaining limitations. The results indicate that cooling energy is the dominant contributor, accounting for approximately 34% of the total GHG emissions score and 28% of the total TAP score. The feedstock PE pellets are the second-largest contributor, accounting for approximately 25% of the total GHG emissions score and 22% of the total TAP score. Notably, oxygen feedstock contributes only about 13% to the GHG emissions score, while accounting for 21% of the TAP score, reflecting differences in characterization factors and substance-specific impact pathways across the two impact categories.

Taken together, these findings identify cooling energy as the most influential point of the system, which is also consistent with the substantial performance gap between the worst-case and standard scenarios—a gap driven mostly by differences in how the cooling utility is modelled. The favourable performance observed under the standard scenario is technically achievable at industrial scale through the use of properly sized cooling infrastructure. Apart from cooling energy, oxygen supply represents the next main opportunity for further reducing the environmental impact. Future developments in water-electrolysis-based green hydrogen technologies, in which oxygen is generated as a co-product, may substantially lower the environmental burden from oxygen supply^64^. Such technological advances could enhance the overall environmental performance of the upcycling process and highlight the importance of considering dynamic background system changes in prospective LCAs.

### Ethics statement

This study did not involve human participants or animal experiments.

## Online content

Any methods, additional references, Nature Portfolio reporting summaries, source data, extended data, supplementary information, acknowledgements, peer review information; details of author contributions and competing interests; and statements of data and code availability are available at https://doi.org/10.1038/s41586-026-10746-7.

## Supplementary information

Supplementary Information Supplementary Tables 1–13

Peer Review File

Supplementary Video 1

Supplementary Video 2

Supplementary Video 3

Supplementary Video 4

## Data Availability

The data that support the findings of this study can be found in the manuscript and its Supplementary Information or are available from the corresponding author on request.
