# Author Correction: Catalyst-free, microdroplet-mediated waste plastic conversion to diacids

**DOI:** 10.1038/s41586-026-11252-6

**Published:** 2026-10-21

**Authors:** Ruiliang Gao, Liwei Zhang, Richard J. Lewis, Hao Wang, Zhiyan Pan, Yage Zhang, Zekai Yu, Zhiqiang Liu, Xiaolin Guo, Xiangbowen Du, Wencong Liu, Minghang Li, Shipan Liang, Bing Lu, Ichiro Daigo, Shanjun Mao, Graham J. Hutchings, Yong Wang

**Affiliations:** 1 Advanced Materials and Catalysis Group, Zhejiang Key Laboratory of Low-Carbon Synthesis of Value-Added Chemicals, State Key Laboratory of Clean Energy Utilization, Institute of Catalysis, Department of Chemistry, Zhejiang University, Hangzhou, People’s Republic of China; 2 Max Planck Cardiff Centre on the Fundamentals of Heterogeneous Catalysis (FUNCAT), Cardiff Catalysis Institute, School of Chemistry, Cardiff University, Cardiff, UK; 3 Research Center for Advanced Science and Technology (RCAST), The University of Tokyo, Meguro, Japan; 4 Institute of Environmental-Chemical Engineering, College of Environment, Zhejiang University of Technology, Hangzhou, People’s Republic of China; 5 College of Materials and Chemistry, China Jiliang University, Hangzhou, People’s Republic of China

**Keywords:** Synthetic chemistry methodology, Pollution remediation

Correction to: *Nature* https://doi.org/10.1038/s41586-026-10746-7 Published online 15 July 2026

In the version of the article initially published, the Competing interests statement was incorrect and has now been amended in the HTML and PDF versions of the article.

