# Supplementary material for: Catalyst-free, microdroplet-mediated waste plastic conversion to diacids
Source: Nature. 2026 Jul 15;655(8124):917–24. doi: 10.1038/s41586-026-10746-7 (PMC13391378; doi:10.1038/s41586-026-10746-7)
Supplement: Supplementary file 1 — Supplementary Tables 1–13 [file 41586_2026_10746_MOESM1_ESM.pdf]

nature portfolio

https://doi.org/10.1038/s41586-026-10746-7

Supplementary information

Catalyst-free, microdroplet-mediated waste plastic conversion to diacids

In the format provided by the authors and unedited

Supplementary Information for

Catalyst-free, microdroplet-mediated waste plastic conversion to diacids

Ruiliang Gao (高瑞良)[1], Liwei Zhang (张立炜)[1], Richard J. Lewis[2], Hao Wang (王浩)[3], Zhiyan Pan (潘志彦)[4], Yage Zhang (张亚各)[4], Zekai Yu (俞泽楷)[1], Zhiqiang Liu (刘志强)[1], Xiaolin Guo (郭晓琳)[5], Xiangbowen Du (杜向博文)[1], Wencong Liu (刘文聪)[1], Minghang Li (李明航)[1], Shipan Liang (梁世潘)[1], Bing Lu (陆冰)[1], Ichiro Daigo (醍醐市朗)[3], Shanjun Mao (毛善俊)[1]*, Graham J. Hutchings[2]*, Yong Wang (王勇)[1]*

[1]Advanced Materials and Catalysis Group, Zhejiang Key Laboratory of Low-Carbon Synthesis of Value-Added Chemicals, State Key Laboratory of Clean Energy Utilization, Institute of Catalysis, Department of Chemistry, Zhejiang University, Hangzhou 310058, P. R. China

[2]Max Planck, Cardiff Centre on the Fundamentals of Heterogeneous Catalysis FUNCAT, Cardiff Catalysis Institute, School of Chemistry, Cardiff University, Cardiff CF24 4HQ, United Kingdom

[3]Research Center for Advanced Science and Technology (RCAST), The University of Tokyo, Japan

[4]Institute of Environmental-Chemical Engineering, College of Environment, Zhejiang University of Technology, Hangzhou 310014, P. R. China

[5]College of Materials and Chemistry, China Jiliang University, Hangzhou 310018, P. R. China

E-mail: maoshanjun@zju.edu.cn; Hutch@Cardiff.ac.uk; chemwy@zju.edu.cn.

Table of content

Table S1: Comparison between Pore-Modulated Pyrolysis and Microdroplet-Interfacial Oxidation Strategy 3

Table S2. Conversion of PE oxidation in water, various organic solvent and solvent-free conditions (2 MPa 125 ℃ 12 h) 4

Table S3. Metal leaching after reaction detected by ICP-MS 5

Table S4. Proximate analysis of commercial PE and PE standard sample in lab. 6

Table S5. Ultimate analysis of commercial PE and PE standard sample in lab. 6

Table S6. Summary of product sales revenue (60 kton) 7

Table S7. Summary of product costs (60 kton) 8

Table S8. Key assumptions about capital investment (60 kton) 9

Table S9. Key assumptions about profits (60 kton) 10

Table S10. Summary of TEA (60 kton) 10

Table S11. Life Cycle Inventory (LCI) data for the chemical recycling process (per functional unit) 11

Table S12. LCA ReCiPe midpoint and CED results (per functional unit) 13

Table S13. LCA ReCiPe endpoint results (per functional unit) 15

Table S1: Comparison between Pore-Modulated Pyrolysis and Microdroplet-Interfacial Oxidation Strategy

	Pore-Modulated Pyrolysis (*Nat. Chem. Eng.*, 2025, 2, 424–435)	This Microdroplet-Interfacial Oxidation
Reaction Type	Pyrolysis	Oxidation
Conditions	420–630 °C, Atmospheric/Inert pressure	125 °C, 2 MPa O_2
Primary Products	C_8–C_{18} hydrocarbon mixture	C_4-C_8 dicarboxylic acids
Product Selectivity	Up to 80.8% for C_8–C_{18} hydrocarbons	Up to 69.6% for C_4-C_8 diacids
Product Value	Fuels / Energy	High-value Platform Chemicals for polymers, food, and pharmaceuticals

Table S2. Conversion of PE oxidation in water, various organic solvent and solvent-free conditions (2 MPa 125 °C 12 h)

Solvent	PE Conversion (%)
Water	100.0
Ethanol	12.4
Methanol	3.3
Dichlorobenzene	8.3
DMSO	11.6
Acetonitrile	4.3
THF	6.8
90% Methanol+10% Water	3.4
70% Methanol+30% Water	11.7
30% Methanol+70% Water	34.6
10% Methanol+90% Water	62.9
No-solvent	10.2

Table S3. Metal leaching after reaction detected by ICP-MS

Metal	Concentration (ppb)	Mass (mg/5 mL)
Ni	2.39	1.19E-05
Mo	0.80	4.00E-06
Fe	0.77	3.86E-06
Cr	0.72	3.60E-06
W	0.16	8.23E-07
Co	0.02	9.68E-08
Pd	0.00	7.14E-10
Pt	0.00	3.57E-10
Ru	0.00	3.57E-10
Re	0.00	0.00E+00

* The concentrations of most metals were below 1 ppb (all under 5 ppb), indicating that no detectable metal leaching occurred from the Hastelloy reactor.

Table S4. Proximate analysis of commercial PE and PE standard sample in lab.

	**M** $_{ad\%}$	**A** $_{ad\%}$	**V** $_{ad\%}$	**FC** $_{ad\%}$
LDPE bag	0.03	0.22	99.71	0.04
Gloves	0.31	1.07	98.56	0.06
LDPE package	0.07	0.32	99.58	0.03
HDPE lid	0.15	3.32	96.14	0.39
PE in lab	0.07	0.12	99.74	0.07

M $_{ad\%}$: Moisture, air-dried; **A** $_{ad\%}$: Ash, air-dried; **V** $_{ad\%}$: Volatile matter, air-dried; **FC** $_{ad\%}$: Fixed carbon, air-dried

Industrial analysis further shows that the volatile content of all samples exceeds 96%, in good agreement with the TGA results for PE. Among the samples, the lid materials show the highest ash content (3.32%), suggesting the presence of more inorganic fillers (such as calcium carbonate or titanium dioxide), which is reasonable given its need for higher mechanical rigidity. In contrast, the PE standard sample has the lowest ash content (0.12%), indicating the highest purity.

Table S5. Ultimate analysis of commercial PE and PE standard sample in lab.

	**C** $_{ad\%}$	**H** $_{ad\%}$	**N** $_{ad\%}$	**St** $_{ad\%}$	**O** $_{ad\%}$
LDPE bag	88.02	9.46	0.03	0.94	1.30
Gloves	86.94	9.71	0.12	0.23	1.62
LDPE package	86.31	10.12	0.01	0.60	2.57
HDPE lid	86.02	9.30	\	0.20	1.01
PE in lab	86.89	10.46	\	1.17	1.29

C $_{ad\%}$: Carbon, air-dried; **H** $_{ad\%}$: Hydrogen, air-dried; **N** $_{ad\%}$: Nitrogen, air-dried; **St** $_{ad\%}$: Sulfur, total, air-dried; **O** $_{ad\%}$: Oxygen, air-dried

Elemental analysis confirms that carbon and hydrogen are the main elements in all samples, although some differences are observed. The plastic sealing bags contain a relatively high oxygen content (2.57%), likely due to oxygen-containing additives used to improve flexibility. Disposable gloves contain trace nitrogen (0.12%), possibly originating from antistatic agents. The PE standard sample shows a relatively higher sulfur content (1.17%), which may result from residual catalysts or antioxidants.

Table S6. Summary of product sales revenue (60 kton)

Product	Output (t)	Market price ($/t)	Revenue (million $)
Succinic anhydride (>99%)	43426.1	2953.2	12824.4
Glutaric anhydride (>99%)	12144.2	3482.5	4229.2
Mixed diacids (C_{6-9})	13128.1	696.5	914.4
**Total**			**17968.0**

* All TEA were calculated in RMB and converted to US dollars at the exchange rate of January 29, 2024.

Table S7. Summary of product costs (60 kton)

**Operating time h/yr**	8,000	**Capacity t/yr**		60,000
	**item**	**quantity per year (t)**	**cost per unit (USD)**	**Calculated value (10^4 USD)**
	**Raw materials**			
**1**	PE	60000	835.8/t	5014.8
**2**	O_2	63000	57.4/t	361.6
**3**	H_2O	7559.7	0.42/t	0.3
**4**	MEA solvent	47.5	1500/t	7.1
	**Utilities**			
**1**	Electricity	$1663.7*10^4$	0.10	173.8
**2**	Water	$270.1*10^4$	0.077	18.8
**3**	Low-pressure steam	$16.2*10^4$	30.65	495.9
**4**	Medium-pressure steam	$11.6*10^4$	30.65	355.0
**Total**				6427.4

* Low-pressure steam: 125 °C, Medium-pressure steam: 250 °C.

*Note on CO_2 Capture Unit: The MEA absorption system is designed for a 50% capture efficiency. Solvent regeneration energy is set at 4.0 GJ/t CO_2, sourced from low-pressure steam and electricity. MEA loss is accounted for at a rate of 1.0 kg/t CO_2.

Table S8. Key assumptions about capital investment (60 kton)

	Cost(10^4 \$)	Note
**Fixed capital investments**	**9056.1**	
(1) Direct cost	*7111.0*	
Costs of major equipment	2788.6	
reactor	1316.4	
tower	438.8	
sorting and feeding	11.7	
tank& pump& pipe	109.0	
heat exchanger	146.3	
compressor	341.3	
flash evaporator	37.6	
combustion furnace	20.9	
gas separator (PSA)	292.5	
mixer	8.4	
MEA unit	65.8	
Other direct costs		(specified as % of equipment cost)
Installation	557.7	20%
Piping	557.7	20%
Instrumentation and control	557.7	20%
Building & structure	836.6	30%
Yard improvement	278.9	10%
Service facilities	1394.3	50%
Land	139.4	5%
(2) Indirect cost	*1945.1*	
Engineering & supervision	648.4	15%
Legal expenses	86.4	2%
Construction expenses	648.4	15%
Contractor's fee	129.7	3%
Contingency	432.2	10%
**Working capital**	**1358.4**	15% of fixed capital investment
**Others**	**3963.6**	
Depreciation period	10 yr	
Annual depreciation rate	0.1	
Depreciation charge	264.9	
Annual interest rate	3%	
Operating labor	642.1	10% of total product cost
Operating supervision	32.1	5% of operating labor
Utilities	963.2	15% of total product cost
Maintenance and repairs	642.1	10% of fixed capital investment
Operating supplies	192.6	30% of maintenance and repairs
Laboratory charges	6.4	20% of operating labor
Royalties	256.9	4% of TPC without depreciation
Plant overhead costs	321.1	5% of total product cost

General expenses	642.1	10% of total product cost

Table S9. Key assumptions about profits (60 kton)

	Cost(10^4 \$)	Note
Variable cost	6427.4	
Salaries	642.7	10% of variable cost
Depreciation	264.9	
Maintenance and repairs	271.7	3% of fixed capital investments
Insurance	63.4	0.7% of fixed capital investments
Total cost	7670.1	
Annual profit	10297.9	
Business income tax	3089.4	30% of annual profit
Net profit	7208.5	
amortization expense	315.9	

Table S10. Summary of TEA (60 kton)

Total capital investment, million $	143.8
Fixed capital investment, million $	90.6
Raw material and power costs, million $	64.3
Total sales volume, million $	179.7
Breakeven point, kton	9.0
Net profits, million $	72.1
Investment profit rate	44%
Static payback period, yr	3.3

* All TEA were calculated in RMB and converted to US dollars at the exchange rate of January 29, 2024.

Table S11. Life Cycle Inventory (LCI) data for the chemical recycling process (per functional unit)

Group	Inventory	Flow	Unit	Comment
**Inputs**	Market for chemical factory, organics {GLO} | Cut-off	4.71E-10	unit	Based on industry average for chemical infrastructure [1]
	Market for heat, from steam, in chemical industry {RER} | Cut-off	3.94	MJ	Net steam demand after heat integration
	Market for cooling energy {GLO} | Cut-off [a]	14.78	MJ	Cooling demand after heat integration
	Market for oxygen, liquid {RER} | Cut-off	0.83	kg	Total oxygen demand (stoichiometric + combustion)
	Market for polyethylene, pellets, recycled {RER} | Cut-off	0.76	kg	Proxy for pretreated waste PE; 76.34% conversion to PE pellets assumed.
	Market for water, deionised {Europe without Switzerland} | Cut-off	0.16	kg	Combined process water and hydration requirements
	Market group for electricity, medium voltage {RER} | Cut-off	0.28	kWh	Electricity for pumping and compression processes
	Carbon dioxide production, liquid {RER} | Cut-off [b]	0.30	kg	Mass and energy flow for MEA-based CO_2 capture (including regeneration)
**Emission**	Carbon dioxide, fossil, Air, urban air close to ground	0.30	kg	Uncaptured CO_2 emissions from liquid CO_2 production, overriding ecoinvent's default zero-emission input assumption

	Water, unspecified	0.14	m^3	Water from distillation column
Output	Succinic anhydride (99.0%) [c]	0.56	kg	Product yield based on process simulation results
	Glutaric anhydride (99.2%) [d]	0.16	kg	Product yield based on process simulation results
	High-carbon diacids mixture [e]	0.17	kg	Product yield based on process simulation results
	Carbon dioxide production, liquid {RER} | Cut-off	0.30	kg	Product recovery based on 50% separation/purification efficiency [1]

[a] This background dataset is modelled as a standard alternative wet cooling tower scenario using proxied ecoinvent flows: 0.0045 m^3 Water, evaporated to air {GLO} | Cut-off, 0.062 kWh market group for electricity, low voltage {RER} | Cut-off, 6.56 kg market for water, decarbonised {CH} | Cut-off, and 0.0020 m^3 market for wastewater, average {Europe without Switzerland} | Cut-off.

[b] This background dataset represents the MEA-based CO_2 capture technology.

[c] The environmental burden of "succinic acid production {GLO} | Cut-off" is avoided via system expansion. The hydration of anhydride to diacid is accounted for by adding the stoichiometric equivalent of deionised water.

[d] The environmental burden of "succinic acid production {GLO} | Cut-off" is avoided similarly to Footnote 2. This substitution is justified by the higher market value of the product compared to succinic acid.

[e] The mixture comprises adipic acid, pimelic acid, suberic acid, and azelaic acid, with a total impurity level below 0.4%. No substitution is applied to this output to ensure a conservative estimation.

Table S12. LCA ReCiPe midpoint and CED results (per functional unit)

Impact Category	Indicator	Unit	Upcycling (worst)	Upcycling (standard)	Incineration	Landfill	MR
Acidification: terrestrial	Terrestrial acidification potential (TAP)	kg SO_2-Eq	2.13E-03	4.57E-04	-1.62E-03	5.80E-05	-7.38E-04
Climate change	Greenhouse gas emissions (GHGs)	kg CO_2-Eq	7.10E-01	-2.99E-01	2.11E+00	1.45E-01	-3.51E-01
Ecotoxicity: freshwater	Freshwater ecotoxicity potential (FETP)	kg 1,4-DCB-Eq	1.29E-01	6.36E-02	2.00E-01	1.54E-01	3.03E-02
Ecotoxicity: marine	Marine ecotoxicity potential (METP)	kg 1,4-DCB-Eq	1.67E-01	8.34E-02	2.90E-01	2.17E-01	3.89E-02
Ecotoxicity: terrestrial	Terrestrial ecotoxicity potential (TETP)	kg 1,4-DCB-Eq	3.28E+00	1.61E+00	8.75E+00	2.63E-01	-1.59E-01
Energy resources: non-renewable, fossil	Fossil fuel potential (FFP)	kg oil-Eq	-2.51E-01	-5.62E-01	-3.13E-01	8.19E-03	-6.28E-01
Eutrophication: freshwater	Freshwater eutrophication potential (FEP)	kg P-Eq	5.60E-04	3.84E-04	-4.24E-04	-3.57E-07	4.34E-05
Eutrophication: marine	Marine eutrophication potential (MEP)	kg N-Eq	1.66E-04	1.36E-04	-3.86E-05	2.02E-04	1.05E-04
Human toxicity: carcinogenic	Human toxicity potential (HTPc)	kg 1,4-DCB-Eq	1.36E-01	-4.03E-02	-5.66E-02	4.94E-03	-1.00E-01
Human toxicity: non-carcinogenic	Human toxicity	kg 1,4-DCB-Eq	2.32E+00	1.12E+00	1.14E+00	3.28E+00	4.89E-01

	potential (HTPnc)						
Ionising radiation	Ionising radiation potential (IRP)	kBq Co-60-Eq	3.69E-01	3.52E-01	-2.83E-01	-8.00E-04	3.45E-02
Land use	Agricultural land occupation (LOP)	m^2*a crop-Eq	2.26E-02	1.67E-02	-1.31E-02	3.37E-03	-6.09E-04
Material resources: metals/minerals	Surplus ore potential (SOP)	kg Cu-Eq	4.62E-02	2.66E-02	-7.23E-03	6.43E-04	-2.89E-03
Ozone depletion	Ozone depletion potential (ODPinfinite)	kg CFC-11-Eq	7.08E-07	3.11E-07	-1.65E-07	5.53E-09	1.43E-07
Particulate matter formation	Particulate matter formation potential (PMFP)	kg PM2.5-Eq	5.68E-04	-2.53E-04	-6.61E-04	2.91E-05	-2.83E-04
Photochemical oxidant formation: human health	Photochemical oxidant formation potential: humans (HOFP)	kg NO_x-Eq	1.44E-04	-1.10E-03	-7.78E-04	1.29E-04	-1.48E-03
Photochemical oxidant formation: terrestrial ecosystems	Photochemical oxidant formation potential: ecosystems (EOFP)	kg NO_x-Eq	-1.77E-04	-1.54E-03	-8.50E-04	1.35E-04	-1.91E-03
Water use	Water consumption potential (WCP)	m^3	3.27E-02	2.66E-02	-5.29E-03	2.49E-04	-3.01E-03
Total: CED	Energy content (HHV)	MJ-Eq	-1.54E+00	-1.70E+01	-2.25E+01	3.60E-01	-2.82E+01

Table S13. LCA ReCiPe endpoint results (per functional unit)

Impact Category	Indicator	Unit	Upcycling (worst)	Upcycling (standard)	Incineration	Landfill	MR
Total: ecosystem quality	Ecosystem quality	species.yr	3.58E-09	-1.02E-10	5.25E-09	6.04E-10	-1.38E-09
Total: human health	Human health	DALYs	2.07E-06	-2.52E-07	1.60E-06	9.18E-07	-7.32E-07
Total: natural resources	Natural resources	USD 2013	-1.02E-01	-2.08E-01	-9.48E-02	3.79E-03	-2.65E-01

References

1 Althaus, H.-J. *et al. Life Cycle Inventories of Chemicals*. ecoinvent report No. 8, v2.0 (EMPA Dübendorf, Swiss Centre for Life Cycle Inventories, 2007).
