# Supplementary material for: Catalyst-free, microdroplet-mediated waste plastic conversion to diacids
Source: Nature. 2026 Jul 15;655(8124):917–24. doi: 10.1038/s41586-026-10746-7 (PMC13391378; doi:10.1038/s41586-026-10746-7)
Supplement: Supplementary file 2 — Peer Review File [file 41586_2026_10746_MOESM2_ESM.pdf]

nature portfolio

Peer Review File

Catalyst-free, microdroplet-mediated waste plastic conversion to diacids

Corresponding Author: Professor Graham Hutchings

Any redactions in this file are there to maintain patient confidentiality, the confidentiality of unpublished data, or to remove third-party material.

This file contains all reviewer reports in order by version, followed by all author rebuttals in order by version.

Attachments originally included by the reviewers as part of their assessment can be found at the end of this file.

Version 0:

Reviewer comments:

Referee #1

(Remarks to the Author)
This is a highly innovative paper on waste plastics valorisation. The author's approach is interesting and relevant as it avoids the use of catalyst and operates under milder conditions than those of conventional waste plastics conversion processes. The paper is in general well written, and the results and reaction mechanism discussion appear to be sound. Thus, data analysis and interpretation are effective and consistent. There are, however, some points that should be addressed and clarified. The next items have the objective of clarifying some aspects of the manuscript and of improving its scientific quality.

The authors should pay attention to some highly selective non-catalytic processes for waste plastics conversion into valuable chemicals. This is the case of plastics thermal cracking at high temperatures and with short residence times. The authors should note that under these conditions very high yields of light olefins (ethylene, propylene or butenes) were reported. Obviously, the conditions of this process are severe, however, it is important to remark that there are reasonable and selective alternatives for non-catalytic conversion of waste polymeric materials.
The interest of microdroplets reactor technology for plastics and other polymeric materials valorisations should be justified in the introduction.
A basic characterization of the polymeric materials (proximate and ultimate analyses, ash composition…) used in this paper should be included in the supplementary material.
It seems that there is a mistake concerning gas product analysis, the authors included the following sentence: "The concentrations of CO and CO2 were determined by GC-FID equipped with a converter and a packed column (TDX-01, 2 m*3 mm*2 mm)". However, the FID detector cannot analyse CO and CO2.
Why was the liquid product esterified? What is the impact of this process in oil composition? Is not possible to directly analyse raw oil? Please clarify.
This sentence included in the experimental section is unclear: "Solid products. After centrifugation, the insoluble solid was dried at 80 °C overnight, and then weighed and compared with the mass of the catalysts loaded before the reaction". The authors stated that no catalyst was used in the process.
The molecular weight of the PE used is very low, it is orders of magnitude lower than that typically used in commercial polymers. What is the impact of this fact in process performance (selection of process conditions, plastics conversion, reaction rate or product distribution)?
A critical aspect in this study is the selection of process conditions. The study was carried out at low temperature and high oxygen pressure, however, the selection of these conditions was not commented or justified.
Does Figure 1 b correspond to a dicarboxylic acids yield of 69.6%? It seems that in this photo the plastic conversion is rather low, it is possible to observed a solid plastic.
The authors evaluated the performance of microdroplet technology for the valorisation of different types of real polymers (polyolefins, PS or even waste tires). The composition of these materials is very different, and a completely different reactivity and reaction mechanism can be expected for each feedstock. In fact, the conditions used with these polymeric

materials were different. The authors should discuss the role played by the polymeric material nature on the selection of suitable process conditions and process performance.
The technology readiness level of this study is low, the study was carried out at lab scale, with some experiments performed in a batch unit operating with 300 g of sample. Considering this fact, it is probably too optimistic the incorporation of a techno-economic analysis of the process.
The real challenges of the plastics valorisation route should be discussed. One of the main concerns is the slow reaction rate of the process, thus, reaction time extremely high (several hours). It is to note that other thermochemical processes for waste plastics valorisation have reaction times from few minutes to some seconds. Moreover, the process was studied in batch regime, the scale up of the process usually requires continuous operation. Is it possible to operate this process in continuous regime?
Another relevant question is the necessity of feeding the polymeric material as a fine powder. This is a major problem, on the one hand, it could be challenging the grinding of some types of waste polymers. On the other hand, it is expensive and requires a lot of energy (extremely high for waste tyres).

Referee #2

(Remarks to the Author)
Review of "Catalyst-Free Valorization of Waste Plastics via Microdroplet-Interfacial Radical Scission"
The authors present a catalyst-free strategy to upcycle plastics (primarily polyolefins) into short-chain diacids (C4–C8) using only water and O2 under mild conditions (125 °C, 2 MPa O2). This approach leverages microdroplet chemistry, exploiting the long-chain structure and hydrophobic nature of molten plastic wastes to form critical water-oil interfacial microdroplets that spontaneously generate •OH radicals, thereby facilitating oxidative polymer cleavage. Mechanistic studies employing isotopically labelled water, combined with comprehensive characterization techniques, confirm the key role of H_2O in mediating the oxidative degradation pathway. The strategy is further extended to various polyolefin feedstocks (both virgin and post-consumer) and rubber tires, and is shown to be compatible with natural water sources such as tap water and seawater. Moreover, the process was successfully scaled up to a 300 g batch to demonstrate its scalability. Overall, this work presents a promising route for converting plastic waste into value-added oxygenates under remarkably mild conditions.
While the manuscript is well written and demonstrates clear novelty and rigor, with results supported by convincing evidence, several major points should be addressed before the work can be considered for publication in Nature.
A. A major concern lies in the CO2 emissions associated with the process. According to the authors' results, approximately 25% COx (predominantly CO2) is generated, with values rising to 30–45% for certain polymers (e.g., PP and PBD). This raises significant questions regarding the environmental footprint of the approach. The reported range corresponds to roughly 0.78–1.35 kg CO2-eq per kg of polymer, potentially offsetting the sustainability benefits of the upcycling process. Moreover, the extended reaction durations (12–24 h on a small scale and >48 h on a larger scale) and the use of excess water likely contribute additional indirect emissions from energy consumption and product separation. A comprehensive life cycle analysis would therefore be essential to evaluate the overall sustainability and practical viability of the process.
B. Another concern pertains to the process feasibility for mixed waste upcycling. Although the authors demonstrate extension to several polymers (PP, PBD, PS), the reaction temperature appears to depend strongly on the polymer melting point. Operating at higher temperatures, however, risks product overoxidation and increased CO2 formation. Real-world plastic waste streams are highly heterogeneous and typically contain polymers such as PET, PVC, and PU in addition to polyolefins. The authors should provide evidence or discussion demonstrating the robustness of the process toward such mixed and additive-rich feedstocks to support claims of practical applicability.
C. Lastly, although the manuscript shows successful deconstruction of certain consumer-grade plastics, further clarification and data are needed regarding the tolerance of this catalyst-free system to additives, fillers, and other impurities. Additional discussion or experiments (as noted in the specific comments) would help to substantiate the claimed generality of the process.
D. Diacids are platform molecules. However, their production rate is about two orders of magnitude lower than that of plastics. How will the products from the conversion of most plastics be absorbed in the market?
E. Most plastics are hydrophobic. How will the scale-up in hydrophobic water happen? How will mixing/interfacial contact occur at a large scale?
In summary, this manuscript introduces an innovative and conceptually appealing strategy for plastic upcycling via interfacial microdroplet oxidation. Despite the aforementioned points, the work holds promise for publication in a high-impact journal. However, addressing these concerns (which will likely require additional experiments and analyses) will be critical for evaluating the study's broader impact and relevance to a general audience and, consequently, for determining its suitability for publication in Nature.

Specific comments are outlined below:

1. Can the process be conducted using air instead of pure O2? This would greatly enhance the practicality and scalability of the method compared to relying on high-purity oxygen.
2. In Table S1, the authors show that the absence of solvent or the use of organic solvents results in low PE conversion. However, the entry with 90% water + 10% methanol also shows almost no conversion. Can the authors explain this observation? Is this due to methanol acting as a radical scavenger? While this is a possible explanation, the tert-butyl alcohol experiments indicate that radical quenching only occurs at higher alcohol concentrations (~30 vol%). Further clarification on this discrepancy is needed. Can the authors clarify this discrepancy?
3. Is it possible to tune the carbon number of the diacid products by adjusting reaction parameters? For example, could the process be directed toward forming higher-value diacids such as sebacic acid (C10), adipic acid (C6), or suberic acid (C8),

instead of succinic acid?
4. Since radical chemistry is unselective, what controls the selectivity to the small diacids and mainly to C4?
5. The authors investigated PE degradation under varied conditions such as temperature, O_2 pressure, and water volume. However, throughout all experiments, the stirring rate was held constant at 600 rpm. Can the authors comment on whether altering the stirring rate might influence microdroplet formation and, consequently, affect reaction rates or product selectivity? Investigating this parameter could be valuable, as it may reveal more optimal operating conditions.
6. As the authors rightly mention, additives pose a major challenge in the catalytic conversion of plastic waste. Although the authors demonstrate the robustness of their process in the presence of certain metal salts, a key class of additives present in most consumer-grade plastics are antioxidants (both primary and secondary). These additives inhibit oxidation either by neutralizing free radicals or decomposing peroxides, thereby preventing degradation. Similarly, hindered amine light stabilizers act as radical scavengers to suppress photooxidation. Recent studies (Nat. Chem. Eng. 2025, 2, 220–228; J. Am. Chem. Soc. 2025, 147, 11227–11238) have shown that such additives can significantly influence catalytic plastic conversion. Although the authors demonstrate applicability to consumer-grade PE, the type and concentration of additives vary widely among products. Despite the process being non-catalytic, it depends on radical chemistry, making it especially important to understand how these additives affect reaction efficiency and product selectivity. Therefore, I recommend that the authors perform additional experiments to evaluate the influence of these additives on process performance and product outcomes.
7. Many consumer-grade plastics also contain inorganic additives (fillers) such as TiO_2, $CaCO_3$, and ZnO, which can influence process performance (https://doi.org/10.26434/chemrxiv-2025-0nfrn). Do the authors anticipate that these fillers could affect their oxidative upcycling process?
8. The authors report that benzoquinone quenches superoxide radicals (•O_2^-), thereby suppressing PE conversion (Fig. S12). A related concern arises for rubber feedstocks, which commonly contain N-(1,3-dimethylbutyl)-N′-phenyl-p-phenylenediamine (6PPD), a widely used antioxidant and antiozonant. Upon oxidation, 6PPD forms 6PPD-quinone, a compound known for its aquatic toxicity (Science 2021, 371, 185–189; Nat. Chem. Eng. 2024, 1, 597–607). Can the authors comment on whether 6PPD or its oxidation product, 6PPD-quinone, could interfere with or influence their oxidative upcycling process?
9. Real-world plastic waste streams are highly heterogeneous and typically contain polymers such as PET, PVC, PU, and others in addition to polyolefins. These materials are known to influence catalytic processes by generating undesirable byproducts (e.g., HCl from PVC decomposition; J. Am. Chem. Soc. 2025, 147, 11227–11238; Nat. Chem. Eng. 2024, 1, 376–384). Similarly, hydrolysable polymers such as PET and nylon can yield acids, amines, or alcohols that may interfere with the reaction. Although the present process is non-catalytic, such byproducts could still impact reaction pathways or product selectivity. Therefore, I strongly recommend that the authors perform experiments using heterogeneous mixed waste streams to evaluate the robustness and general applicability of their process.
10. Likewise, packaging waste often consists of multilayer films made from coextruded polymers. Typical compositions include layers of PE, PET, EVOH, EVA, and nylon. It would be valuable for the authors to demonstrate the applicability of their process to such multilayer packaging waste, given its prevalence and complexity in real-world plastic streams.
11. An added challenge with mixed plastic waste is that different polymers have varying melting temperatures. Identifying a single optimal reaction temperature suitable for the entire mixture may be difficult, as conditions that work for one polymer may cause overoxidation of others, potentially leading to higher CO_x formation. While the authors report that “further oxidation of short-chain diacids was minimal, as confirmed by control experiments using commercial diacids (Fig. S24),” this behavior is clearly temperature-dependent. When processing mixed wastes, higher temperatures may be required to accommodate polymers with higher melting points, which could increase CO_2 generation. Furthermore, the authors should discuss how individual carboxylic acids are separated from the complex product mixture, as mixed feedstocks may yield a wide range of products from different polymers.
12. The authors state that “real tires processed at 180 °C produced 24.4% inorganic solids and 57.9% liquid products”. The composition of these liquid products should be clearly specified. Based on the statement, it is unclear fraction comprises diacids versus monoacids or other species?
13. The authors state that “scalability was confirmed in a 5 L reactor (300 g PE, 3 L H_2O, 2 MPa O_2) achieving 89% conversion with saturated diacid yields over 52% after 48 h.” However, the reaction time is nearly four times longer than in the small-scale reactor. Can the authors explain the cause of this slower reactivity?
14. Fig. S20 – FTIR Analysis: The post-reaction polymer spectrum shows additional features between 1000–1500 cm^{-1} besides the carbonyl peak. Please specify clearly which functional groups these correspond to. Furthermore, it may be useful to supplement FTIR results with high-temperature ^1H-NMR to confirm bulk oxidation of PE and quantify different functional groups.
15. Given that the authors show that •OH radicals are generated even under N_2, some oxidation of unconverted PE would be expected. Do the authors observe any hydroxyl, carbonyl, or other oxygen-containing functionalities on the residual solids?
16. Figure 2D : Including EPR spectra for the PE + H_2O + O_2 system in Fig. 2D would help compare the relative amounts of •OH radicals formed under different conditions.
17. The authors must provide details on the calibration standards used and the molecular weight calibration range for the HT-GPC in the methods section. If the system was not properly calibrated, or if the product MW falls outside this range, the reported MW values may be inaccurate.
18. The authors state, “High-MW PE waste including gloves, LDPE bags, HDPE caps…”. However, these materials cannot be classified as high-MW PE without reporting their molecular weights. I suggest referring to them as consumer-grade PE instead.
19. Introduction, Lines 54–59: The authors state, “However, accumulation of miscellaneous additives and contaminants inevitably lead to catalyst deactivation,….” Please provide supporting citations for this claim.
20. Techno-Economic Analysis: It is unclear how the assumed processing capacity of 200 kt/year was determined for the TEA. This value appears overambitious relative to current industry metrics. Presently, pilot- and commercial-scale plastic depolymerization plants typically operate in the 3 –100 kt/year range, with most reports citing capacities below 60 kt $year^{-1}$

(Energy Environ. Sci. 2023, 16, 3638–3653). Moreover, the achievable scale will depend strongly on the waste-processing capacity of regional materials recovery facilities. The authors are therefore encouraged to adopt more realistic and modest assumptions for the TEA to ensure a credible and representative techno-economic evaluation. Detailed explanations must be provided to justify the assumptions underlying the TEA model. A sensitivity analysis should be performed to identify the key cost drivers and parameters influencing process economics.

Referee #3

(Remarks to the Author)
I co-reviewed this manuscript with one of the reviewers who provided the listed reports.

Version 1:

Reviewer comments:

Referee #1

(Remarks to the Author)
I have carefully reviewed the revised version of the manuscript, and I am pleased to report that the authors have addressed all previously raised concerns in a thorough and convincing manner. The revisions significantly enhance the clarity, methodological rigor, and overall contribution of the paper.
The authors have improved the exposition and provided clearer justification for their key assumptions and conclusions. The manuscript is now well-structured, readable, and aligned with the standards expected for publication in a top-tier journal.
In light of these substantial improvements, I recommend acceptance of the manuscript in its current form.

Referee #2

(Remarks to the Author)
Firstly, we would like to thank the authors for carefully considering our previous comments and undertaking a substantial revision of the manuscript. We appreciate the effort invested in this process. After reviewing the revised manuscript and the response-to-reviewers document, we believe that the authors have addressed most of our previous concerns. However, several issues still require clarification and, in some cases, additional justification. In particular, major concerns remain regarding the assumptions and results presented in the technoeconomic analysis and lifecycle assessment, as well as certain experimental results that require further clarification and/or additional analysis. Once these points are adequately addressed, we would be willing to recommend the manuscript for publication.
Additionally, while reviewing the revised manuscript, we came across a communications-type paper (Published: 10 February 2026) that conceptually uses a similar framework as the present study: Y. Chang, W. Gao, S. Wang, et al., “Aqua-Oxidation of Polyethylene Into Carboxylic Acids Under Mild Conditions: A Catalyst-Free Upcycling Strategy for Nonpolar Plastics,” Angew. Chem. Int. Ed., (2026): e23067. We were surprised to see that this paper also originates from the same university as the authors of the present study, although there is no overlap in authorship. Despite this conceptual overlap, we believe that the current manuscript is more rigorous, provides deeper fundamental insights into the process, and demonstrates broader applicability. However, I leave it to the discretion of the Editors to determine whether the publication of the other manuscript affects the present submission.

Specific comments are outlined below:
• Figs. S17, S18 and S20: Reaction times and corresponding polymer conversions are not clearly reported in these figures. This information is essential for interpreting the COx yields. For example, at elevated temperatures (e.g., 150 °C), are the higher COx yields simply a consequence of operating beyond full conversion, thereby leading to over-oxidation of primary products? Explicit reporting of conversion and reaction times is necessary to clarify this point.
• Results, Lines 255-257 & Comment 15 (Mechanistic Clarification): The authors state that “Initially, interfacial •OH abstracts hydrogen atoms from the PE chains, generating alkyl radicals. Alkyl radicals are then captured by •OH to produce alcohol intermediates (Fig. S27). Further •OH attack forms alkoxyl radicals that undergo β-scission/HAT, producing aldehyde/ketone intermediates.” Given the central importance attributed to •OH radicals in Fig. 3E (right panel), one would expect that even under N_2 (in the absence of O2), some degree of hydroxyl or carbonyl functionalization should be detectable in the recovered polymer. However, FTIR data (Fig. R11) do not appear to show such features. This discrepancy requires clarification. It is possible that FTIR, being surface-sensitive, does not adequately capture bulk modifications. If so, complementary techniques such as NMR may provide more definitive insight.
• The authors state “ Since the oxidation is exothermic, the temperature can be set slightly below the polymer’s melting point to limit over-oxidation. Polypropylene (PP) and polystyrene (PS) were selectively converted to acetic acid and benzoic acid at 125 °C and 220 °C, respectively, driven by the reactivity of tertiary sp3 carbons (Fig. S32).” While this explanation may be plausible, the mechanistic picture remains unclear. The proposed mechanism relies on microdroplet formation, which presumably requires the polymer to be in a molten state. However, polypropylene typically melts at ~160–170 °C, yet significant conversion is reported at 125 °C. If the polymer is not molten under these conditions, it is difficult to rationalize how the proposed microdroplet-mediated mechanism operates. This represents a mechanistic inconsistency that must be addressed. Additionally, providing DSC data for all tested polymers would be highly valuable, both to justify the chosen reaction temperatures and to clarify phase behavior under operating conditions.
• Technoeconomic Analysis: Although the authors have expanded the discussion of the technoeconomic analysis, the underlying assumptions are still not described with sufficient depth or transparency. Several key parameters remain vague,

and critical inputs are either insufficiently justified or not explicitly stated. Greater clarity and a more rigorous presentation of assumptions are necessary to enable proper evaluation of the robustness and credibility of the analysis.
o Feedstock sourcing: It is unclear how and from where the polyethylene feedstock is sourced. Because the proposed process relies on a relatively pure polyethylene stream rather than mixed plastic waste, collection, sorting, and transportation costs should be explicitly included. Numerous technoeconomic studies of plastic chemical recycling have shown that these upstream logistics are among the largest cost drivers in the overall process, including work by Beckham et al. (Energy Environ. Sci., 2023, 16, 3638–3653) and Hernández et al. (ACS Sustainable Chemistry & Engineering, 2023, 11, 7170–7181). These costs must either be incorporated into the model or clearly justified if excluded.
o MEA-based CO_2 capture: The authors state that residual CO2 is captured using an MEA-based absorption unit; however, no explicit capital or operating costs are presented. It is unclear whether this unit is included within the “Gas separator” category. MEA-based CO2 capture typically requires approximately 3.5–5.0 GJ per tonne of CO2 for solvent regeneration, significantly affecting both CAPEX and OPEX. It is unclear whether these energy demands and associated costs have been accounted for.
o Retention time: In the industrial upscaling framework, a 12 h residence time is assumed. However, the authors’ own scale-up experiments required 48 h to achieve performance comparable to laboratory-scale results. This discrepancy is substantial and must be justified quantitatively. The choice of a 12 h residence time appears optimistic and may materially affect projected economics.
o Water recycle assumption: The authors state that recovered water is recycled with a 1% purge stream to prevent impurity accumulation. The basis for selecting 1% is not explained. This assumption should be justified, and its impact on process performance and economics should be analysed.
o Table S8 – “Gas separator” unit: It is unclear which specific operations are included in the “Gas separator” unit. A detailed breakdown is required.
• Lifecycle Assessment: Similar to the TEA, several assumptions underlying the LCA remain unclear or insufficiently justified.
o The authors state that “Using a gate-to-gate attributional framework, the analysis shows that chemical upcycling reduces CO2 emissions by ~50% and ~66% relative to market-mix treatment and incineration.” However, it is unclear what the conventional “market-mix” technology includes—does it refer to mechanical recycling, pyrolysis, or another pathway? No details are provided. Importantly, mechanical recycling, the most mature and widely implemented recycling pathway, is not included in the comparison.
o It appears that pretreatment steps (e.g., collection, sorting, and transportation) are either omitted or assumed to be equivalent across all technologies. This assumption is unrealistic. Processes such as landfilling or incineration typically require less preprocessing than a technology that depends on a relatively pure, single-stream polyethylene feedstock. Assuming identical pretreatment burdens may bias the comparison in favour of the proposed technology. These system boundaries and assumptions should be explicitly defined and justified.
o As in the TEA, the energy demand and emissions associated with MEA-based CO2 capture (including regeneration) must be clearly incorporated into the LCA. It is currently unclear whether these contributions have been accounted for.
• Rebuttal, Comment B: The authors argue that “Density-based sorting can efficiently separate low-density polyolefins (PE, PP, PS) from higher-density plastics such as PET, PVC, and PU. PET can be depolymerized via established glycolysis or hydrolysis routes under milder conditions and is better processed separately.” While this may hold for virgin resins, consumer-grade plastics frequently contain fillers in significant quantities, which can increase density and compromise separation efficiency. This concern motivated our earlier question regarding the process tolerance to mixed or imperfectly sorted streams. The robustness of the system under such realistic conditions should be demonstrated or more critically discussed.
• It would strengthen the manuscript to incorporate results from realistic feedstocks (e.g., Fig. S30 and Fig. R2/R8) into Fig. 4, to demonstrate process robustness. Likewise, results from Fig. R3 should be included in the main text or Supporting Information to substantiate claims regarding multicomponent waste tolerance.
• Rebuttal, Comment E: The manuscript would benefit from the inclusion of a brief discussion of scale-up challenges and engineering considerations.
• In response to comment 11, the authors suggest that “In mixed polyolefin blends, the low-melting component (PE) softens and melts first and acts as a solvent phase into which higher-melting polymers (PP, PS) dissolve.” However, it is well established that polymers are often immiscible even when they possess similar hydrocarbon backbones. For example, polyethylene and polypropylene are generally thermodynamically immiscible in the melt and typically form phase-separated blends in the absence of compatibilizers or specially designed copolymers. Furthermore, in the present system, these polymers are dispersed in water, which would make homogeneous dissolution even less plausible. As written, the explanation appears inconsistent with established polymer physics. The authors should either provide direct evidence supporting this claim or revise the mechanistic interpretation accordingly.
• Rebuttal, Comment 14: In the FTIR spectra, only carbonyl peaks are labeled. O–H and C–O stretching bands should also be clearly indicated. Furthermore, the provided NMR spectra lack peak assignments. Protons associated with hydroxyl, aldehyde, and other functional groups typically resonate beyond 3 ppm, yet this region is not shown. The spectra should be expanded, properly annotated, and included in the Supporting Information to allow rigorous evaluation.
• Fig. S34: There appears to be a typo “Electic price” should be corrected to “Electric price.”
• Fig. S29b: The DSC thermograms presented correspond to the first heating cycle. For polymers, it is standard practice to report DSC data from the second heating cycle (Heat–Cool–Heat) to erase any prior thermal history and obtain reproducible melting and crystallization behavior.
• Abstract, Line 29: The term “fatty acids” should be replaced with “carboxylic acids,” as the reported products include a range of monoacids, diacids, and benzoic acid depending on the feedstock.
• Abstract, Lines 39-42: The reported numerical values should be updated to reflect the revised 60 kton/year basis.

Referee #3

(Remarks to the Author)
I co-reviewed this manuscript with one of the reviewers who provided the listed reports.

Version 2:

Reviewer comments:

Referee #2

(Remarks to the Author)
Review of “Catalyst-Free Valorization of Waste Plastics via Microdroplet-Interfacial Radical Scission”

We thank the authors for carefully considering our previous comments and for revising the manuscript accordingly. After reviewing the revised manuscript and the response-to-reviewers document, we believe that most of our concerns have been satisfactorily addressed, with the exception of a few points. Once these are adequately resolved, we recommend the manuscript for publication.

Specific comments are outlined below:
• Rebuttal, Comment 3: The authors attempt to explain the reactivity of PP based on its Vicat softening point, citing a range of ~80–120 °C, which is below the reaction temperature of 125 °C. However, values reported in the literature for PP are typically higher (~130–170 °C). This discrepancy raises the question of whether the reported value is specific to the PP used in this study. Given that the molecular weight of the PP employed here appears to be very low (~5 kDa), this may be possible. It would be useful to clarify this point. Additionally, it would be useful to comment on whether the same rationale would apply to isotactic PP of higher molecular weight, and whether the reaction would still proceed under the given conditions? The explanation provided for PS is satisfactory.
• Rebuttal, Comment 5.1: I thank the authors for the clarification regarding the market-mix technology and for providing additional comparison with mechanical recycling. While I understand the authors’ position that direct comparisons between chemical upcycling and mechanical recycling must be made with care, given that mechanical recycling preserves polymer chain integrity and therefore typically involves lower energy input and carbon emissions, I note that the other benchmarks used (landfilling, incineration, and market-mix scenarios) are likewise not directly comparable. A more rigorous comparison would ideally involve other chemical recycling or upcycling technologies that generate value from polyethylene (e.g., pyrolysis, gasification, catalytic cracking, hydrocracking). That said, I acknowledge that such an analyses may be beyond the scope of the present study. My original recommendation to include mechanical recycling was motivated by its status as one of the most mature and widely implemented waste management strategies. Its inclusion would provide a valuable baseline, enabling readers to better contextualize the performance of the proposed chemical approach. At the same time, elucidating the inherent limitations of mechanical recycling would further strengthen this context. Although mechanical recycling may exhibit lower energy and carbon intensity, this does not detract from the potential significance of the proposed technology. Rather, incorporating it as a benchmark would enhance the transparency and contextual robustness of the analysis. I therefore maintain my recommendation that mechanical recycling be included in the comparison.

• Rebuttal, Comment 9: The authors propose that “the role of PE is not to act as a thermodynamic solvent for PP or PS, but rather to generate low-molecular-weight, wax-like oligomers upon partial oxidative degradation, which mediate the dispersion of higher-melting polymers into the aqueous phase through wetting and swelling interactions.” To support this hypothesis, the authors present model experiments using C17 alkane as a proxy for the low-molecular-weight wax-like fraction and demonstrate that C17 can successfully disperse PP and PS. While this result is not unexpected, given that small alkanes are known to be effective solvents for polymers, it raises a question regarding the nature of the intermediates formed under the proposed reaction conditions. Based on the proposed mechanism, one might instead expect more polar species (e.g., alcohols, acids, or aldehydes/ketones). It is therefore unclear whether the same dispersive behavior would persist for such intermediates given their higher polarity. That said, the evidence provided in Fig. R5 demonstrating that the reaction proceeds at lower temperatures in the presence of PE is compelling. Additional experiments may not be strictly necessary; however, the authors should clarify this point or appropriately qualify their mechanistic interpretation.

Referee #3

(Remarks to the Author)
I co-reviewed this manuscript with one of the reviewers who provided the listed reports.

Open Access This Peer Review File is licensed under a Creative Commons Attribution 4.0 International License, which permits use, sharing, adaptation, distribution and reproduction in any medium or format, as long as you give appropriate credit to the original author(s) and the source, provide a link to the Creative Commons license, and indicate if changes were made.
In cases where reviewers are anonymous, credit should be given to 'Anonymous Referee' and the source.
The images or other third party material in this Peer Review File are included in the article’s Creative Commons license, unless indicated otherwise in a credit line to the material. If material is not included in the article’s Creative Commons license and your intended use is not permitted by statutory regulation or exceeds the permitted use, you will need to obtain permission directly from the copyright holder.
To view a copy of this license, visit https://creativecommons.org/licenses/by/4.0/

Point-by-point response to the reviewers' comments

We sincerely thank the reviewers for their insightful and comprehensive comments on our manuscript (ID: 2025-08-22021). We have carefully revised the manuscript in response to all comments and suggestions, and each point has been addressed in detail. Our responses to the reviewers' comments are provided below. We believe that these revisions have significantly improved the quality and clarity of the manuscript and that it now meets the criteria for publication.

Detailed reply to the reviewers' comments:

Reviewer #1:

This is a highly innovative paper on waste plastics valorisation. The author's approach is interesting and relevant as it avoids the use of catalyst and operates under milder conditions than those of conventional waste plastics conversion processes. The paper is in general well written, and the results and reaction mechanism discussion appear to be sound. Thus, data analysis and interpretation are effective and consistent. There are, however, some points that should be addressed and clarified. The next items have the objective of clarifying some aspects of the manuscript and of improving its scientific quality.

Response: We thank the reviewer for the positive and encouraging comments on our manuscript, especially for recognizing its innovation and relevance. We are also grateful for the constructive feedback provided, which has greatly helped us improve the clarity and overall scientific quality of the work. We have carefully addressed all the points below and believe that the manuscript has been significantly strengthened as a result.

Comment 1. *The authors should pay attention to some highly selective non-catalytic processes for waste plastics conversion into valuable chemicals. This is the case of plastics thermal cracking at high temperatures and with short residence times. The authors should note that under these conditions very high yields of light olefins (ethylene, propylene or butenes) were reported. Obviously, the conditions of this*

process are severe, however, it is important to remark that there are reasonable and selective alternatives for non-catalytic conversion of waste polymeric materials.

Response: We thank the reviewer for this helpful comment highlighting selective non-catalytic routes such as high-temperature thermal cracking. In response, we have added a concise discussion of catalyst-free pyrolysis to the main text and included a direct comparison between thermal cracking technologies and our approach (Table S1, Supplementary Information). This comparison clarifies the fundamental differences in reaction mechanism, target products, and application scope. We believe these revisions improve the contextual positioning of our work and more clearly convey its distinct contribution.

The specific revisions are outlined below:

Page 2, line 32 in the revised manuscript:

Non-catalytic thermal cracking is a mature and industrially practiced route for plastic valorization. Operated at high temperatures and short residence times, thermal pyrolysis can selectively produce light olefins (e.g., ethylene, propylene, and butenes) while benefiting from catalyst-free operation, rapid kinetics, and compatibility with continuous processing. However, its reliance on high-temperature radical chemistry limits reaction control and often leads to unstable product distributions, coke formation, reactor fouling, and high energy consumption (Table S1).

Page 47 in the revised Supplementary Information:

Table S1: *Comparison between Pore-Modulated Pyrolysis and Microdroplet-Interfacial Oxidation Strategy*

	Pore-Modulated Pyrolysis (*Nat. Chem. Eng.*, 2025, 2, 424 – 435)	This Microdroplet-Interfacial Oxidation
Reaction Type	Pyrolysis	Oxidation
Conditions	420–630 °C, Atmospheric/Inert pressure	125 °C, 2 MPa O_2

	Pore-Modulated Pyrolysis (*Nat. Chem. Eng.*, 2025, 2, 424－435)	This Microdroplet-Interfacial Oxidation
Primary Products	C_8–C_{18} hydrocarbon mixture	C_4-C_8 dicarboxylic acids
Product Selectivity	Up to 80.8% for C_8–C_{18} hydrocarbons	Up to 69.6% for C_4-C_8 diacids
Product Value	Fuels / Energy	High-value Platform Chemicals for polymers, food, and pharmaceuticals

Comment 2. *The interest of microdroplets reactor technology for plastics and other polymeric materials valorisations should be justified in the introduction.*

Response: We thank the reviewer for this insightful comment. In response, we have revised the Introduction to more clearly articulate the rationale for applying microdroplet concepts to plastic valorization. The revised text explicitly connects the distinctive features of microdroplet interfaces—such as enhanced interfacial fields and spontaneous radical generation—to the need for catalyst-free oxidation under mild conditions, thereby strengthening the conceptual foundation and clarity of the manuscript.

The specific revisions are outlined below:

Page 3, line 4 in the revised manuscript:

Microdroplets have recently emerged as unique chemical reactors due to their distinctive interfacial properties [6,7,26,31-37]. Intense electric fields (~10^9 V/m) at the microdroplet interface [36,38-41] can induce electron abstraction from hydroxide ions (OH^-), leading to the formation of hydroxyl radicals (•OH) and solvated electrons [42]. These highly reactive species enable efficient redox reactions under mild conditions, facilitating catalyst-free oxidation of small organic molecules such as toluene[43] and styrene[44] via ultrasonic emulsification or circulating droplet systems.

Page 3, line 15 in the revised manuscript:

Inspired by these considerations, we introduce a catalyst-free oxidative strategy that converts diverse polymer wastes—including polyolefins, rubbers, and mixed plastics—into value-added organic acids using only water and O_2 under mild conditions. The hydrophobic, long-chain nature of molten polymers is leveraged to generate dynamic water–oil interfacial microdroplets upon stirring, where hydroxyl radicals form spontaneously during oxidation (Fig. 1A). Continuous in situ •OH generation enables efficient C–H and C–C bond scission across chemically diverse polymers, while tolerating commercial additives and heterogeneous waste streams, offering a scalable and low-cost route for plastic upcycling.

Comment 3. *A basic characterization of the polymeric materials (proximate and ultimate analyses, ash composition…) used in this paper should be included in the supplementary material.*

Response: We thank the reviewer for this helpful suggestion. In response, we conducted a series of preliminary characterizations—including TGA/DSC, proximate (industrial) analysis, and elemental analysis—on the different PE samples used as representative substrates in this study. The corresponding results are provided in the revised manuscript and Supplementary Information (Fig. S29 and Tables S4–S5).

The specific revisions are outlined below:

Page 2 in the revised Supplementary Information:

Thermal analysis was conducted using a METTLER-TOLEDO TGA/DSC 1 or an equivalent synchronous thermal analyzer. All measurements were performed under a nitrogen atmosphere with a heating rate of 10 $K\cdot min^{-1}$. Proximate analysis was carried out using a 5E-MAG6700 Fully Automatic Industrial Analyzer. Prior to testing, the commercial PE samples were cooled with liquid nitrogen and ground into powder.

Page 37 in the revised Supplementary Information:

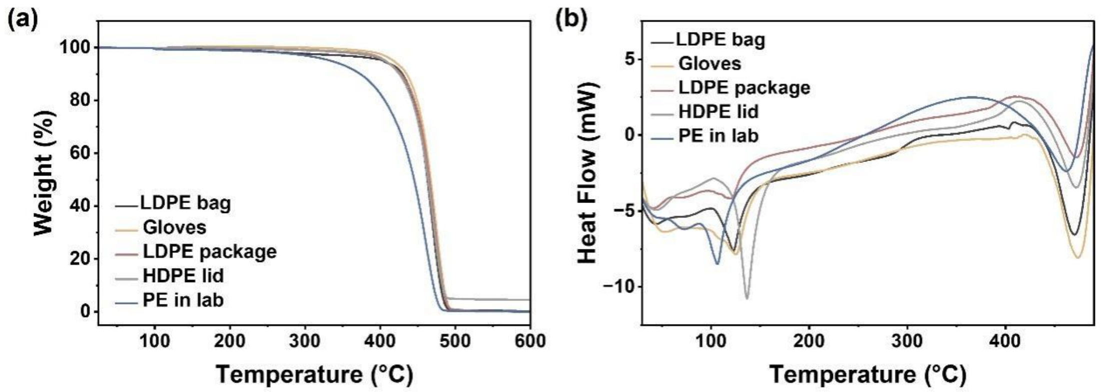

Fig. S29. (a) TG curves and (b) DSC curves of commercial PE and PE standard sample in lab. The test results of the five samples exhibited significant overlap, indicating that the thermal decomposition behaviors of the four commercial PEs are essentially analogous to that of standard PE in lab.

Thermogravimetric analysis (TGA) shows that all samples retain more than 99% of their mass up to approximately 350 °C, confirming the excellent thermal stability of PE. Significant weight loss occurs between 400 °C and 500 °C, which can be attributed to random scission of the polymer chains and the release of small hydrocarbon molecules. The final residue was minimal (close to 0%), consistent with the fact that PE contains almost no inorganic components (Fig. S29a and Table R4&5). The small amount of residue observed is likely due to trace additives or experimental noise. Differential scanning calorimetry (DSC) results show typical PE melting endothermic peaks below 150 °C for all samples. The laboratory PE standard exhibits a slightly lower apparent melting temperature than the commercial PE samples, which can be explained by differences in crystallinity and thermal history resulting from industrial processing (Fig. S29b).

Overall, the thermal behaviors of the five PE samples largely overlap, indicating that the four commercial PE materials have decomposition characteristics very similar to those of the laboratory PE standard.

Page 50 in the revised Supplementary Information:

Table S4. Proximate analysis of commercial PE and PE standard sample in lab.

	$M_{ad\%}$	$A_{ad\%}$	$V_{ad\%}$	$FC_{ad\%}$

LDPE bag	0.03	0.22	99.71	0.04
Gloves	0.31	1.07	98.56	0.06
LDPE package	0.07	0.32	99.58	0.03
HDPE lid	0.15	3.32	96.14	0.39
PE in lab	0.07	0.12	99.74	0.07

$\mathbf{M}_{ad\%}$: Moisture, air-dried; $\mathbf{A}_{ad\%}$: Ash, air-dried; $\mathbf{V}_{ad\%}$: Volatile matter, air-dried; $\mathbf{FC}_{ad\%}$: Fixed carbon, air-dried

Industrial analysis further shows that the volatile content of all samples exceeds 96%, in good agreement with the TGA results for PE. Among the samples, the lid materials show the highest ash content (3.32%), suggesting the presence of more inorganic fillers (such as calcium carbonate or titanium dioxide), which is reasonable given its need for higher mechanical rigidity. In contrast, the PE standard sample has the lowest ash content (0.12%), indicating the highest purity.

Table S5. Ultimate analysis of commercial PE and PE standard sample in lab.

	$\mathbf{C}_{ad\%}$	$\mathbf{H}_{ad\%}$	$\mathbf{N}_{ad\%}$	$\mathbf{St}_{ad\%}$	$\mathbf{O}_{ad\%}$
LDPE bag	88.02	9.46	0.03	0.94	1.30
Gloves	86.94	9.71	0.12	0.23	1.62
LDPE package	86.31	10.12	0.01	0.60	2.57
HDPE lid	86.02	9.30	\	0.20	1.01
PE in lab	86.89	10.46	\	1.17	1.29

$\mathbf{C}_{ad\%}$: Carbon, air-dried; $\mathbf{H}_{ad\%}$: Hydrogen, air-dried; $\mathbf{N}_{ad\%}$: Nitrogen, air-dried; $\mathbf{St}_{ad\%}$: Sulfur, total, air-dried; $\mathbf{O}_{ad\%}$: Oxygen, air-dried

Elemental analysis confirms that carbon and hydrogen are the main elements in all samples, although some differences are observed. The plastic sealing bags contain a relatively high oxygen content (2.57%), likely due to oxygen-containing additives used to improve flexibility. Disposable gloves contain trace nitrogen (0.12%), possibly originating from antistatic agents. The PE standard sample shows a relatively higher sulfur content (1.17%), which may result from residual catalysts or antioxidants.

Comment 4. *It seems that there is a mistake concerning gas product analysis, the*

*authors included the following sentence: "The concentrations of CO and CO2 were determined by GC-FID equipped with a converter and a packed column (TDX-01, 2 m*3 mm*2 mm)". However, the FID detector cannot analyse CO and CO2.*

Response: We thank the reviewer for drawing attention to the important point regarding gas analysis. As noted by the reviewer, a conventional FID does not directly detect CO or CO_2. In our measurements, this limitation was addressed by incorporating a catalytic methanizer upstream of the FID, which converts CO and CO_2 into methane over a nickel catalyst at elevated temperature (~350 °C).

$CO + 3H_2 \rightarrow CH_4 + H_2O$

$CO_2 + 4H_2 \rightarrow CH_4 + 2H_2O$

The resulting methane is then detected by the FID, enabling quantitative analysis of CO and CO_2. This GC-FID configuration with methanization is a well-established and widely used approach. We have revised the Methods section to clarify this experimental setup.

The specific revisions are outlined below:

Page 3 in the revised Supplementary Information:

The concentrations of CO and CO_2 were determined using GC-FID equipped with a methanization converter, which reduces CO and CO_2 to CH_4 over a Ni catalyst for subsequent FID detection, and a packed column (TDX-01, 2 m*3 mm*2 mm).

Comment 5. *Why was the liquid product esterified? What is the impact of this process in oil composition? Is not possible to directly analyse raw oil? Please clarify.*

Response: We thank the reviewer for raising this point. Because the liquid products are dominated by highly polar C4–C8 dicarboxylic acids, direct GC analysis suffers from peak tailing, inconsistent detector responses, and potential damage to GC components. To ensure reliable separation and accurate quantification, the acids were therefore derivatized to their methyl esters prior to GC analysis, as detailed in the Supplementary Information.

The specific revisions are outlined below:

Page 3 in the revised Supplementary Information:

The liquid products are dominated by strongly polar, saturated dicarboxylic acids (predominantly C_4–C_8 diacids). To enable more accurate quantification of the individual products, the carboxylic acids were derivatized into their corresponding methyl esters using trimethylsilyl diazomethane (TMS–CHN_2) under mild conditions prior to GC analysis.

Comment 6. *This sentence included in the experimental section is unclear: "Solid products. After centrifugation, the insoluble solid was dried at 80 overnight, and then weighed and compared with the mass of the catalysts loaded before the reaction". The authors stated that no catalyst was used in the process.*

Response: We thank the reviewer for noting this point. The question originated from an earlier draft of the Methods section and is not applicable to the experiments reported here, as no catalysts were used in this study. We have revised the Methods and Supplementary Information to clarify this and to prevent any potential confusion.

The specific revisions are outlined below:

Page 4 in the revised Supplementary Information:

Solid products. After centrifugation, the insoluble solid was dried at 80 °C overnight and then weighed to determine the mass of the solid residue. The conversion was calculated by comparing the mass of the residue with the initial mass of the polymer.

Comment 7. *The molecular weight of the PE used is very low, it is orders of magnitude lower than that typically used in commercial polymers. What is the impact of this fact in process performance (selection of process conditions, plastics conversion, reaction rate or product distribution)?*

Response: We thank the reviewer for raising this important point. Although a low-molecular-weight PE (Mn ≈ 1700 Da) was employed as a convenient model substrate for mechanistic studies, polymer molecular weight does not negatively affect the overall process performance.

To address this concern, we expanded the substrate scope to include several

commercial PE grades, including ultra-high-molecular-weight polyethylene (UHMWPE, Mn ≈ 6 × 10^6 Da), which exhibited comparable conversions and diacid yields under identical conditions (Fig. 4A, B).

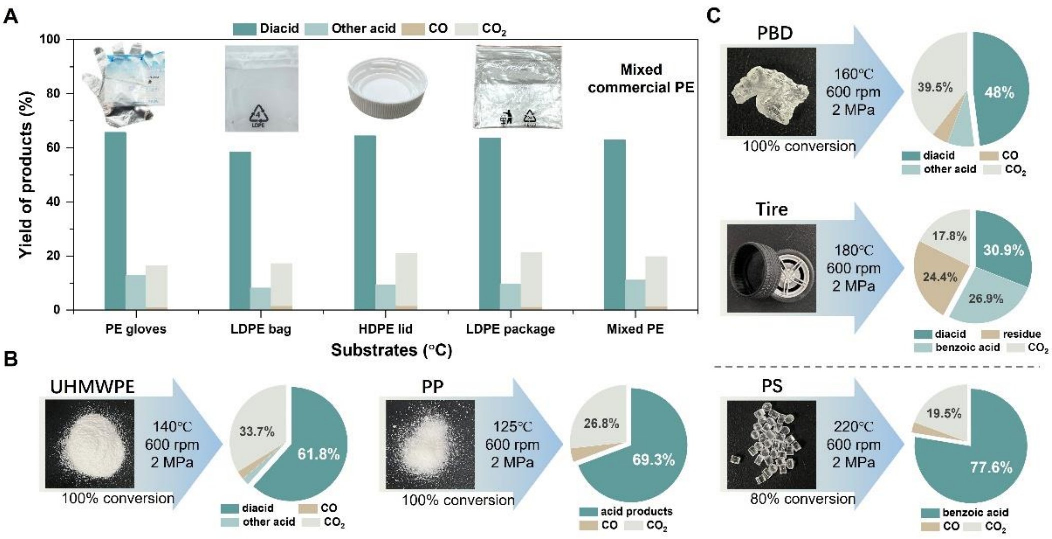

Fig. 4. The substrate expansion of the catalyst-free PE oxidation strategy. Performance evaluation using (A) different commercial PE: PE gloves, LDPE bag, HDPE lid, LDPE package and mixed commercial PE; (B) different polyolefins: UHMWPE, PP, PS; (C) rubber products: PBD, real-life tire.

Comment 8. *A critical aspect in this study is the selection of process conditions. The study was carried out at low temperature and high oxygen pressure, however, the selection of these conditions was not commented or justified.*

Response: We thank the reviewer for this comment. The reaction conditions (125 °C, 2 MPa O_2, 600 rpm stirring, and sufficient water) were chosen based on systematic optimization (see Supplementary Information, Fig. S16–S20). In brief: (i) 125 °C ensures PE melting and efficient dispersion into microdroplets, which is critical for hydroxyl radical generation and high conversion, while higher temperatures (>140–160 °C) reduce diacid selectivity and increase CO_2 formation (Fig. S18); (ii) increasing O_2 pressure improves conversion and diacid yield up to ~2 MPa, reflecting O_2's role in sustaining the interfacial radical cycle (Fig. S17, S13); (iii) adequate water volume and stirring are essential to form well-dispersed microdroplet–microbubble interfaces, as yields drop sharply under low water conditions (Fig. S20). To clarify these points, we

have added a concise summary in the main text, emphasizing the rationale behind the selected conditions.

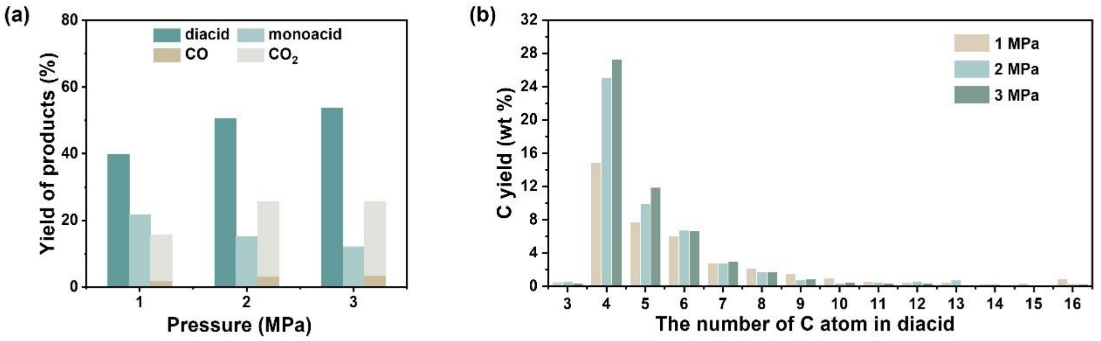

Fig. S17. (a) The yield of products of PE oxidation under different pressure of O_2 at 125 °C. (b) Distribution of diacid generated under different pressure of O_2 at 125 °C.

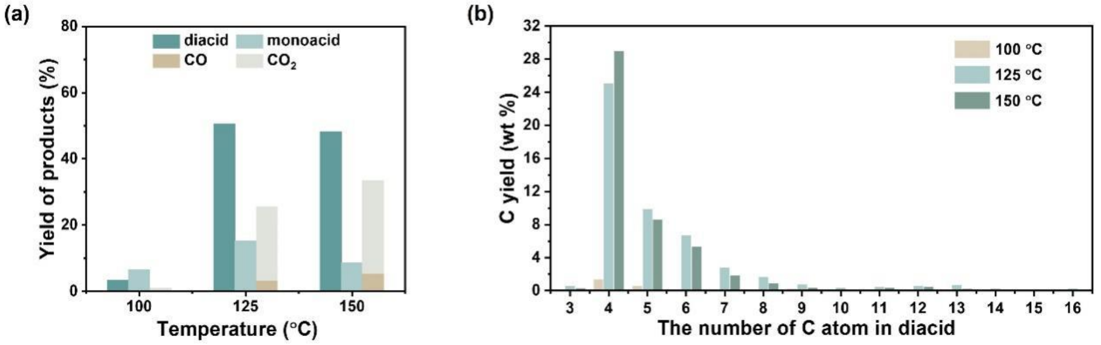

Fig. S18. (a) The yield of products of PE oxidation under different temperature under 2 MPa O_2. (b) Distribution of diacid generated under different temperature under 2 MPa O_2.

The specific revisions are outlined below:

Page 8, line 6 in the revised manuscript:

PE degradation was investigated under varied conditions by adjusting temperature, stir speed and oxygen pressure to control oxidation and optimize diacid yield (Figs. S16-S19). Optimal performance was achieved at 125 °C and 2 MPa O_2 with vigorous stirring (600 rpm), which promotes polymer melting and interfacial dispersion, thereby enhancing •OH generation while avoiding excessive over-oxidation.

Comment 9. *Does Figure 1 b correspond to a dicarboxylic acids yield of 69.6%? It seems that in this photo the plastic conversion is rather low, it is possible to observed a solid plastic.*

Response: We thank the reviewer for this comment. Fig. 1b indeed corresponds to a reaction achieving a 69.6% diacid yield. The left image shows the initial PE powder, while the right image depicts the fully liquefied post-reaction mixture. The apparent "solid" in the right image is not unreacted polymer but small O_2 bubbles adhering to the vial walls during depressurization. Analytically, complete PE conversion (>99%) is confirmed.

To prevent any misunderstanding, we have revised the figure caption to clearly indicate the pre- and post-reaction states and the absence of solid residue.

The specific revisions are outlined below:

Page 5, line 10 in the revised manuscript:

(**B**) Comparison of reaction vials before and after PE oxidation. (Left: initial PE powder; Right: post-reaction mixture with no visible solid residue)

Comment 10. *The authors evaluated the performance of microdroplet technology for the valorisation of different types of real polymers (polyolefins, PS or even waste tires). The composition of these materials is very different, and a completely different reactivity and reaction mechanism can be expected for each feedstock. In fact, the conditions used with these polymeric materials were different. The authors should discuss the role played by the polymeric material nature on the selection of suitable process conditions and process performance.*

Response: We thank the reviewer for this important suggestion. The chosen reaction temperature is primarily dictated by the thermal and phase behavior of each polymer, ensuring they are molten or well-dispersed to form water–oil interfacial microdroplets. These interfaces are essential for the generation of •OH radicals, which are sufficiently reactive to initiate oxidation at the relevant C–H or C=C sites across different polymers (see Page 10 and Fig. S32). According to your suggestions, we have added further explanations for Figure 4B in the manuscript and for Figure S32 in the Supplementary Information.

The specific revisions are outlined below:

Page 10, line 3 in the revised manuscript:

The process was extended to other polymers by tuning the temperature to match their melting or dispersion behavior (Fig. 4B). For example, UHMWPE was fully converted at 140 °C, yielding 61.8% diacids. Although the higher temperature slightly increased CO_2 production, the outcome still affirms the method's effectiveness. Since the oxidation is exothermic, the temperature can be set slightly below the polymer's melting point to limit over-oxidation. Polypropylene and polystyrene were selectively converted to acetic acid and benzoic acid at 125 °C and 220 °C, respectively, driven by the reactivity of tertiary sp^3 carbons (Fig. S32).

Page 40, line 1 in the revised Supplementary Information:

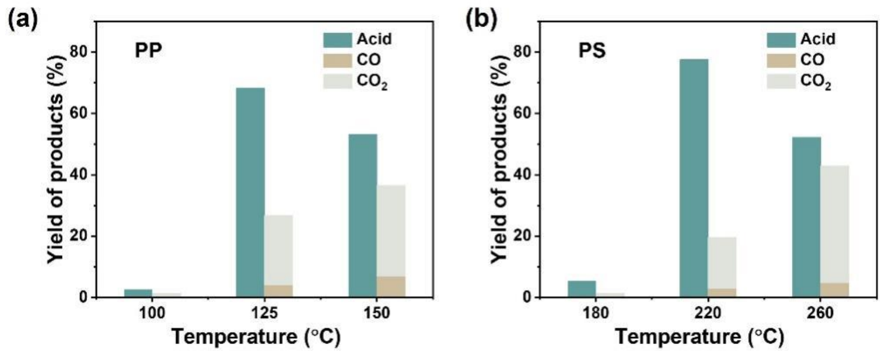

Fig. S32. Product yields from (a) polypropylene (PP) and (b) polystyrene (PS) oxidation at different temperatures under 2 MPa O_2.

Polypropylene (PP): The tertiary sp^3 C–H bonds in PP facilitate hydrogen abstraction, forming stable tertiary radicals. These radicals preferentially undergo β-scission or further oxidation, yielding mainly low-molecular-weight monocarboxylic acids such as acetic acid. Consequently, PP produces a narrower distribution of small monocarboxylic products compared with linear polyolefins.

Polystyrene (PS): Benzylic C–H bonds are readily abstracted to form benzyl radicals, stabilized by adjacent aromatic rings. Subsequent O_2 addition and stepwise oxidation via benzyl alcohol and aldehyde intermediates ultimately yield benzoic acid, explaining the prevalence of aromatic monocarboxylic acids in PS oxidation.

Comment 11. *The technology readiness level of this study is low, the study was carried*

out at lab scale, with some experiments performed in a batch unit operating with 300 g of sample. Considering this fact, it is probably too optimistic the incorporation of a techno-economic analysis of the process.

Response: We thank the reviewer for this thoughtful comment. We agree that the original TEA based on a 200 kt·yr^{-1} plant may be overly optimistic at this early stage. In response, we revised the analysis to a more realistic 60 kt·yr^{-1} scale, consistent with typical pilot- and early commercial depolymerization facilities (3–100 kt·yr^{-1}, with most below 60 kt·yr^{-1}, *Energy Environ. Sci.,* 2023, 16, 3638–3653; *Science* 2023, 381, 660–666). This provides a more conservative and practical assessment while retaining the key insights into cost structures.

Although our study remains at the lab scale, the 300 g batch experiments represent one of the larger scales used in academic studies to support preliminary TEA for chemical plastic upcycling. We are actively exploring scale-up in collaboration with industrial partners, but full integration with dedicated equipment and infrastructure requires several years. The revised TEA therefore offers a rigorous, transparent, and realistic evaluation for near-term scale-up, as recommended. All results have been updated in the manuscript and SI.

The specific revisions are outlined below:

Page 11, line 2 in the revised manuscript:

Techno-economic analysis (TEA) confirmed the robust economic viability of the catalyst-free polyolefin degradation process (Fig. S33). At plant scale the effluent is cooled and flashed to separate a gas stream (O_2, CO_2 and CO) and a liquid phase; CO is combusted for energy recovery and the flue gas is subjected to CO_2 capture while process water is recovered and recycled. The heavier liquid fraction is upgraded via dehydration/distillation to convert C_4–C_5 diacids to their anhydrides, followed by fractional distillation to obtain purified products. Evaluated at 60 kt yr^{-1}, the design requires a total capital investment of $141 million (Tables S6–S10) and yields an annual net profit of $74.0 million after tax, giving a payback of ~3.1 years without government subsidies (Fig. 5D). The breakeven point is ~7.8 kt yr^{-1}, demonstrating sustained

economic viability across an exceptionally broad spectrum of polymer waste input volumes. This remarkably low threshold signifies operational resilience to feedstock volatility and supports potential deployment at sub-nominal capacities. Our sensitivity analysis shows that product price and total capital investment are the dominant drivers of NPV, while reaction residence time also strongly influences NPV because it controls reactor volume (Fig. S34). These results support the characterization of the process as capital-intensive with economics that depend on high product value and efficient reactor design.

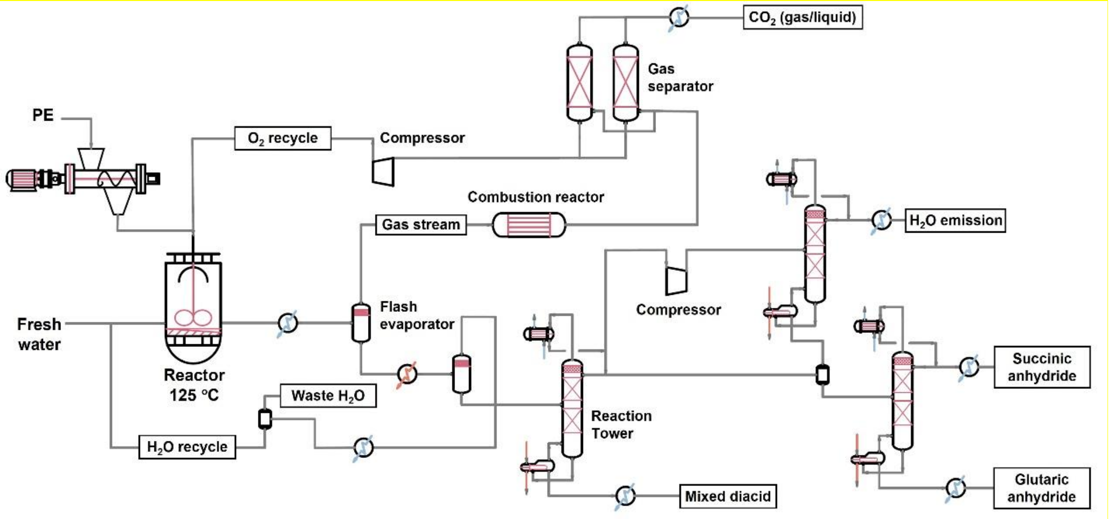

Fig. S33. Proposed process flow diagram to produce diacid from waste PE via catalyst-free oxidation.

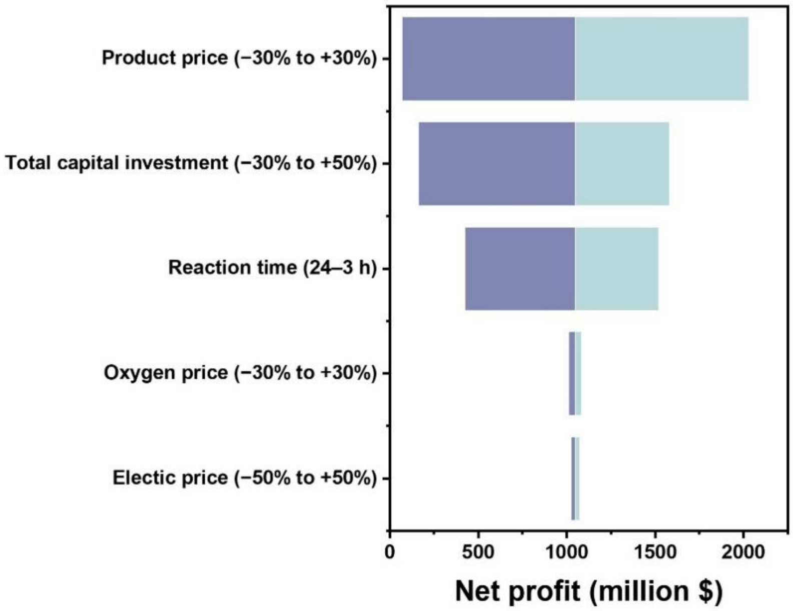

Fig. S34. One-way sensitivity analysis of net present value (NPV).

Comment 12. *The real challenges of the plastics valorisation route should be discussed. One of the main concerns is the slow reaction rate of the process, thus, reaction time extremely high (several hours). It is to note that other thermochemical processes for waste plastics valorisation have reaction times from few minutes to some seconds. Moreover, the process was studied in batch regime, the scale up of the process usually requires continuous operation. Is it possible to operate this process in continuous regime?*

Response: We thank the reviewer for this important comment. We agree that reaction time and continuous operation are key considerations for practical plastic valorization. While high-temperature pyrolysis offers very short residence times and high throughput, it also faces well-recognized challenges—including broad product distributions, high energy demand, limited mass transfer of molten plastics, and extensive downstream processing to produce specification-grade chemicals (e.g., fractionation, sequential catalytic steps, and purification; 10.1126/science.adh0993; 10.1126/science.adh1853). Consequently, short pyrolysis residence times do not directly translate to high overall yields of value-added products.

In contrast, our study explores a fundamentally different, mild oxidative-interfacial route that generates structurally defined oxygenates and emphasizes mechanistic understanding of microdroplet-mediated •OH generation. For this purpose, batch operation provides precise control over temperature, oxygen pressure, and droplet characteristics, enabling detailed investigation of reaction pathways and early-stage scale-up.

We fully agree that continuous operation is essential for future process development. To this end, we have designed a plug-flow reactor concept (Fig. R1), which enables continuous microdroplet generation, segmented temperature control, precise residence time management, and scalable operation through channel extension or parallelization. While full implementation requires substantial time for equipment setup and safety validation, these continuous-mode studies form the core of our ongoing work and will guide future industrial translation.

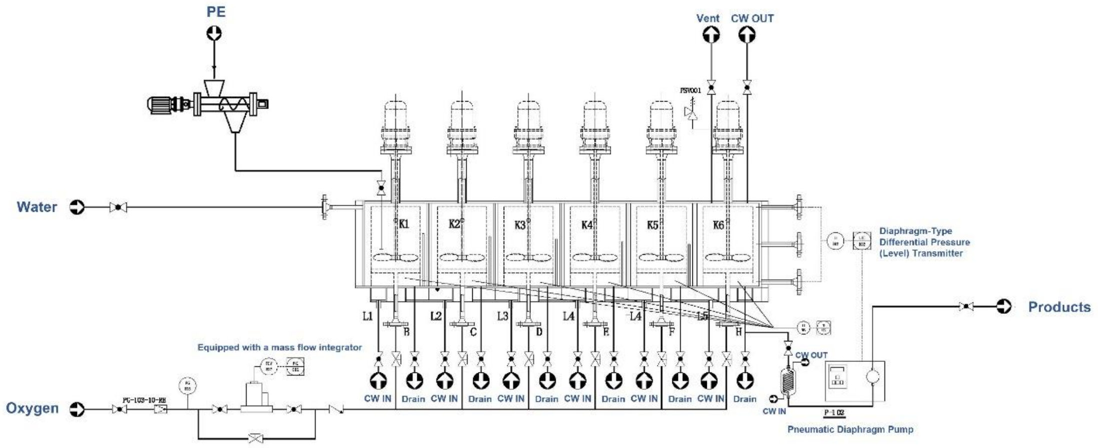

Fig. R1. Process flow diagram of the plug-flow reactor designed for continuous microdroplet oxidation of polymers.

This reactor enables the oxidative upcycling of polyolefins in a spatially and temporally controlled manner. Polymers are continuously fed via a heated screw and feeder, where mechanical agitation disperses the polymer into the aqueous phase, forming polymer – water interfacial microdomains. Oxygen is supplied through a mass-flow-controlled gas line along the reactor length, ensuring sufficient oxidant availability while minimizing local over-oxidation. As the reaction mixture progresses, gradual chain scission and oxygen incorporation produce oxygenated oligomers and ultimately short-chain carboxylic acids. This design allows continuous operation with controlled reaction extent, maintaining the diacid selectivity observed in batch experiments and providing a clear route toward scalable implementation.

Comment 13. *Another relevant question is the necessity of feeding the polymeric material as a fine powder. This is a major problem, on the one hand, it could be challenging the grinding of some types of waste polymers. On the other hand, it is expensive and requires a lot of energy (extremely high for waste tires).*

Response: We thank the reviewer for raising this practical point. Using fine powder is not a requirement of our process; complete conversion with high diacid yield is readily achieved using small polymer fragments, such as cut packaging waste (Fig. R2). In the lab, powders were employed mainly for mechanistic convenience and analytical consistency. In practice, the continuous plug-flow reactor under development is

compatible with small chips or fragments, which can be fed via a heated screw feeder after low-energy shredding. For materials like end-of-life tires, size reduction is already standard industrial practice and requires less energy than fine grinding of polyolefins. The critical operational requirement is that the polymer reaches a molten or sufficiently softened state to enable effective dispersion into water as interfacial microdroplets.

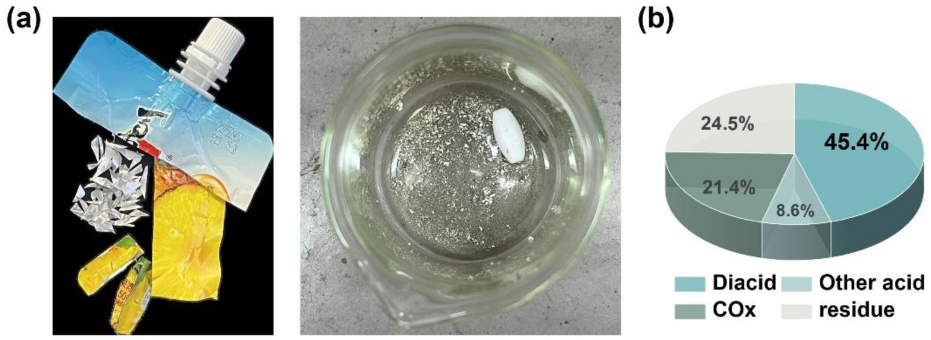

Fig. R2. (a) The photo of the packaging waste (cut into small pieces) and post-reaction solution (the solid residue is Al foil). (b) The product yields from packaging waste conversion.

Reviewers 2 and 3:

The authors present a catalyst-free strategy to upcycle plastics (primarily polyolefins) into short-chain diacids (C4–C8) using only water and O2 under mild conditions (125 °C, 2 MPa O2). This approach leverages microdroplet chemistry, exploiting the long-chain structure and hydrophobic nature of molten plastic wastes to form critical water-oil interfacial microdroplets that spontaneously generate •OH radicals, thereby facilitating oxidative polymer cleavage. Mechanistic studies employing isotopically labelled water, combined with comprehensive characterization techniques, confirm the key role of H_2O in mediating the oxidative degradation pathway. The strategy is further extended to various polyolefin feedstocks (both virgin and post-consumer) and rubber tires, and is shown to be compatible with natural water sources such as tap water and seawater. Moreover, the process was successfully scaled up to a 300 g batch to demonstrate its scalability. Overall, this work presents a promising route for converting plastic waste into value-added oxygenates under remarkably mild conditions. While the manuscript is well written and demonstrates clear novelty and rigor, with results supported by convincing evidence, several major points should be addressed before the work can be considered for publication in Nature.

Response: We sincerely thank the reviewer for the thorough and insightful evaluation of our work. We appreciate the recognition of the novelty, mechanistic depth, and broad applicability of our catalyst-free microdroplet oxidation strategy, as well as its potential for converting diverse plastic wastes into value-added oxygenates under mild conditions. We also acknowledge the reviewer's constructive major concerns. In the revised manuscript, we have carefully addressed all points raise. Detailed responses to each comment are provided below, and all corresponding revisions have been incorporated into the manuscript and the Supplementary Information.

Comment A. *A major concern lies in the CO2 emissions associated with the process. According to the authors' results, approximately 25% COx (predominantly CO2) is generated, with values rising to 30－45% for certain polymers (e.g., PP and PBD). This raises significant questions regarding the environmental footprint of the approach.*

The reported range corresponds to roughly 0.78 – 1.35 kg CO2-eq per kg of polymer, potentially offsetting the sustainability benefits of the upcycling process. Moreover, the extended reaction durations (12 – 24 h on a small scale and >48 h on a larger scale) and the use of excess water likely contribute additional indirect emissions from energy consumption and product separation. A comprehensive life cycle analysis would therefore be essential to evaluate the overall sustainability and practical viability of the process.

Response: We thank the reviewer for raising this important point regarding CO_2 emissions, energy demand, and overall environmental footprint. To address this, we performed a gate-to-gate life-cycle assessment (LCA) of the proposed process (see Supplementary Information). The analysis shows that, compared with conventional incineration or market-mix disposal, our chemical upcycling route reduces net CO_2 emissions by ~50–66% and delivers a net benefit in cumulative energy demand when credits for displaced diacid and CO_2 production are considered (Fig. 5E). Sensitivity analyses indicate that factors such as cooling energy, CO_2 capture efficiency, and oxygen supply can further optimize environmental performance.

While some direct CO_2 formation and water use contribute to the footprint, these are fully accounted for in the LCA. Ongoing reactor design improvements—including plug-flow configurations and enhanced gas-liquid contacting—are expected to reduce residence times and energy consumption at scale. Overall, the results indicate that, despite unavoidable CO_2 formation during oxidative cleavage, the process is environmentally competitive and can deliver net sustainability benefits. We have clarified and expanded the LCA discussion in the revised manuscript and Supplementary Information.

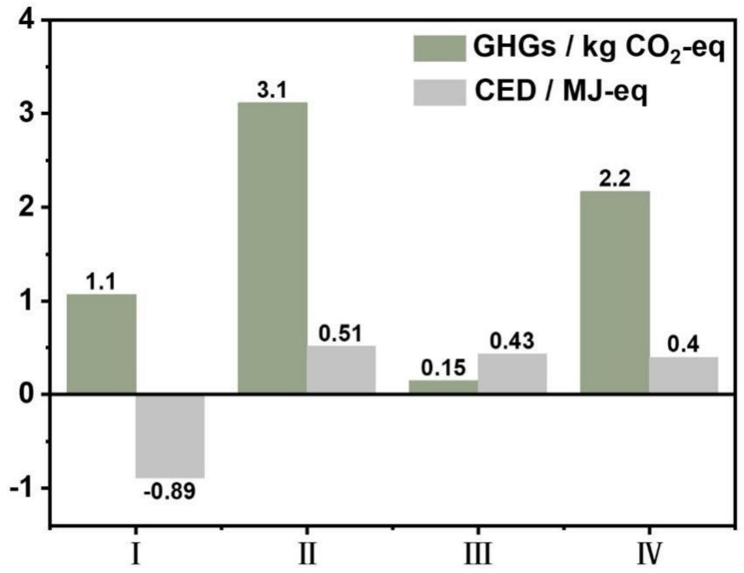

Fig. 5E. Greenhouse gas emissions (GHGs) and cumulative energy demand (CED) for the treatment of 1 kg of plastic via: (I) the proposed upcycling technology, (II) incineration, (III) landfilling, and (IV) the conventional market-mix technology.

The specific revisions are outlined below:

Page 11, line 20 in the revised Manuscript:

Life-cycle assessment (LCA) was conducted to benchmark the environmental performance of the proposed upcycling process against conventional PE waste management routes. Using a gate-to-gate attributional framework, the analysis shows that chemical upcycling reduces CO_2 emissions by ~50% and ~66% relative to market-mix treatment and incineration, respectively (Fig. 5E), while providing a net benefit in cumulative energy demand through system expansion that credits displaced diacid and liquid CO_2 production. Endpoint indicators for human health, ecosystem quality, and resource scarcity consistently favor the oxidative upcycling route.

Comment B. *Another concern pertains to the process feasibility for mixed waste upcycling. Although the authors demonstrate extension to several polymers (PP, PBD, PS), the reaction temperature appears to depend strongly on the polymer melting point. Operating at higher temperatures, however, risks product overoxidation and increased CO2 formation. Real-world plastic waste streams are highly heterogeneous and typically contain polymers such as PET, PVC, and PU in addition to polyolefins. The*

authors should provide evidence or discussion demonstrating the robustness of the process toward such mixed and additive-rich feedstocks to support claims of practical applicability.

Response: We thank the reviewer for raising this important point regarding the treatment of heterogeneous plastic waste streams. We fully agree that real-world plastics often contain polymers with distinct thermal and chemical properties, which must be considered for process robustness.

In practice, preliminary separation is advisable. Density-based sorting can efficiently separate low-density polyolefins (PE, PP, PS) from higher-density plastics such as PET, PVC, and PU. PET can be depolymerized via established glycolysis or hydrolysis routes under milder conditions and is better processed separately. PVC generates corrosive species and char upon oxidation, making it unsuitable for co-processing, while PU is more appropriately recycled via selective cleavage of urethane and amide linkages.

Accordingly, we focused on mixed low-density polyolefins. In our interfacial oxidative system, polymer melting and dispersion into water to form microdroplets are essential for •OH-driven interfacial chemistry. We optimized reaction conditions using mixed-polymer model systems (Fig. R3), demonstrating that polyolefin-dominated streams can be effectively upcycled, while non-polyolefin fractions are best treated via dedicated routes matched to their chemical characteristics.

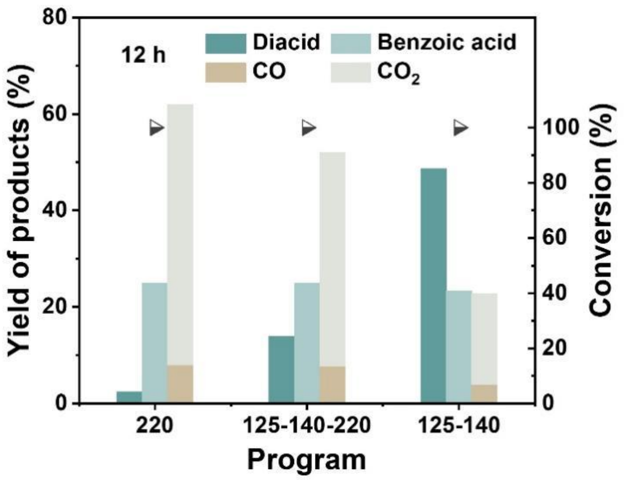

Fig. R3. The product yields from mixed polyolefins (PE, PP, PS) under different heating

protocols.

Comment C. *Lastly, although the manuscript shows successful deconstruction of certain consumer-grade plastics, further clarification and data are needed regarding the tolerance of this catalyst-free system to additives, fillers, and other impurities. Additional discussion or experiments (as noted in the specific comments) would help to substantiate the claimed generality of the process.*

Response: We appreciate the reviewer's attention to the role of additives, fillers, and impurities commonly present in commercial plastics, which is indeed an important consideration for evaluating the practical robustness of the process. In addition to demonstrating the deconstruction of a range of real-world plastic wastes, we further assessed the tolerance of our catalyst-free system by deliberately introducing representative organic and inorganic additives into the reaction mixture. These included zinc-calcium stabilizers, ultraviolet absorbers, hindered amine light stabilizers (HALS), phthalate plasticizers, as well as inorganic fillers such as TiO_2 and $CaCO_3$. These experiments show that the interfacial microdroplet oxidation pathway remains operative in the presence of typical additive loadings, with overall conversion and product selectivity largely preserved. The corresponding results and discussion have been incorporated into the revised manuscript and the Supplementary Information.

The specific revisions are outlined below:

Page 9, line 20 in the revised Manuscript:

We additionally evaluated the effects of excess antioxidants, hindered amine light stabilizers (HALS), and representative inorganic impurities commonly found in commercial plastics (Figs. S30-31 and Table S4-5). Under typical formulation loadings, these additives had no detectable impact on either the reaction conversion or diacid yield, underscoring the robustness of the process.

Page 38 and 39 in the revised Supplementary Information:

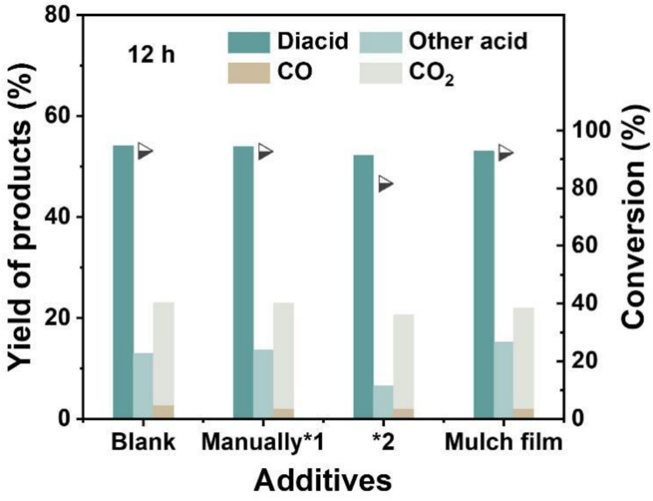

Fig. S30. The yield of products and PE conversion under different conditions of additives. (Manually add*1: 0.5 wt% BHT (a phenolic antioxidant), 0.5 wt% Bis(2,2,6,6-tetramethyl-4-piperidyl)sebacate (HALS), 0.4 wt% Irganox 1010 (Pentaerythritol tetrakis[3-(3',5'-di-tert-butyl-4'-hydroxyphenyl)propionate]) and 1 wt% N-(1,3-dimethylbutyl)-N'-phenyl-p-phenylenediamine (6PPD); *2: twice the manually added amount of * 1; Mulch film: agricultural film containing HALS)

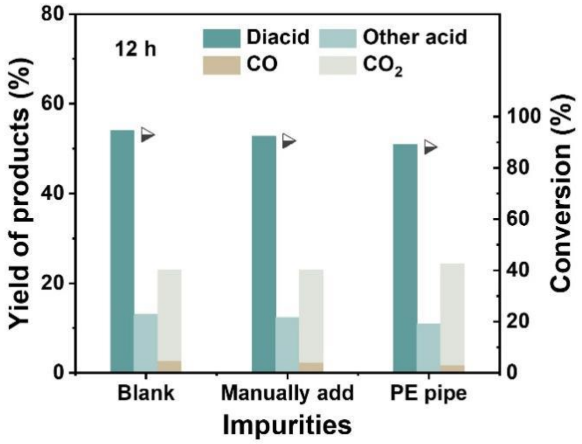

Fig. S31. The yield of products and PE conversion under different conditions of inorganic impurities. (Manually add: 5 wt% TiO_2 + 5 wt% $CaCO_3$ + 0.1 wt% ZnO; PE pipe: containing TiO_2)

Comment D. *Diacids are platform molecules. However, their production rate is about two orders of magnitude lower than that of plastics. How will the products from the conversion of most plastics be absorbed in the market?*

Response: We thank the reviewer for this important and forward-looking question. We

fully acknowledge that no single product pathway can, or should, be expected to absorb the entirety of global plastic waste.

Importantly, plastic production and utilization are not static. Trends such as reduced demand for single-use plastics, policy-driven requirements for recycled content, and the increasing adoption of biodegradable and bio-based materials are gradually reshaping both the volume and composition of plastics on the market. Within this evolving landscape, the relative role of oxygenated platform molecules such as diacids is likely to expand, but not to the extent of replacing all polyolefin-derived products.

Our approach is therefore not intended to convert most plastics into diacids. Rather, it is envisioned as one complementary pathway within a diversified portfolio of plastic waste management strategies, alongside mechanical recycling, energy recovery, and other chemical recycling routes. The most suitable valorization pathway will ultimately depend on regional waste composition, infrastructure, regulatory frameworks, and market demand.

Consistent with this view, we and others are actively developing parallel routes for polyolefin valorization that yield different product classes, such as light aromatics and fuels (Angew. Chem. Int. Ed., 2025, 64, e202506815 & e202424334), which can access much larger existing markets. Collectively, the coexistence of multiple, economically viable pathways—rather than reliance on a single product market—represents the most realistic approach to managing large and heterogeneous streams of plastic waste.

Comment E. *Most plastics are hydrophobic. How will the scale-up in hydrophobic water happen? How will mixing/interfacial contact occur at a large scale?*

Response: We thank the reviewer for this important practical question. Two aspects are central to understanding the conversion of hydrophobic plastics in our system and its translation to larger scales.

1. Behavior in batch operation (mechanistic and operational considerations).

When large amounts of hydrophobic polymers are introduced into an aqueous reactor, they initially form a separate molten or softened oil phase floating on the water. Under elevated temperature, pressure, and vigorous stirring, this phase rapidly fragments into

dispersed particles and microplastics. Micron-sized polymer droplets were directly observed during reaction sampling (Supplementary Fig. S10, Supplementary Movies). Importantly, the interfacial environment is highly dynamic: droplets are continuously oxidized and consumed at the interface, while new droplets and softened fragments are generated through melting and mechanical shear. This continuous renewal of interfacial area sustains the reaction over time, even at high polymer loadings.

2. Engineering strategies for scale-up (enhancing and maintaining interfacial contact).

At larger scales, droplet formation is no longer solely passive; established industrial mixing and contacting strategies can be employed:

Continuous plug-flow feeding: A plug-flow configuration with a melt/feeding module and inline emulsification (Fig. R1) stabilizes droplet formation, limits solids inventory, and allows precise residence-time control, facilitating scale-up.

High-shear and turbulent agitation: A combined impeller strategy can be used: (i) a high-shear impeller (e.g., serrated turbine) in the lower reactor zone fragments molten polymer into fine droplets; (ii) an upper axial-flow impeller circulates material to the shear zone, minimizing dead zones; (iii) gas entrainment along the shaft enhances O_2 or air distribution, improves gas-liquid mixing, reduces slurry density, and promotes sustained oil–water contact.

Additional interfacial intensification: If required, Venturi or other hydrodynamic mixers can generate intense shear and turbulence to produce fine droplets and renew interfacial area. Small amounts of emulsifiers can further stabilize dispersed droplets. Such approaches are widely applied in industrial gas–liquid and solid–liquid–gas processes and are directly compatible with molten-polymer-in-water systems.

Operationally, the key controllable parameters are polymer melting/softening temperature, shear intensity, stirring rate, and oxygen (or gas) management. Collectively, these measures allow replication and intensification of the microdroplet interfacial environment observed in the lab at larger throughput, while maintaining energy efficiency and operational safety using established engineering practices.

The responses to the Specific Comments are as follows:

Comment 1. *Can the process be conducted using air instead of pure O2? This would greatly enhance the practicality and scalability of the method compared to relying on high-purity oxygen.*

Response: We thank the reviewer for highlighting this important point. We agree that using air instead of pure O_2 would enhance the practicality and scalability of the process. Provided the total pressure is sufficient to maintain an adequate oxygen partial pressure, the reaction proceeds stably, demonstrating the robustness of the underlying chemistry.

In our experiments, introducing compressed air at 2 MPa resulted in lower PE conversion and diacid yields (Fig. S16). Performance improved with increasing air pressure, and at 8 MPa the system approached the behavior observed under pure O_2. These results indicate that the reaction does not inherently require pure oxygen, but rather sufficient oxygen availability at the reactive interface.

The specific revisions are outlined below:

Page 25 in the Supplementary Information:

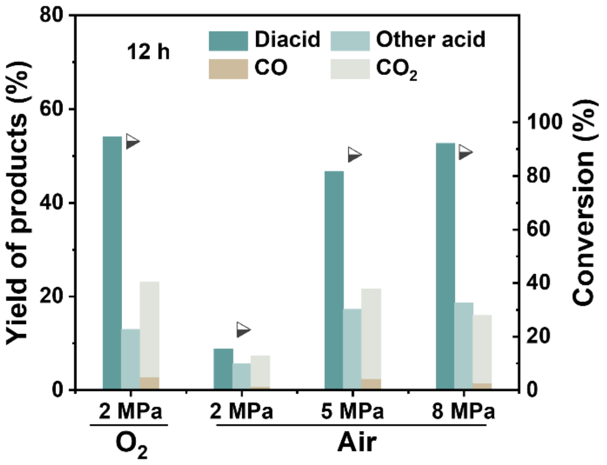

Fig. S16. Product yield and PE conversion under different atmospheres.

Comment 2. *In Table S1, the authors show that the absence of solvent or the use of organic solvents results in low PE conversion. However, the entry with 90% water + 10% methanol also shows almost no conversion. Can the authors explain this observation? Is this due to methanol acting as a radical scavenger? While this is a possible explanation, the tert-butyl alcohol experiments indicate that radical quenching*

only occurs at higher alcohol concentrations (~30 vol%). Further clarification on this discrepancy is needed. Can the authors clarify this discrepancy?

Response: Thank you for drawing our attention to this discrepancy. Upon carefully re-examining our experimental records, we identified that the corresponding entry in Table S2 was inadvertently transposed during the preparation of the Supplementary Information. The correct composition for this entry is 10% water and 90% methanol (rather than 90% water and 10% methanol). We apologize for any confusion this may have caused and have corrected the Supplementary Information accordingly.

The specific revisions are outlined below:

Page 47 in the Supplementary Information:

90% Methanol+10% Water	3.4%
70% Methanol+30% Water	11.7%
30% Methanol+70% Water	34.6%
10% Methanol+90% Water	62.9%

Comment 3. *Is it possible to tune the carbon number of the diacid products by adjusting reaction parameters? For example, could the process be directed toward forming higher-value diacids such as sebacic acid (C10), adipic acid (C6), or suberic acid (C8), instead of succinic acid?*

Response: We appreciate this insightful question. Based on our mechanistic analysis and systematic screening of reaction conditions (Figs. S16–S20), we find that the carbon-number distribution of diacid products is largely insensitive to variations in temperature, pressure, or reaction time. Across all conditions, C_4–C_6 diacids dominate, with yields decreasing steadily for longer chains.

This preference arises from the intrinsic •OH-initiated radical oxidation mechanism, which proceeds via highly favorable 1,5-hydrogen atom transfer (1,5-HAT) and related intramolecular rearrangements. As illustrated in Fig. S25, these pathways involve 6–8-membered transition states that are both kinetically and thermodynamically preferred, naturally biasing the system toward short-chain cleavage products (10.1007/s11426-

024-2252-9). Formation of longer-chain diacids (e.g., C_8 or C_{10}) would require higher-order H-shifts or less favorable β-scission events, which are strongly disfavored under the mild interfacial oxidation conditions used here.

Thus, while reaction parameters can modulate overall conversion, they do not substantially alter the intrinsic preference for C_4–C_6 diacids. This selectivity is advantageous practically, as these products are easier to separate and have broad industrial relevance.

In recent work, we have extended this approach by selectively introducing double bonds into PE through controlled functionalization. These defined reactive sites enable ozonolytic cleavage, allowing conversion of PE into longer-chain C_8–C_{20} dicarboxylic acids (Fig. R4).

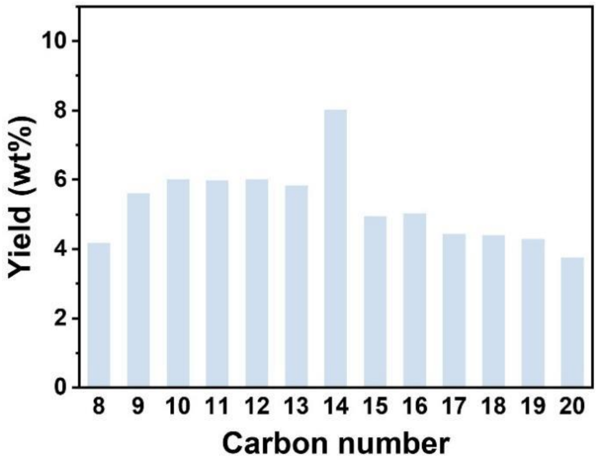

Fig. R4. The yield distribution of long-chain dicarboxylic acids (C_8–C_{20}) obtained from ozonolysis of functionalized PE.

The specific revisions are outlined below:

Page 8, line 25 in the manuscript:

Notably, C_4–C_6 diacids exhibited higher selectivity than longer-chain analogues across all tested reaction conditions, consistent with 1,5-HAT pathways proceeding through thermodynamically favored 6-8 membered transition states (Fig. S25).

Comment 4. *Since radical chemistry is unselective, what controls the selectivity to the small diacids and mainly to C4?*

Response: We thank the reviewer for this important point. While “radical chemistry”

often implies broad, unselective fragmentation at high temperatures, the radical pathway in our system is fundamentally different and intrinsically biased toward short diacids, particularly C_4. This selectivity arises from the combination of low-temperature •OH initiation and favorable intramolecular H-transfer. Key aspects include:

1. Mechanistic bias of •OH initiation (distinct from pyrolysis).

The reaction begins with hydroxyl-radical hydrogen abstraction or addition at accessible C–H sites, forming carbon-centered radicals that react with O_2 to generate peroxyl intermediates (ROO•). Unlike high-temperature pyrolysis, which causes indiscriminate C–C cleavage and broad product distributions, the •OH pathway targets specific hydrogen sites, directing controlled oxygenation rather than random bond rupture.

2. Dominant 1,5-HAT and favorable transition states.

Key radical rearrangements proceed via 1,5-hydrogen atom transfer (1,5-HAT) or related intramolecular shifts through 6–8-membered transition states, which are both kinetically and thermodynamically preferred under mild conditions (10.1007/s11426-024-2252-9). This bias favors C_4–C_6 fragmentation, with succinic acid (C4) being the dominant product, while formation of longer diacids requires higher-energy, less favorable pathways.

In summary, the selective formation of short diacids, particularly C_4, is an intrinsic consequence of low-temperature •OH-mediated radical oxidation within a confined interfacial environment, fundamentally distinct from the unselective fragmentation of high-temperature thermal pyrolysis.

Comment 5. *The authors investigated PE degradation under varied conditions such as temperature, O_2 pressure, and water volume. However, throughout all experiments, the stirring rate was held constant at 600 rpm. Can the authors comment on whether altering the stirring rate might influence microdroplet formation and, consequently, affect reaction rates or product selectivity? Investigating this parameter could be valuable, as it may reveal more optimal operating conditions.*

Response: We investigated the effect of stirring rate on the reaction (300, 600, and 900

rpm). We found that at 300 rpm both the conversion and product yield decrease markedly, whereas at 600 and 900 rpm the conversion and selectivity are comparable. Therefore, a stirring rate of 600 rpm was selected as the standard condition. The relevant data are shown in Fig. S19.

The specific revisions are outlined below:

Page 28 in the revised Supplementary Information:

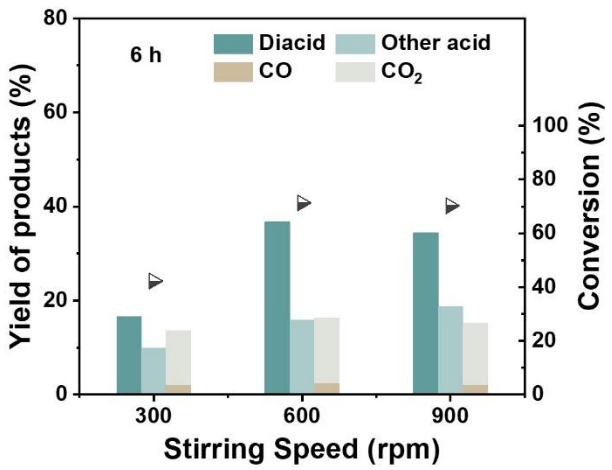

Fig. S19. Product yields and PE conversion as a function of stirring rate at 125 °C and 2 MPa O_2 after 6 h.

At intermediate stirring (600 rpm), an optimal balance is achieved: droplets form efficiently while maintaining sufficient interfacial residence time, resulting in the highest overall conversion and diacid selectivity. At very low stirring rates, limited droplet formation and fragment generation occur, leading to lower PE conversion and diacid yield. At very high stirring (900 rpm), although dispersion is vigorous, convective stripping removes partially oxidized monoacids and oligomers from the droplet surface into the bulk water before complete oxidation, reducing diacid selectivity and shifting the product distribution toward partially oxidized species.

Comment 6. *As the authors rightly mention, additives pose a major challenge in the catalytic conversion of plastic waste. Although the authors demonstrate the robustness of their process in the presence of certain metal salts, a key class of additives present in most consumer-grade plastics are antioxidants (both primary and secondary). These additives inhibit oxidation either by neutralizing free radicals or decomposing*

peroxides, thereby preventing degradation. Similarly, hindered amine light stabilizers act as radical scavengers to suppress photooxidation. Recent studies (Nat. Chem. Eng. 2025, 2, 220-228; J. Am. Chem. Soc. 2025, 147, 11227-11238) have shown that such additives can significantly influence catalytic plastic conversion. Although the authors demonstrate applicability to consumer-grade PE, the type and concentration of additives vary widely among products. Despite the process being non-catalytic, it depends on radical chemistry, making it especially important to understand how these additives affect reaction efficiency and product selectivity. Therefore, I recommend that the authors perform additional experiments to evaluate the influence of these additives on process performance and product outcomes.

Response: We thank the reviewer for highlighting this important point. Antioxidants (both primary and secondary) and hindered amine light stabilizers (HALS) are key additives in commercial plastics, and their radical-scavenging properties could potentially affect •OH-mediated oxidation in our system.

To address this, we performed experiments introducing representative stabilizers at loadings corresponding to typical levels in consumer plastics (0.5 wt% BHT, 0.5 wt% bis(2,2,6,6-tetramethyl-4-piperidyl)sebacate (HALS), 0.4 wt% Irganox 1010, and 1 wt% 6PPD), and at twice those concentrations. Under these conditions, PE conversion and diacid yields remained essentially unchanged (Fig. S30), indicating that the interfacial •OH pathway is robust to common radical-scavenging additives.

Consistently, a variety of commercial plastics were efficiently converted under identical conditions (Fig. 4A). Notably, agricultural mulch film—a polyolefin containing HALS—was fully converted at 125 °C and 2 MPa O_2 in 12 h, yielding 53 wt% diacids, comparable to laboratory-grade PE (Fig. S30). These results demonstrate compatibility with real-world polyolefin waste, and the corresponding data and discussion have been added to the revised Supplementary Information.

The specific revisions are outlined below:

Page 9, line 20 in the revised manuscript:

We additionally evaluated the effects of excess antioxidants, hindered amine light

stabilizers (HALS), and representative inorganic impurities commonly found in commercial plastics (Fig. S30-31). Under typical formulation loadings, these additives had no detectable impact on either the reaction conversion or diacid yield, underscoring the robustness of the process.

Page 38 in the revised Supplementary Information:

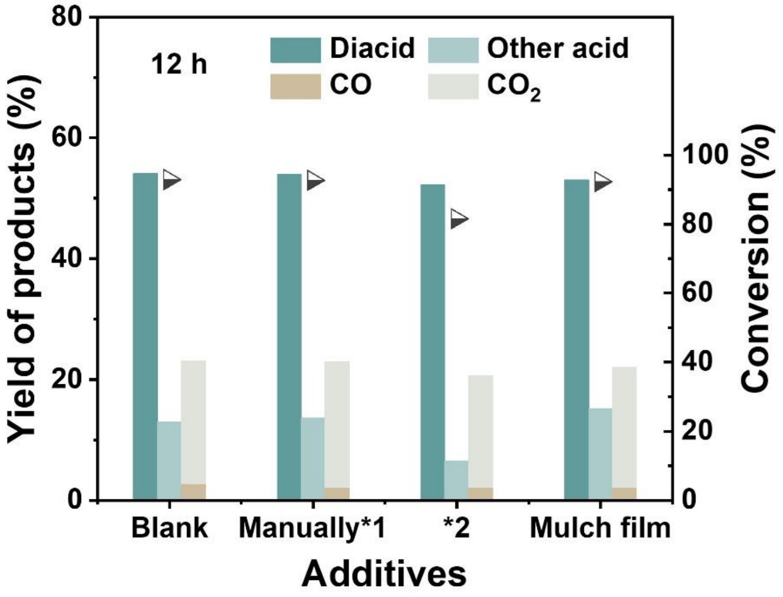

Fig. S30. The yield of products and PE conversion under different conditions of additives. (Manually add*1: 0.5 wt% BHT (a phenolic antioxidant), 0.5 wt% Bis(2,2,6,6-tetramethyl-4-piperidyl)sebacate (HALS), 0.4 wt% Irganox 1010 (Pentaerythritol tetrakis[3-(3',5'-di-tert-butyl-4'-hydroxyphenyl)propionate]) and 1 wt% N-(1,3-dimethylbutyl)-N'-phenyl-p-phenylenediamine (6PPD); *2: twice the manually added amount of * 1; Mulch film: agricultural film containing HALS)

Comment 7. *Many consumer-grade plastics also contain inorganic additives (fillers) such as TiO2, CaCO3, and ZnO, which can influence process performance (https://doi.org/10.26434/chemrxiv-2025-0nfrn). Do the authors anticipate that these fillers could affect their oxidative upcycling process?*

Response: We thank the reviewer for raising this important point. To directly evaluate the influence of inorganic fillers commonly present in consumer-grade plastics, we conducted experiments using with both representative and intentionally exaggerated impurity levels. At realistic (5 wt% TiO_2 + 5 wt% $CaCO_3$ + 0.1 wt% ZnO), no

measurable change in PE conversion or diacid yield were observed. Importantly, several real-world plastics examined in this work-such as the white bottle cap shown in Fig. 4A and a commercial PE pipe (Fig. S31)-are representative TiO_2-containing consumer products, yet their conversion proceeded without detectable performance loss.

The specific revisions are outlined below:

Page 9, line 20 in the revised manuscript:

We additionally evaluated the effects of excess antioxidants, hindered amine light stabilizers (HALS), and representative inorganic impurities commonly found in commercial plastics (Fig. S30-31). Under typical formulation loadings, these additives had no detectable impact on either the reaction conversion or diacid yield, underscoring the robustness of the process.

Page 39 in the revised Supplementary Information:

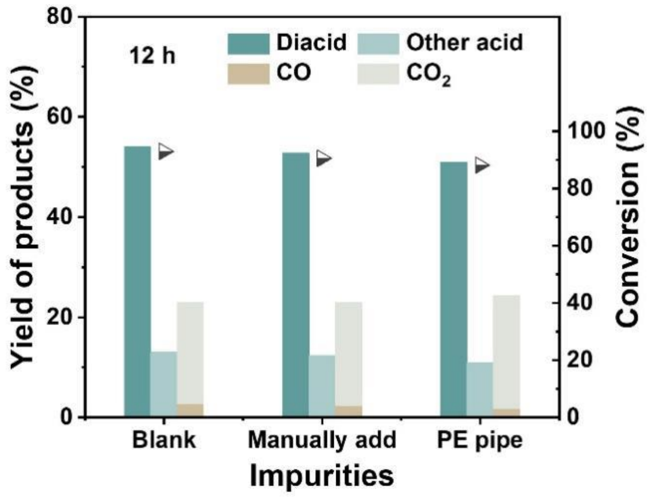

Fig. S31. The yield of products and PE conversion under different concentrations of inorganic impurities. (Manually add: 5 wt% TiO_2 + 5 wt% $CaCO_3$ + 0.1 wt% ZnO; PE pipe: containing TiO_2)

Comment 8. *The authors report that benzoquinone quenches superoxide radicals (•O2-), thereby suppressing PE conversion (Fig. S12). A related concern arises for rubber feedstocks, which commonly contain N-(1,3-dimethylbutyl)-N'-phenyl-p-phenylenediamine (6PPD), a widely used antioxidant and antiozonant. Upon oxidation,*

6PPD forms 6PPD-quinone, a compound known for its aquatic toxicity (Science 2021, 371, 185–189; Nat. Chem. Eng. 2024, 1, 597–607). Can the authors comment on whether 6PPD or its oxidation product, 6PPD-quinone, could interfere with or influence their oxidative upcycling process?

Response: We thank the reviewer for this insightful and detailed question. As discussed in *Comment 8*, we have systematically examined the influence of common organic additives, including antioxidants and antiozonants such as 6PPD, on the oxidative upcycling process. At loadings representative of commercial formulations, we observed no significant changes in polymer conversion or product selectivity (Fig. S30).

High-resolution LC-MS analysis of the post-reaction solution confirmed the presence of 6PPD-quinone (Fig. R5a), indicating that 6PPD is readily oxidized under the reaction conditions and therefore does not persist as an effective radical inhibitor at realistic concentration in our system. Our previous quenching experiment with benzoquinone (Fig. S12) also showed that only higher concentrations would significantly inhibit the reaction.

To further assess rubber-specific relevance, we deliberately introduced additional 6PPD into rubber feedstocks at varying levels. When the added amount was within 5 wt%, the impact on conversion and diacid yield remained negligible. A noticeable suppression of performance was only observed at unrealistically high loadings, well above typical industrial additive levels (Fig. R5b). For aqueous effluents, mature PAC/GAC adsorption units can effectively remove 6PPD and 6PPD-Q, followed by established advanced oxidation processes (e.g., UV-activated ozonation or Fenton oxidation) to mineralize residual 6PPD-Q and mitigate associated toxicity.

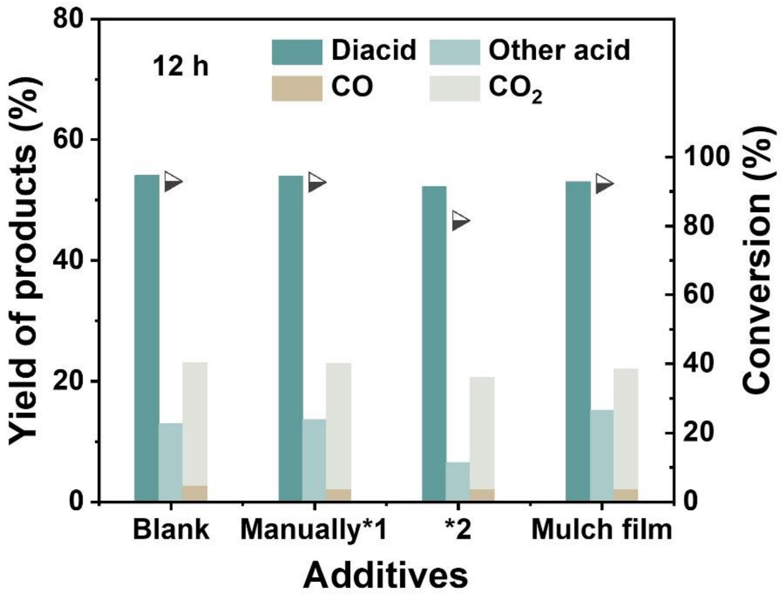

Fig. S30. The yield of products and PE conversion under different conditions of additives. (Manually add: 0.5 wt% BHT (a phenolic antioxidant), 0.5 wt% Bis(2,2,6,6-tetramethyl-4-piperidyl)sebacate (HALS), 0.4 wt% Irganox 1010 (Pentaerythritol tetrakis[3-(3',5'-di-tert-butyl-4'-hydroxyphenyl)propionate]) and 1 wt% N-(1,3-dimethylbutyl)-N'-phenyl-p-phenylenediamine (6PPD); *2: twice the manually added amount of * 1; Mulch film: agricultural film containing HALS)

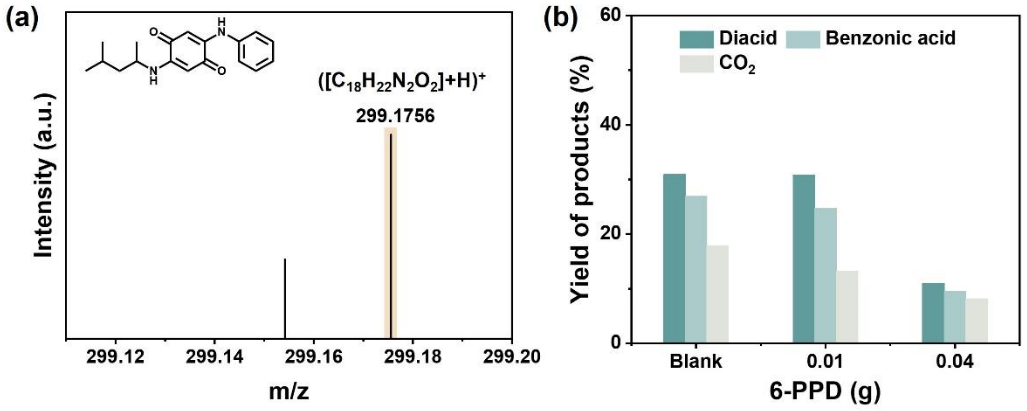

Fig. R5. (a) The detection of 6PPD-Q after 12 h by high-resolution LC-MS. (b) The yield of products and waste tires conversion under different amounts of 6-PPD.

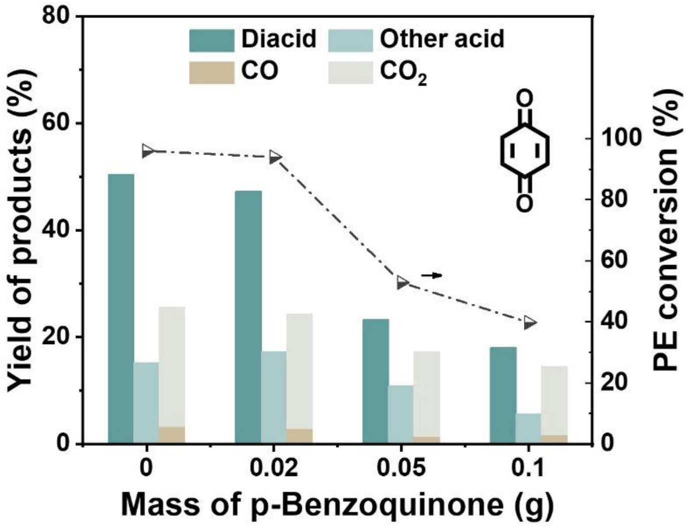

Fig. S12. The yield of products and PE conversion under different amounts of benzoquinone. Polyethylene (0.2 g), 5 mL DI water and benzoquinone (0, 0.02, 0.05 or 0.1 g) were loaded into the autoclave.

Comment 9. *Real-world plastic waste streams are highly heterogeneous and typically contain polymers such as PET, PVC, PU, and others in addition to polyolefins. These materials are known to influence catalytic processes by generating undesirable byproducts (e.g., HCl from PVC decomposition; J. Am. Chem. Soc. 2025, 147, 11227 – 11238; Nat. Chem. Eng. 2024, 1, 376-384). Similarly, hydrolysable polymers such as PET and nylon can yield acids, amines, or alcohols that may interfere with the reaction. Although the present process is non-catalytic, such byproducts could still impact reaction pathways or product selectivity. Therefore, I strongly recommend that the authors perform experiments using heterogeneous mixed waste streams to evaluate the robustness and general applicability of their process.*

Response: We thank the reviewer for this important comment. We fully agree that real-world plastic waste streams are heterogeneous and that non-polyolefin fractions can affect processing and product outcome. We believe that when recycling mixed plastics in reality, preliminary separation should be carried out first (such as using density separation method or spectral sorting machine).

In laboratory, mixed waste streams are commonly pre-sorted: polyolefins (PE/PP/PS) typically float in water while PET, PVC, nylon and many other heteroatom-containing

polymers sink. As shown in Fig. R6 (and discussed in the SI), simple density-based separation provides an effective and low-cost first step to remove high-chlorine and high-density fractions prior to oxidative treatment. For these reasons we do not recommend processing unsorted, heterogeneous plastic mixes together in our oxidative system--particularly mixtures containing PVC.

However, this does not imply that our system is intrinsically incapable of handling mixed plastics. As discussed in *Comment 11*, we have explored representative mixed-plastic feeds and identified conditions under which polyolefin conversion can still proceed. Nevertheless, for practical deployment of the present oxidative upcycling strategy, prior removal of PVC is essential, and targeted separation of hydrolysable polymers is strongly advisable to maximize efficiency, product selectivity, and reactor longevity.

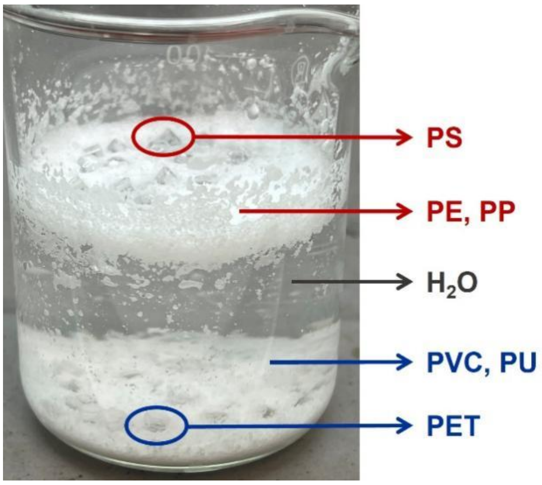

Fig. R6. Schematic diagram of separating common mixed plastics using density method (with water).

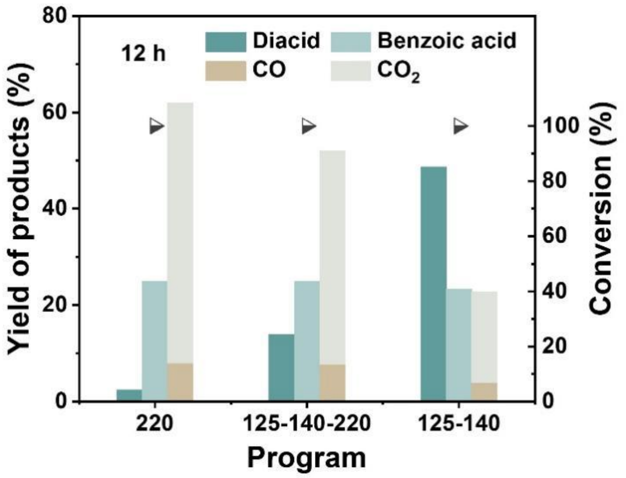

Fig. R3. The product yields from mixed plastics under different heating protocols.

When density-based separation becomes ineffective due to the presence of inorganic fillers in real plastic waste, more advanced sensor-based sorting technologies can be employed. Commercial equipment such as Autosort™ FLAKE, which integrate near-infrared (NIR) spectroscopy, high-resolution imaging, metal detection, and AI-assisted classification, are capable of discriminating and automatically separating PET, PVC, PP, PE, PS, PA and other common polymer types (including polymer alloys). Such optical sorting technologies are already deployed in waste plastics recovery facilities.

Comment 10. *Likewise, packaging waste often consists of multilayer films made from coextruded polymers. Typical compositions include layers of PE, PET, EVOH, EVA, and nylon. It would be valuable for the authors to demonstrate the applicability of their process to such multilayer packaging waste, given its prevalence and complexity in real-world plastic streams.*

Response: We thank the reviewer for this important and constructive suggestion. In response, we carried out a preliminary test using a representative multilayer food-packaging film (Fig. R2), which typically consists of coextruded polymer layers together with a thin metallized barrier layer. The packaging material was simply rinsed, cut into small pieces, and directly subjected to the oxidative process without any prior delamination or separation.

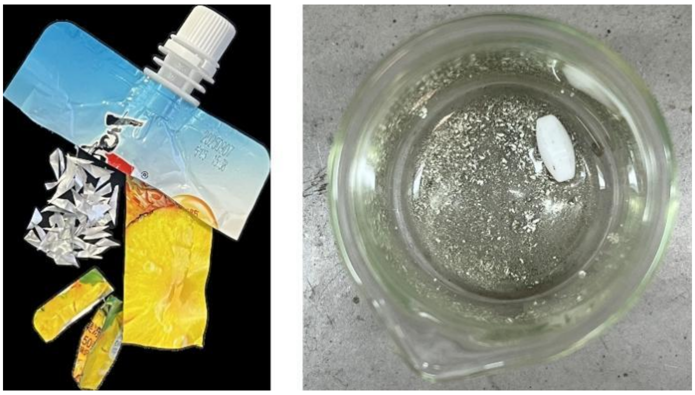

Fig. R2. The photo of the packaging waste and post-reaction solution.

After reaction at 220 °C for 12 h, a clear, faintly yellow aqueous phase was obtained, accompanied by a small amount of finely particulate solid residue exhibiting metallic luster (Fig. R2). XRD analysis of this solid residue confirmed that it is predominantly Al (Fig. R7), originating from the metallized barrier layer commonly employed in food packaging films. Analysis of the liquid-phase products showed that diacids remained the dominant products, accounting for 45.4% of the carbon-containing products, while CO and CO_2 together contributed 21.4% (Fig. R8). The remaining 24.5% corresponds to undetected residues, likely associated with metallized barrier layer or interfacial losses.

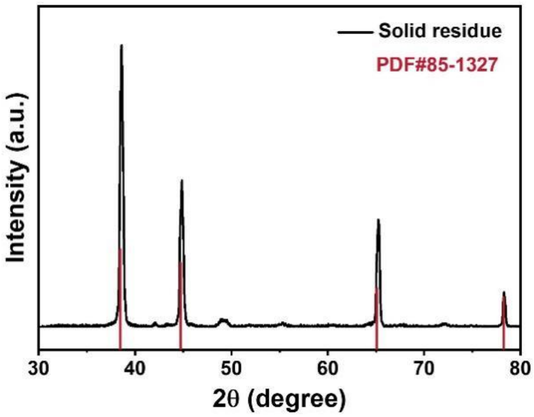

Fig. R7. The XRD pattern of the solid residue.

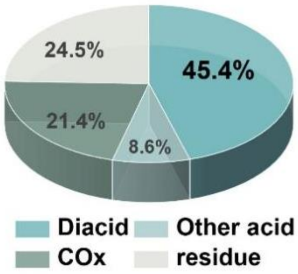

Fig. R8. The product yields from packaging waste conversion.

Overall, these results demonstrate that the present catalyst-free interfacial oxidation strategy can tolerate realistic multilayer packaging waste and selectively convert the polyolefin-rich components into diacids, even in the presence of barrier layers and heterogeneous polymer compositions.

Comment 11. *An added challenge with mixed plastic waste is that different polymers have varying melting temperatures. Identifying a single optimal reaction temperature suitable for the entire mixture may be difficult, as conditions that work for one polymer may cause overoxidation of others, potentially leading to higher* CO_x *formation. While the authors report that "further oxidation of short-chain diacids was minimal, as confirmed by control experiments using commercial diacids (Fig. S24)," this behavior is clearly temperature-dependent. When processing mixed wastes, higher temperatures may be required to accommodate polymers with higher melting points, which could increase CO2 generation. Furthermore, the authors should discuss how individual carboxylic acids are separated from the complex product mixture, as mixed feedstocks may yield a wide range of products from different polymers.*

Response: We thank the reviewer for highlighting the important issue of variable melting points in mixed waste streams and the associated risk of overoxidation when higher temperatures are required.

First of all, we believe that the best solution is to perform a preliminary separation before oxidation to achieve more efficient and selective recycling, as mentioned in *Comment 9*. Nonetheless, we carried out co-processing experiments using representative polymer mixtures and systematically screened different temperature profiles. Direct operation at 220 °C led to extensive CO_2 formation, with low diacid

yields. A stepped heating protocol (125 °C 3 h, 140 °C 3 h, followed by 220 °C 6 h) similarly increased CO_2 formation, despite a higher diacid yield. In contrast, a milder two-stage protocol (125 °C for 6 h followed by 140 °C for 6 h) provided a more favorable outcome: dicarboxylic acids accounted for ~48.7% of the products, while CO_2 formation was substantially suppressed and benzoic acid yield remained comparable (Fig. R3).

In mixed polyolefin blends, the low-melting component (PE) softens and melts first and acts as a solvent phase into which higher-melting polymers (PP, PS) dissolve. This behavior enables interfacial •OH-mediated oxidation to proceed at temperatures below the nominal melting points of the higher-melting components. Although these results demonstrate the feasibility of our approach for mixed plastics, we nevertheless consider physical separation using mature sorting technologies followed by polymer-specific conversion to be a more rational and controllable strategy for practical implementation.

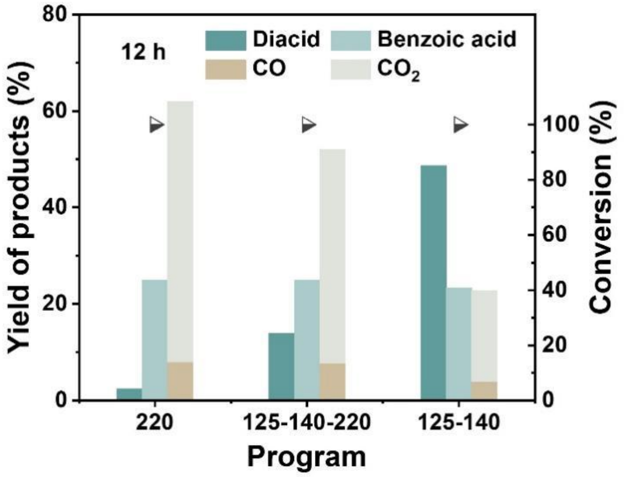

Fig. R3. The product yields from mixed plastics under different heating protocols.

At the laboratory scale, we have demonstrated the separation of mixed dicarboxylic acids by a combination of extraction, adsorption, and temperature-controlled fractional crystallization, as detailed in the Supplementary Information. At the industrial level, the mixed diacids can be processed to isolate C_4-C_5 diacids via established dehydration-distillation routes through their corresponding anhydrides. Higher homologues (C_6-C_8), which are formed in smaller quantities, do not require further fractionation and can be directly utilized as mixed diacid streams for polymer synthesis or surface treatment,

where strict carbon-number purity is not required (Fig. S33). As for mixed polyolefins upcycling, the mixed acid streams can be efficiently separated through initial water removal and preferential recovery of volatile acetic acid. Benzoic acid can be selectively separated from aliphatic diacids by ethyl acetate extraction, exploiting its higher solubility in moderately polar organic solvents, and subsequently recovered by crystallization after solvent removal.

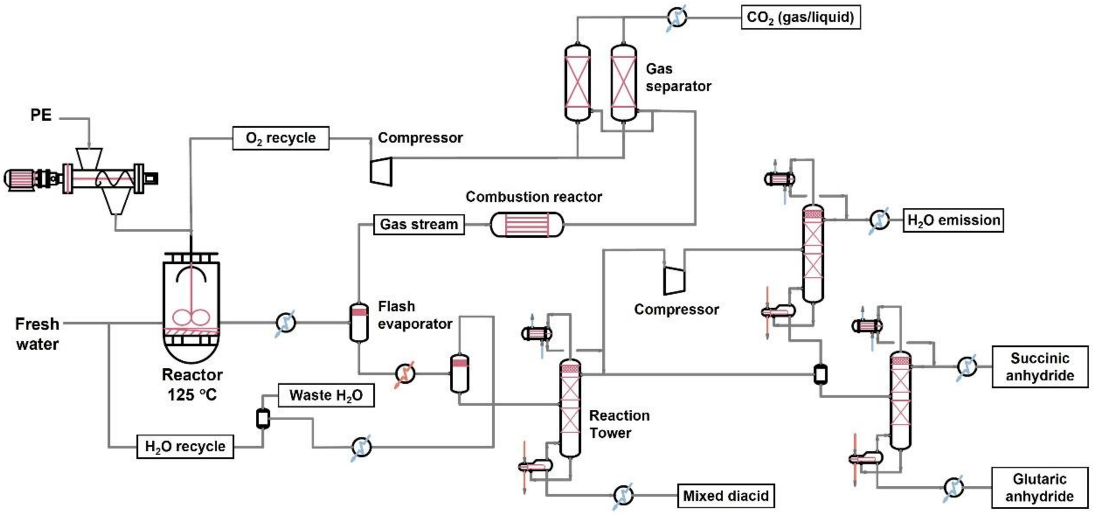

Fig. S33. Proposed process flow diagram to produce diacid from waste PE via catalyst-free oxidation.

Comment 12. *The authors state that "real tires processed at 180 °C produced 24.4% inorganic solids and 57.9% liquid products". The composition of these liquid products should be clearly specified. Based on the statement, it is unclear fraction comprises diacids versus monoacids or other species?*

Response: We apologize for the lack of clarity in the original description. The composition of the liquid products obtained from real tire processing has now been explicitly quantified. Within the liquid fraction, the yield of diacids is 30.9%, with succinic acid as the dominant diacid, while benzoic acid is the major monocarboxylic product (26.9%). We have revised the manuscript to clearly specify the distribution of the liquid products.

The specific revisions are outlined below:

Page 10, line 11 in the revised manuscript:

The method also degraded challenging rubber-based materials. Tire waste-polybutadiene processed at 160 °C yielded predominantly succinic acid, while real tires processed at 180 °C produced 33.9% diacid and 26.9% benzoic acid, along with 24.4% inorganic solids, despite the presence of vulcanizers that would severely poison conventional metal or acid catalysts.

Comment 13. *The authors state that "scalability was confirmed in a 5 L reactor (300 g PE, 3 L H_2O, 2 MPa O2) achieving 89% conversion with saturated diacid yields over 52% after 48 h." However, the reaction time is nearly four times longer than in the small-scale reactor. Can the authors explain the cause of this slower reactivity?*

Response: We thank the reviewer for this important observation. The longer reaction time observed at the 5 L scale is primarily due to physical and engineering factors rather than changes in the underlying chemistry. In larger vessels, gas-liquid mass transfer and local mixing uniformity are inherently less efficient, limiting the rate of oxidative cleavage. These scale-dependent effects are well recognized in multiphase reactor engineering and motivate the use of targeted intensification strategies at higher throughputs. In ongoing work, we are addressing these limitations through deliberate hydrodynamic and reactor-design improvements. Specifically, we have designed a plug-flow reactor configuration (Fig. R1) that enables continuous feeding of molten polymers, inline high-shear dispersion, and spatially controlled oxidation along the reactor length. This design minimizes back-mixing, stabilizes microdroplet formation, and allows precise control over residence time and oxidation extent. In parallel, enhanced gas–liquid contacting strategies—including Venturi-assisted mixing—are incorporated to intensify shear and turbulence, continuously renew oil–water and gas–liquid interfaces, and maintain a high steady-state interfacial area. Such designs are expected to restore high interfacial area and substantially accelerate reaction rates at scale.

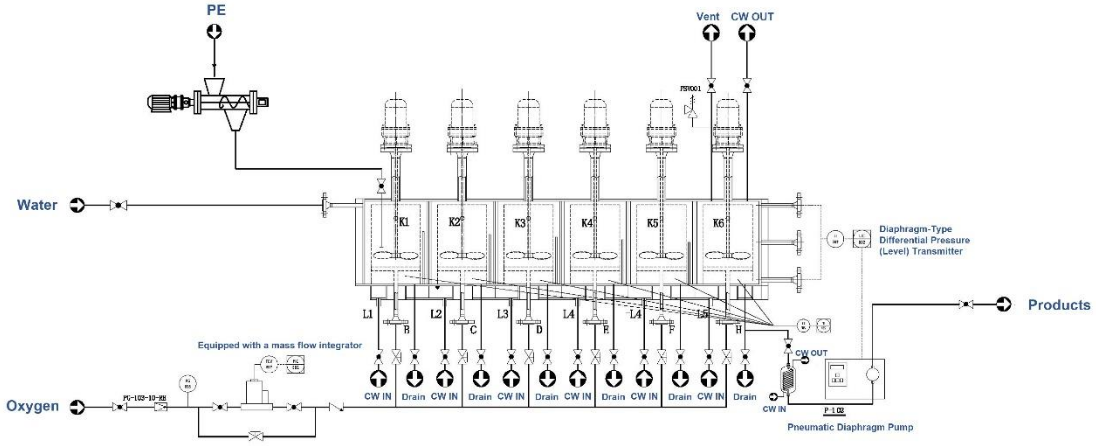

Fig. R1. Process flow diagram of the plug-flow reactor designed for continuous microdroplet oxidation of polymers.

This reactor enables the oxidative upcycling of polyolefins in a spatially and temporally controlled manner. Polymers are continuously fed via a heated screw and feeder, where mechanical agitation disperses the polymer into the aqueous phase, forming polymer－water interfacial microdomains. Oxygen is supplied through a mass-flow-controlled gas line along the reactor length, ensuring sufficient oxidant availability while minimizing local over-oxidation. As the reaction mixture progresses, gradual chain scission and oxygen incorporation produce oxygenated oligomers and ultimately short-chain carboxylic acids. This design allows continuous operation with controlled reaction extent, maintaining the diacid selectivity observed in batch experiments and providing a clear route toward scalable implementation.

Comment 14. *Fig. S20- FTIR Analysis: The post-reaction polymer spectrum shows additional features between 1000-1500 cm-1 besides the carbonyl peak. Please specify clearly which functional groups these correspond to. Furthermore, it may be useful to supplement FTIR results with high-temperature 1H-NMR to confirm bulk oxidation of PE and quantify different functional groups.*

Response: We thank the reviewer for this helpful suggestion. The additional FTIR features observed in the post-reaction polymer spectrum (1000-1500 cm^{-1}) can be assigned as follows and correlate well with our ^{1}H-NMR observations:

1. The broad band near ~3200 cm^{-1} is assigned to O–H stretching vibrations.

2. The absorption at ~1465 cm^{-1} corresponds to the CH_2 scissoring mode of the polyethylene backbone. The markedly reduced intensity of this band in the 3 h product indicates backbone degradation and is consistent with the diminished aliphatic signals at δ = 0.77-1.56 ppm in the ^{1}H-NMR spectrum.

3. Peaks at ~1375 and 1355 cm^{-1} are attributed to symmetric deformation modes of terminal and short-chain alkyl methyl groups, reflecting increased chain-end formation resulting from oxidative β-scission.

4. Bands in the 1300–1200 cm^{-1} region are assigned to C-O related vibrations and CH_2 deformation modes associated with oxidized polyethylene, particularly methylene groups adjacent to carbonyl functionalities and hydrogen-bonded carboxylic acid motifs.

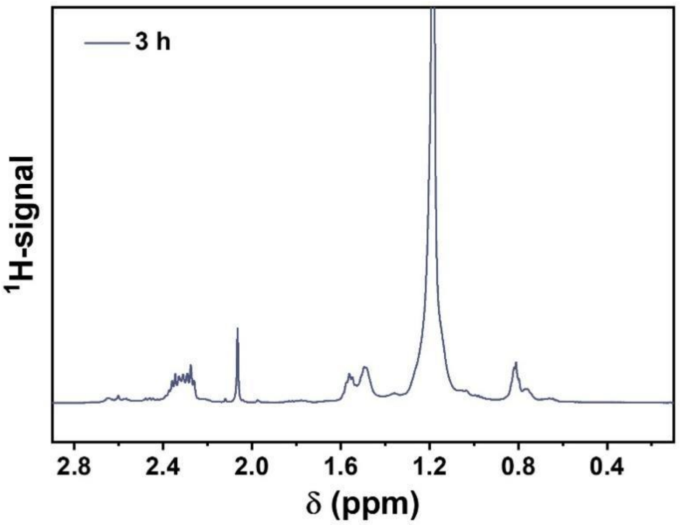

Fig. R9. ^{1}H-NMR spectrum of the solid residue after 3 h.

The specific revisions are outlined below:

Page 31 in the revised Supplementary Information:

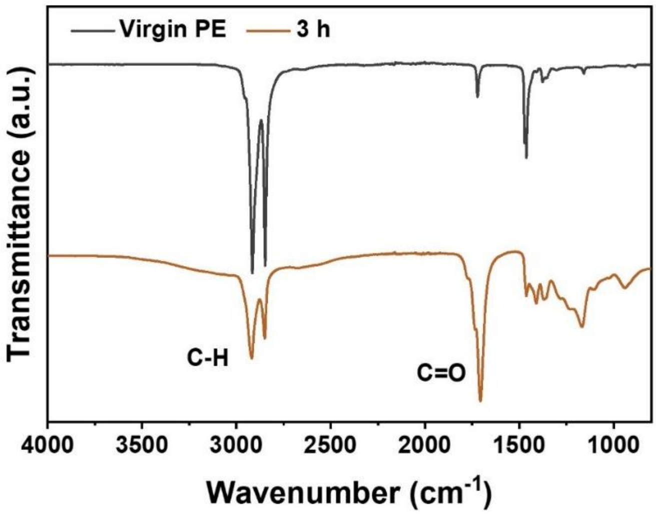

Fig. S22. The IR spectrum of virgin PE and the solid residue after 3 h. The oxidized polymer shows emerging carbonyl bands and weakened C–H stretching features, confirming the introduction of oxygenated functionalities along the polymer backbone. New absorptions appear in the 1000–1500 cm^{-1} region, accompanied by attenuation of the CH_2 scissoring band at ~1465 cm^{-1}, indicative of backbone oxidation and chain scission. Bands at ~1375 and 1355 cm^{-1} are assigned to terminal alkyl methyl deformations generated by oxidative β-scission, while features at ~1300–1200 cm^{-1} arise from C–O-related vibrations and CH_2 deformation modes in oxidized polyethylene. These assignments are consistent with the accompanying 1H NMR results.

Comment 15. *Given that the authors show that •OH radicals are generated even under N_2, some oxidation of unconverted PE would be expected. Do the authors observe any hydroxyl, carbonyl, or other oxygen-containing functionalities on the residual solids?*

Response: We thank the reviewer for this insightful question. Under a N_2 atmosphere, after 12 h of reaction the residual PE indeed exhibited a slight yellowing (Fig. R10), suggesting that only very limited chemical modification had occurred. However, FTIR analysis of the recovered solid shows no discernible new absorptions attributable to hydroxyl, carbonyl, or other oxygen-containing functional groups when compared with virgin PE (Fig. R11). These results indicates that, although trace •OH radicals can be transiently generated even under N_2, their concentration and lifetime are insufficient to

sustain deep oxidation or oxidative chain scission of PE in absence of O_2. Together, these observations demonstrate that molecular oxygen is essential for maintaining continuous radical generation and for driving effective oxidation and depolymerization of PE in this system (as mentioned in the manuscript Fig. 3A).

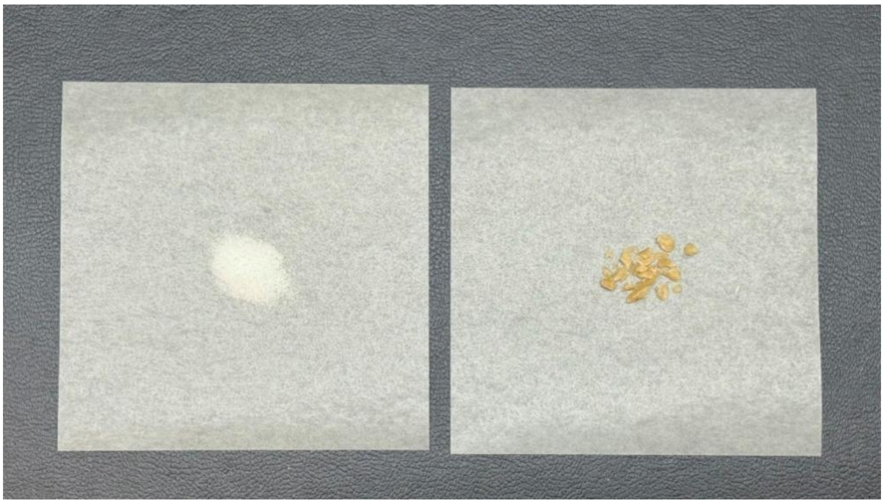

Fig. R10. The comparison of virgin PE and residual PE after 12 h under N_2.

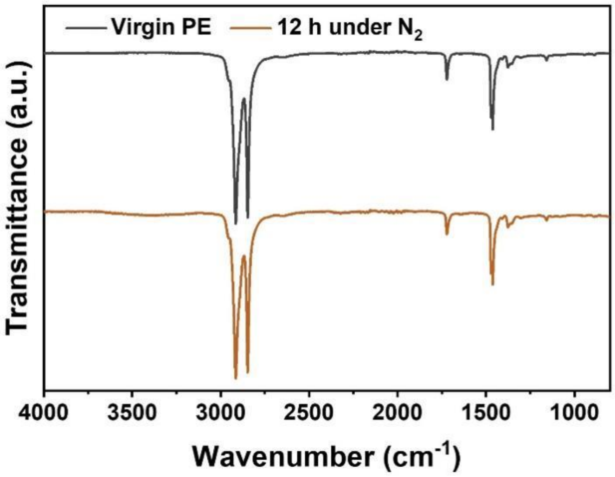

Fig. R11. The IR spectrum of virgin PE and solid residue after 12 h under N_2.

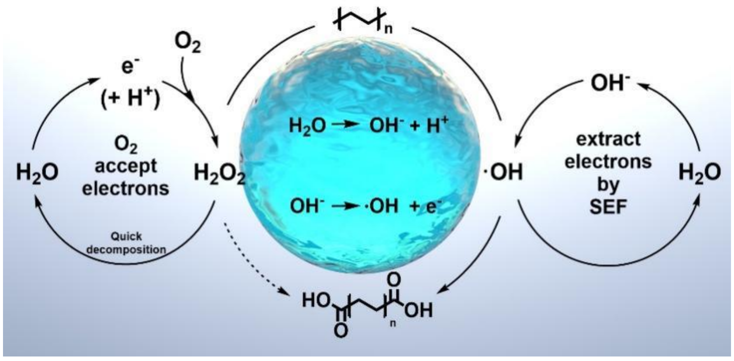

Fig. 3A. Mechanism diagram of the spontaneous oxidation of PE to diacid on the surfaces of microdroplets.

Comment 16. *Figure 2D: Including EPR spectra for the PE + H2O + O2 system in Fig. 2D would help compare the relative amounts of •OH radicals formed under different conditions.*

Response: We thank the reviewer for this suggestion. We have added the EPR spectra for the PE + H_2O system under O_2 and N_2 atmospheres to Fig. 2D. The DMPO spin-adduct signal is markedly stronger under O_2, consistent with substantially enhanced •OH generation in the presence of molecular oxygen.

The specific revisions are outlined below:

Page 8, line 6 in revised the manuscript:

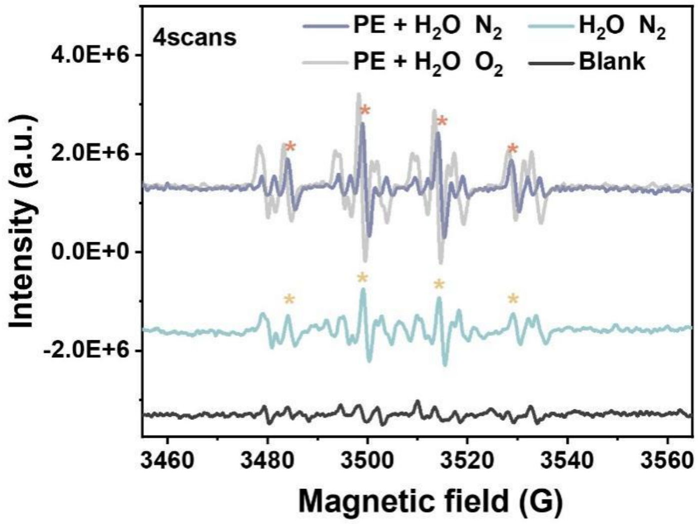

(D) EPR spectra of the system under 2 MPa N_2 and 2 MPa O_2, recorded with and without PE.

Comment 17. *The authors must provide details on the calibration standards used and the molecular weight calibration range for the HT-GPC in the methods section. If the system was not properly calibrated, or if the product MW falls outside this range, the reported MW values may be inaccurate.*

Response: We appreciate the reviewer's concern. HT-GPC measurements were calibrated using narrow polystyrene standards, and molecular weights are therefore

reported as PS-equivalent values, which is a common practice for polyolefins at high temperature. Although the measured Mw of the 3 h residue (~455 Da) is slightly below the nominal lower calibration limit (~500 Da), the deviation is small and the result clearly indicates a transition from polymeric PE (Mw=4700 Da) to oligomeric fragments. Note that the GPC data are used here to demonstrate extensive chain scission rather than to assign an exact absolute molecular weight, and this conclusion is fully consistent with FTIR and liquid-phase product analyses.

The specific revisions are outlined below:

Page 2 in the revised Supplementary Information:

High-temperature gel permeation chromatography (HT-GPC) measurements were calibrated using narrow polystyrene (PS) standards. The effective calibration range of the system spans approximately 500-1*10^7 g mol^{-1}.

Comment 18. *The authors state, "High-MW PE waste including gloves, LDPE bags, HDPE caps…". However, these materials cannot be classified as high-MW PE without reporting their molecular weights. I suggest referring to them as consumer-grade PE instead.*

Response: To avoid ambiguity and ensure accurate description, we have revised the manuscript to refer to these materials as consumer-grade polyethylene (PE) (e.g., gloves, LDPE bags, HDPE caps) rather than high-MW PE.

The specific revisions are outlined below:

Page 9, line 18 in the revised manuscript:

Consumer-grade PE waste including gloves, LDPE bags, HDPE caps…

Comment 19. *Introduction, Lines 54 – 59: The authors state, "However, accumulation of miscellaneous additives and contaminants inevitably lead to catalyst deactivation, …." Please provide supporting citations for this claim.*

Response: In accordance with the reviewer's suggestion, we have added the following relevant references:

27. Ngu, J. et al. Catalytic deconstruction of organic additive-containing plastics. *Nat.*

Chem. Eng. 2, 220-228 (2025).

28. Selvam, E. et al. Conversion of compositionally diverse plastic waste over earth-abundant sulfides. *J. Am. Chem. Soc.* 147, 11227-11238 (2025).

Comment 20. *Techno-Economic Analysis: It is unclear how the assumed processing capacity of 200 kt/year was determined for the TEA. This value appears overambitious relative to current industry metrics. Presently, pilot- and commercial-scale plastic depolymerization plants typically operate in the 3 –100 kt/year range, with most reports citing capacities below 60 kt year⁻¹ (Energy Environ. Sci. 2023, 16, 3638–3653). Moreover, the achievable scale will depend strongly on the waste-processing capacity of regional materials recovery facilities. The authors are therefore encouraged to adopt more realistic and modest assumptions for the TEA to ensure a credible and representative techno-economic evaluation. Detailed explanations must be provided to justify the assumptions underlying the TEA model. A sensitivity analysis should be performed to identify the key cost drivers and parameters influencing process economics.*

Response: We thank the reviewer for this important comment. We agree that the choice of plant capacity strongly affects techno-economic conclusions and that industry context must be clearly justified. In response we have recalculated the TEA using a conservative commercial case of 60,000 t yr^{-1} and provided full documentation of the underlying assumptions in the Supplementary Information. For transparency we also added one-way sensitivity analyses (tornado charts for the five most influential parameters) and report the key financial metrics using a 15-year project horizon.

Based on a reasonable estimate of 20-40 kilograms of polyethylene waste per capita, a facility with a processing capacity of 60000 tons per year can theoretically serve approximately 1.5 to 3 million residents producing polyethylene waste. As mentioned above, a single high-throughput optical sorter (e.g., Autosort™ FLAKE) is typically rated at ~5–8 t h^{-1}. Accordingly, one such commercial sorting line or two units operated under conservative uptime assumptions, can realistically supply a 60 kt·yr^{-1} upcycling facility.

The plant breakeven point is ≈7.8 kt yr^{-1}, demonstrating economic viability well below the nominal capacity and indicating resilience to feedstock volatility and staged deployment. Our sensitivity analysis shows that product price and total capital investment are the dominant drivers of NPV, while reaction residence time also strongly influences NPV because it controls reactor volume (Fig. S34). These results support the characterization of the process as capital-intensive with economics that depend on high product value and efficient reactor design.

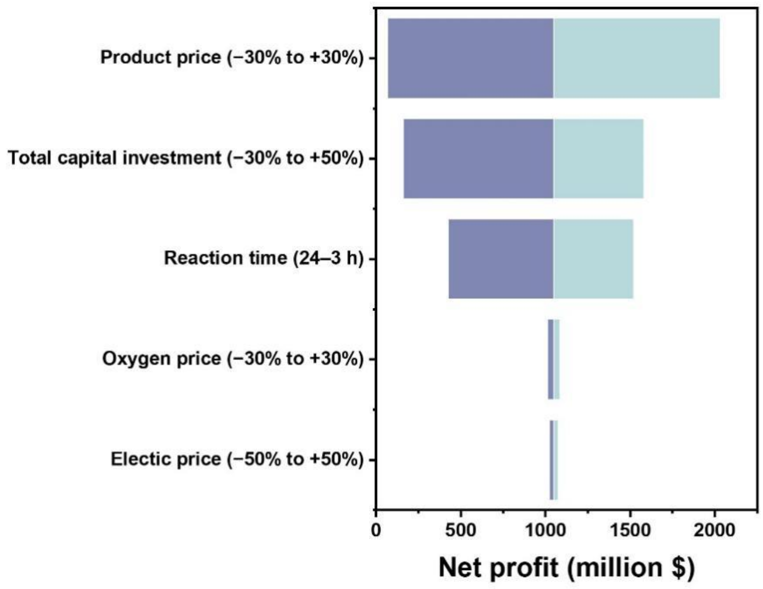

Fig. S34. One-way sensitivity analysis of net present value (NPV).

The specific revisions are outlined below:

Page 11, line 2 in the revised manuscript:

Techno-economic analysis (TEA) confirmed the robust economic viability of the catalyst-free polyolefin degradation process (Fig. S27). At plant scale the effluent is cooled and flashed to separate a gas stream (O_2, CO_2 and CO) and a liquid phase; CO is combusted for energy recovery and the flue gas is subjected to CO_2 capture while process water is recovered and recycled. The heavier liquid fraction is upgraded via dehydration/distillation to convert C_4–C_5 diacids to their anhydrides, followed by fractional distillation to obtain purified products. Evaluated at 60 kt yr^{-1}, the design requires a total capital investment of $141 million (Tables S6–S10) and yields an annual net profit of $74.0 million after tax, giving a payback of ~3.1 years without government

subsidies (Fig. 5D). The breakeven point is ~7.8 kt yr^{-1}, demonstrating sustained economic viability across an exceptionally broad spectrum of polymer waste input volumes. This remarkably low threshold signifies operational resilience to feedstock volatility and supports potential deployment at sub-nominal capacities. Our sensitivity analysis shows that product price and total capital investment are the dominant drivers of NPV (Fig. S34), while reaction residence time also strongly influences NPV because it controls reactor volume. These results support the characterization of the process as capital-intensive with economics that depend on high product value and efficient reactor design.

The specific revisions are outlined below:

Page 41 in the revised Supplementary Information:

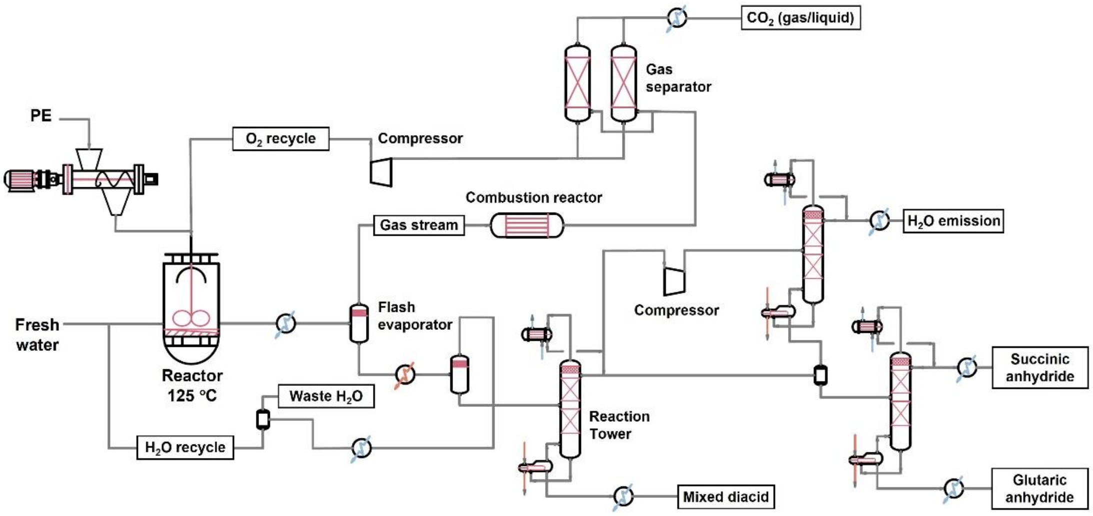

Fig. S33. Proposed process flow diagram to produce diacid from waste PE via catalyst-free oxidation.

The upcycling process operates under mild aqueous conditions without the use of precious metal catalysts or complex solvents, indicating strong potential for industrial scale-up and subsequent techno-economic assessment. The key financial metrics are reported using a 15-year project horizon.

Reactor design and throughput optimization (12 h retention time): developing continuous stirred-tank or plug-flow reactors capable of handling high solid-liquid loads, ensuring efficient mass and heat transfer at industrial scale.

Feedstock logistics: Establishing robust preprocessing systems compatible with heterogeneous commercial plastic waste streams, including advanced spectroscopic sorting (e.g., NIR sorting) for polymer identification and separation, followed by cleaning. Molten or softened polymers can then be continuously delivered using heated screw feeders or extruders, enabling stable feeding, composition control, and seamless integration with downstream oxidative upcycling units. The framework applies a 20% relative solvent reduction upon scale-up, with reactant and auxiliary stream flow rates scaled linearly [1].

Material recycling and energy recovery: Heat integration strategies were applied to reduce steam and cooling water requirements. Oxygen and water were internally recycled to minimize fresh feedstock inputs. The Calculator module in Aspen was used to ensure constant feed ratios under steady-state conditions, with water-to-PE and oxygen-to-PE mass ratios fixed at 8:1 and 3.15:1, respectively.

Product separation: After the reaction, the cooled gas–liquid effluent was subjected to a flash tank for separation. The gaseous fraction (containing CO) was combusted for safety; the resulting flue stream was then processed using an MEA-based CO_2 capture unit (assumed CO_2 capture efficiency = 50% [2]). This separation step was not explicitly modelled in Aspen but was treated using representative industrial data. The liquid fraction was flashed to separate water from higher-boiling products; The recovered water was recycled to the process, with a 1% purge stream to prevent impurity accumulation. The product stream was routed to a reactive distillation to convert succinic and glutaric acids to their corresponding anhydrides [3] The anhydrides were separated from residual mixed diacids and the two anhydride products were further purified by two fractional distillations.

A full list of TEA assumptions (product yield composition, utility consumption rates, capital expenditure breakdown, operating expenditure items, and key financial parameters) is provided in Table S6-10.

Table S6. Summary of product sales revenue (60 kton)

Product	Output (t)	Market price ($/t)	Revenue (million $)
Succinic anhydride (>99%)	43426.1	2953.2	12824.4

Glutaric anhydride (>99%)	12144.2	3482.5	4229.2
Mixed diacids (C_{6-9})	13128.1	696.5	914.4
**Total**			**17968.0**

* All TEA were calculated in RMB and converted to US dollars at the exchange rate of January 29, 2024.

Table S7. Summary of product costs (60 kton)

**Operating time h/yr**	8,000	**Capacity t/yr**		200,000
	**item**	**quantity per year (t)**	**cost per unit (USD)**	**Calculated value (10^4 USD)**
	**Raw materials**			
**1**	PE	60000	835.8/t	5014.8
**2**	O_2	63000	57.4/t	361.6
**3**	H_2O	7559.7	0.42/t	0.3
	**Utilities**			
**1**	Electricity	$1463.7*10^4$	0.10	152.9
**2**	Water	$270.1*10^4$	0.077	18.8
**3**	Low-pressure steam	$9.2*10^4$	30.65	281.4
**4**	Medium-pressure steam	$11.6*10^4$	18.39	355.0
**Total**				6184.8

* Low-pressure steam: 125 °C, Medium-pressure steam: 250 °C.

Table S8. Key assumptions about capital investment (60 kton)

	Cost(10^4 \$)	Note
**Fixed capital investments**	**8868.4**	
(1) Direct cost	*6963.8*	
Costs of major equipment	2730.8	
reactor	1316.4	
tower	438.8	
sorting and feeding	11.7	
tank& pump& pipe	117.0	
heat exchanger	146.3	
compressor	341.3	
flash evaporator	37.6	
combustion furnace	20.9	
gas separator	292.5	
mixer	8.4	
Other direct costs		(specified as % of equipment cost)
Installation	546.2	20%
Piping	546.2	20%
Instrumentation and control	546.2	20%
Building & structure	819.3	30%
Yard improvement	273.1	10%
Service facilities	1365.4	50%
Land	136.5	5%
(2) Indirect cost	*1904.8*	
Engineering & supervision	634.9	15%
Legal expenses	84.7	2%
Construction expenses	634.9	15%
Contractor's fee	127.0	3%
Contingency	423.3	10%
**Working capital**	**1330.3**	15% of fixed capital investment
**Others**	**3821.9**	
Depreciation period	10 yr	
Annual depreciation rate	0.1	
Depreciation charge	259.4	
Annual interest rate	3%	
Operating labor	618.5	10% of total product cost
Operating supervision	30.9	5% of operating labor
Utilities	927.7	15% of total product cost
Maintenance and repairs	618.5	10% of fixed capital investment
Operating supplies	185.5	30% of maintenance and repairs
Laboratory charges	6.2	20% of operating labor
Royalties	247.4	4% of TPC without depreciation
Plant overhead costs	309.2	5% of total product cost

General expenses	618.5	10% of total product cost

Table S9. Key assumptions about profits (60 kton)

	Cost(10^4 $)	Note
Variable cost	6184.8	
Salaries	618.5	10% of variable cost
Depreciation	259.4	
Maintenance and repairs	266.1	3% of fixed capital investments
Insurance	62.1	0.7% of fixed capital investments
Total cost	7390.9	
Annual profit	10577.1	
Business income tax	3173.1	30% of annual profit
Net profit	7404.0	
amortization expense	304.5	

Table S10. Summary of TEA (60 kton)

Total capital investment, million $	140.2
Fixed capital investment, million $	88.7
Raw material and power costs, million $	61.8
Total sales volume, million $	179.7
Breakeven point, kton	7.8
Net profits, million $	74.0
Investment profit rate	47%
Static payback period, yr	3.1

* All TEA were calculated in RMB and converted to US dollars at the exchange rate of January 29, 2024.

<u>Point-by-point response to reviewers' comments</u>

Title: "Catalyst-Free Valorization of Waste Plastics via Microdroplet-Interfacial Radical Scission"

Corresponding Author: Prof. Graham J. Hutchings

The reviewer's comments are shown in black; our response is shown in blue.

All revisions in the MS are highlighted in yellow.

Reviewer #1

Comments to the Author:

I have carefully reviewed the revised version of the manuscript, and I am pleased to report that the authors have addressed all previously raised concerns in a thorough and convincing manner. The revisions significantly enhance the clarity, methodological rigor, and overall contribution of the paper.

The authors have improved the exposition and provided clearer justification for their key assumptions and conclusions. The manuscript is now well structured, readable, and aligned with the standards expected for publication in a top tier journal.

In light of these substantial improvements, I recommend acceptance of the manuscript in its current form.

<u>Response</u>: We sincerely thank the reviewer for their careful re-evaluation of our revised manuscript and for the generous and encouraging assessment. We are glad that the revisions have effectively addressed the previously raised concerns and that the manuscript now meets the standards for publication. We look forward to seeing this work published.

Reviewers #2

Comments to the Author:

Firstly, we would like to thank the authors for carefully considering our previous comments and undertaking a substantial revision of the manuscript. We appreciate the effort invested in this process. After reviewing the revised manuscript and the response-

to-reviewers document, we believe that the authors have addressed most of our previous concerns. However, several issues still require clarification and, in some cases, additional justification. In particular, major concerns remain regarding the assumptions and results presented in the technoeconomic analysis and lifecycle assessment, as well as certain experimental results that require further clarification and/or additional analysis. Once these points are adequately addressed, we would be willing to recommend the manuscript for publication.

Additionally, while reviewing the revised manuscript, we came across a communications-type paper (Published: 10 February 2026) that conceptually uses a similar framework as the present study: Y. Chang, W. Gao, S. Wang, et al., "Aqua-Oxidation of Polyethylene Into Carboxylic Acids Under Mild Conditions: A Catalyst-Free Upcycling Strategy for Nonpolar Plastics," Angew. Chem. Int. Ed., (2026): e23067. We were surprised to see that this paper also originates from the same university as the authors of the present study, although there is no overlap in authorship. Despite this conceptual overlap, we believe that the current manuscript is more rigorous, provides deeper fundamental insights into the process, and demonstrates broader applicability. However, I leave it to the discretion of the Editors to determine whether the publication of the other manuscript affects the present submission.

Response: We sincerely thank the reviewer for their careful re-evaluation of our revised manuscript and for the generous and encouraging assessment. We are gratified that the revisions have effectively addressed the majority of the previously raised concerns. We especially appreciate the reviewer's recognition of the rigor, mechanistic depth, and broad applicability of our work. Regarding the remaining points concerning the technoeconomic analysis (TEA), lifecycle assessment (LCA), and specific experimental clarifications, we have provided detailed justifications and additional analyses below to ensure the manuscript meets the highest standards for publication.

Additionally, we also noted the recent publication (Angew. Chem. Int. Ed., 2026, e23067). While the two studies share certain phenomenological similarities, they differ fundamentally in their core mechanistic conclusions, interpretive framework, and scope of applicability. We are confident that our manuscript presents a distinct and independent contribution. We concur with the reviewer's assessment regarding the greater rigor and deeper mechanistic insights offered by our work, and are grateful to the Editors for the opportunity to further clarify these distinctions.

Comment 1. *Figs. S17, S18 and S20: Reaction times and corresponding polymer conversions are not clearly reported in these figures. This information is essential for*

interpreting the COx yields. For example, at elevated temperatures (e.g., 150 °C), are the higher COx yields simply a consequence of operating beyond full conversion, thereby leading to over-oxidation of primary products? Explicit reporting of conversion and reaction times is necessary to clarify this point.

Response: We thank the reviewer for this important comment. We confirm that all reactions were conducted with a fixed reaction time of 12 h, and we have now explicitly reported both reaction times and polymer conversions in the revised figures. We acknowledge that at elevated temperatures (e.g., 150 °C), full polymer conversion is achieved, which raises the reasonable concern that higher CO_x yields may result from over-oxidation of primary products. To address this directly, we conducted a control experiment in which mixed diacids ($C_{4\text{-}8}$) was subjected to the reaction conditions. Negligible CO_x generation was observed (Fig. S27), confirming that the primary diacids products are stable under these conditions and are not the source of CO_x. The elevated CO_x yields at higher temperatures therefore reflect an intrinsic characteristic of the radical oxidation pathway rather than secondary over-oxidation of the target products. We believe this clarification, together with the addition of conversion data, fully addresses the reviewer's concern.

The specific revisions are outlined below:

Page 8 line 28 in revised manuscript:

Further oxidation of short-chain diacids was minimal, as demonstrated by control experiments using commercial diacids with chain lengths of $C_{4\text{-}8}$ (Fig. S27), confirming that the elevated CO_x yields at higher temperatures stem from the initial radical-mediated scission pathways rather than the secondary overproduction of CO_2.

Page 36 in the Supplementary Information:

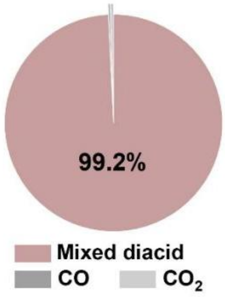

Fig. S27. Stability test of mixed dicarboxylic acids ($C_{4\text{-}8}$) under standard reaction conditions (150 °C, 3 MPa O_2 in 5 mL H_2O, 12 h). The minimal CO_x evolution (<1%) confirms the robust stability of the primary upcycling products against over-oxidation.

Page 28-31 in the Supporting Information:

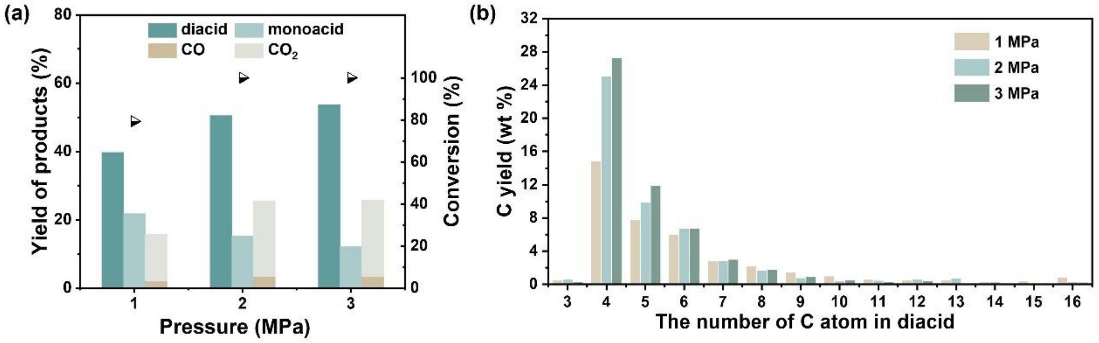

Fig. S18. (a) The yield of products of PE oxidation under different pressure of O_2 at 125 °C after 12 h. (b) Distribution of diacid generated under different pressure of O_2 at 125 °C after 12 h.

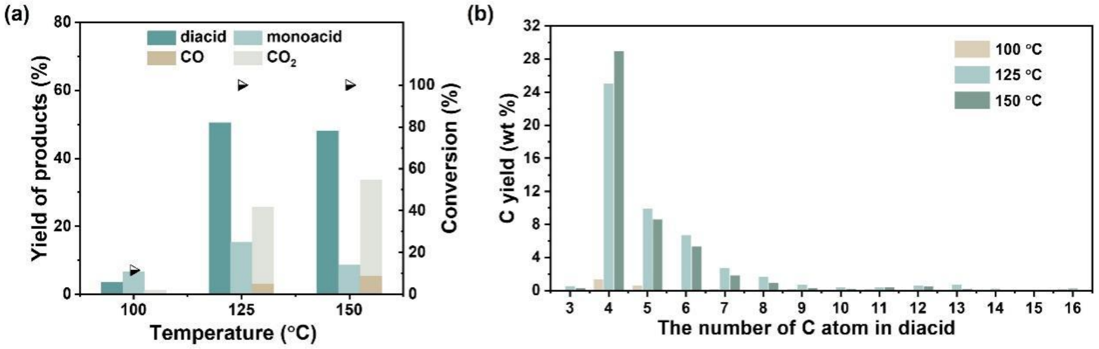

Fig. S19. (a) The yield of products of PE oxidation under different temperature under 2 MPa O_2 after 12 h. (b) Distribution of diacid generated under different temperature under 2 MPa O_2 after 12 h.

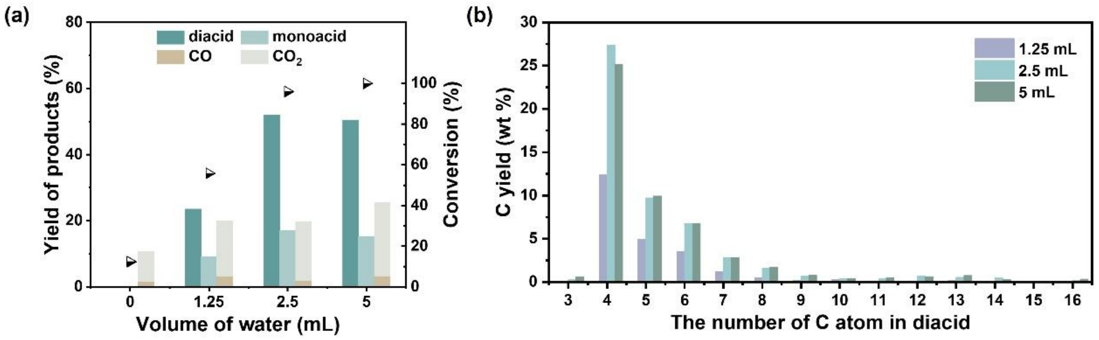

Fig. S21. (a) The yield of products of PE oxidation under different volume of water after 12 h. (b) Distribution of diacid generated under different volume of water after 12 h. Notably, at 125 °C and 2 MPa O_2, the gas-phase oxygen concentration is ~0.604 mol L^{-1} regardless of liquid water volume, while the equilibrium dissolved O_2 concentration in water is ~0.107 mol L^{-1} (from the ideal gas law and Henry's law).

Comment 2. *Results, Lines 255-257 & Comment 15 (Mechanistic Clarification): The authors state that "Initially, interfacial •OH abstracts hydrogen atoms from the PE chains, generating alkyl radicals. Alkyl radicals are then captured by •OH to produce alcohol intermediates (Fig. S27). Further •OH attack forms alkoxyl radicals that undergo β-scission/HAT, producing aldehyde/ketone intermediates." Given the central importance attributed to •OH radicals in Fig. 3E (right panel), one would expect that even under N_2 (in the absence of O_2), some degree of hydroxyl or carbonyl functionalization should be detectable in the recovered polymer. However, FTIR data (Fig. R11) do not appear to show such features. This discrepancy requires clarification. It is possible that FTIR, being surface-sensitive, does not adequately capture bulk modifications. If so, complementary techniques such as NMR may provide more definitive insight.*

Response: We thank the reviewer for this insightful comment. We acknowledge that the absence of detectable functionalization under a N_2 atmosphere appeared inconsistent with the proposed •OH-mediated mechanism. We attribute this to the dilution effect where the limited •OH radicals generated under N_2 were insufficient to produce a detectable concentration of surface species given the high PE-to-water ratio in our previous setup. To address this directly, we optimized the control experiment by reducing the PE loading by half to enhance the relative concentration of functionalized surface sites. As shown in Fig. R1, three complementary characterization techniques now consistently support the occurrence of •OH-mediated surface functionalization (all samples were dried in a vacuum oven at 80 °C before testing):

UV absorbance of TMB solution (Fig. R1a): To further corroborate the presence of •OH under N_2, we employed 3,3′,5,5′-tetramethylbenzidine (TMB) as a colorimetric probe. After 12 h of reaction, the UV-Vis absorbance of the TMB oxidation product reached 0.034 under N_2, compared with 0.22 under O_2. Although the •OH yield under N_2 is much lower than under O_2, the measurable TMB response unambiguously confirms that •OH radicals are generated at the water microdroplet interface even in the absence of oxygen.

Gas-phase mass spectrometry (Fig. R1a): Given that •OH production at the microdroplet interface involves electron and H^+ generation (as depicted in Fig. 3A), we hypothesized that the complementary reduction half-reaction should yield H_2. Gas-phase MS analysis of the headspace after reaction under N_2 revealed an enhanced m/z = 2 signal in the PE + H_2O system relative to H_2O alone, confirming H_2 evolution. This observation is consistent with the proposed mechanism and the process can be enhanced

by the presence of PE as a new water-oil interface.

XPS O 1s (Fig. R1b): The corresponding O 1s spectrum of PE-N_2 exhibits a pronounced component at ~532 eV, attributable to C-O-H/C-O-C functionalities. Additionally, the oxygen-to-carbon intensity ratio (A_O/A_C) of PE recovered after 12 h reaction under N_2 is 7.9×10^{-2}, nearly an order of magnitude higher than that of virgin PE (8.4×10^{-3}). The XPS results therefore provide direct surface-chemical evidence that oxygen incorporation into the PE backbone occurs under N_2, consistent with •OH-mediated oxidation at the microdroplet interface.

Solid-state ^{1}H NMR (Fig. R1c): A new resonance (sharp peak) at 3.4 ppm is clearly resolved, which is assignable to the -CH(OH)- protons of the predicted alcohol intermediates.

Regarding the ^{13}C solid-state NMR, the absence of detectable signals is expected due to the combined effect of the low natural abundance of ^{13}C (~1.1%) and the inherently low degree of oxidation under oxygen-free conditions, which keeps the total carbon-oxygen species below the instrument's sensitivity limit.

Collectively, these multi-spectroscopic results resolve the initial discrepancy and provide robust evidence for the •OH-initiated hydrogen abstraction and subsequent surface hydroxylation steps.

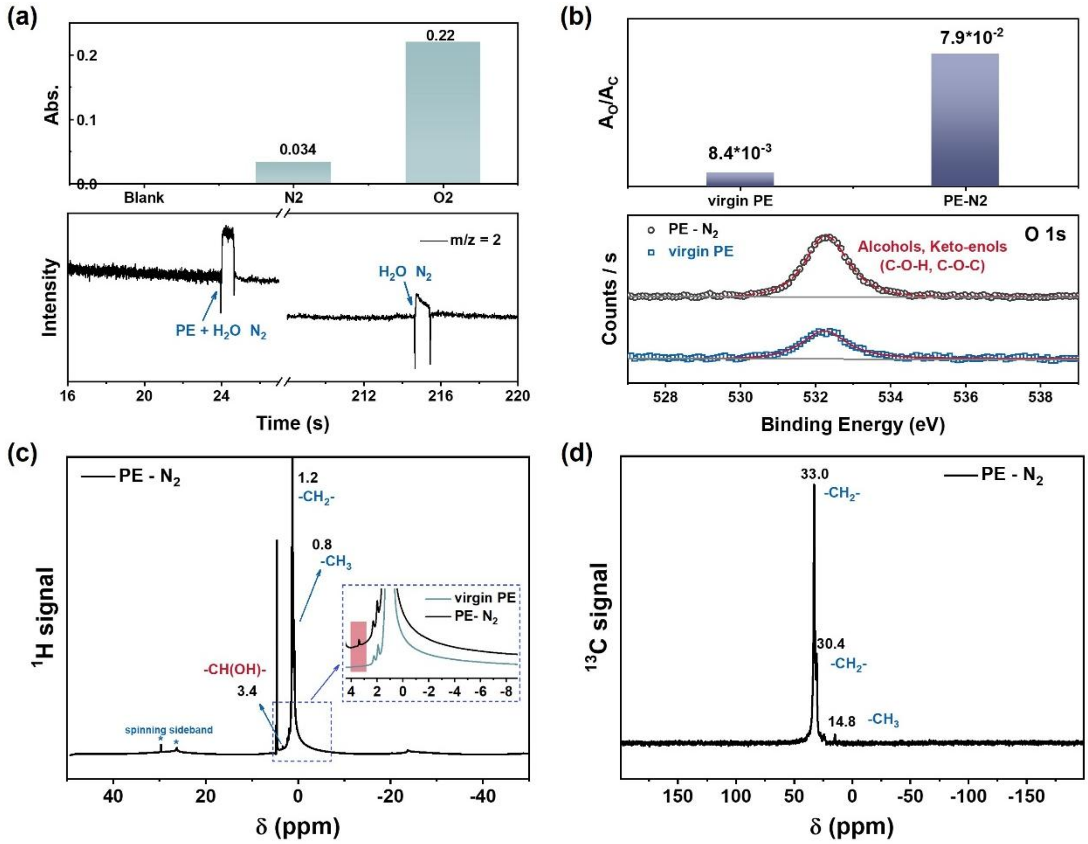

Fig. R1. (a) UV absorbance of the solution (0.5 ml 2mM TMB/DMSO+5ml H_2O) under

N_2 and O_2 after 12 h at 125 °C. And the gas-phase online MS of the PE +H_2O and pure H_2O systems. (b) XPS O 1s spectra of virgin PE and PE residual solid after reaction under N_2 for 12 h. (c) The solid-state ^{1}H NMR spectrum and (d) solid-state ^{13}C NMR spectrum of virgin PE and recovered PE-N_2 residue.

The specific revisions are outlined below:

Page 20 in the Supporting Information:

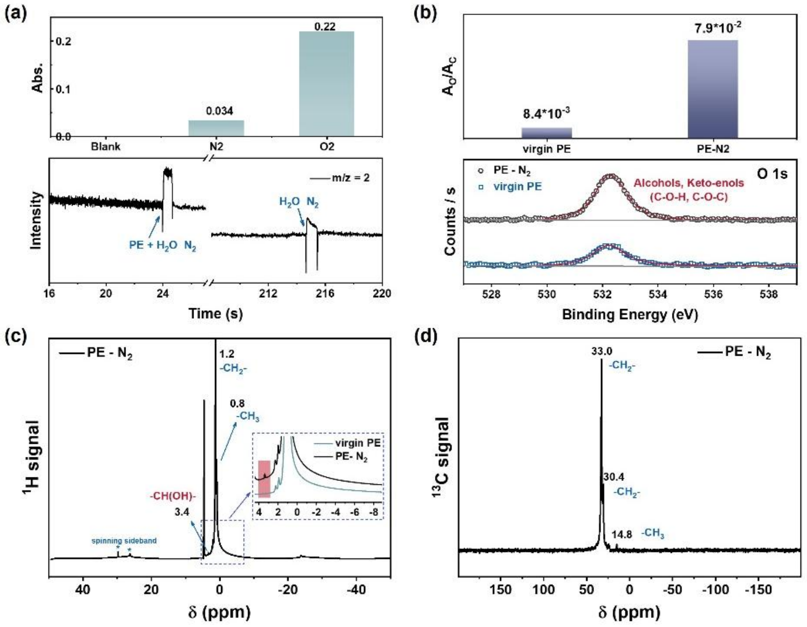

Fig. S10. (a) UV absorbance of the solution (0.5 ml 2mM TMB/DMSO+5ml H_2O) under N_2 and O_2 after 12 h at 125 °C. And the gas-phase online MS of the PE +H_2O and pure H_2O systems. (b) XPS O 1s spectra of virgin PE and PE residual solid after reaction under N_2 for 12 h. (c) The solid-state ^{1}H NMR spectrum and (d) solid-state ^{13}C NMR spectrum of virgin PE and recovered PE-N_2 residue.

UV absorbance of TMB solution (Fig. R1a): To further corroborate the presence of •OH under N_2, we employed 3,3′,5,5′-tetramethylbenzidine (TMB) as a colorimetric probe. After 12 h of reaction, the UV-Vis absorbance of the TMB oxidation product reached 0.034 under N_2, compared with 0.22 under O_2. Although the •OH yield under N_2 is much lower than under O_2, the measurable TMB response unambiguously confirms that •OH radicals are generated at the water microdroplet interface even in the absence of oxygen.

Gas-phase mass spectrometry (Fig. R1a): Given that •OH production at the microdroplet interface involves electron and H^+ generation (as depicted in Fig. 3A), we hypothesized that the complementary reduction half-reaction should yield H_2. Gas-phase MS analysis of the headspace after reaction under N_2 revealed an enhanced m/z

= 2 signal in the PE + H_2O system relative to H_2O alone, confirming H_2 evolution. This observation is consistent with the proposed mechanism and the process can be enhanced by the presence of PE as a new water-oil interface.

XPS O 1s (Fig. R1b): The oxygen-to-carbon intensity ratio (A_O/A_C) of PE recovered after 12 h reaction under N_2 is 7.9×10^{-2}, nearly an order of magnitude higher than that of virgin PE (8.4×10^{-3}). The XPS results therefore provide direct surface-chemical evidence that oxygen incorporation into the PE backbone occurs under N_2, consistent with •OH-mediated oxidation at the microdroplet interface.

Solid-state ^{1}H NMR (Fig. R1c): A new resonance (sharp peak) at 3.4 ppm is clearly resolved, which is assignable to the -CH(OH)- protons of the predicted alcohol intermediates.

Comment 3. *The authors state "Since the oxidation is exothermic, the temperature can be set slightly below the polymer's melting point to limit over-oxidation. Polypropylene (PP) and polystyrene (PS) were selectively converted to acetic acid and benzoic acid at 125 °C and 220 °C, respectively, driven by the reactivity of tertiary sp3 carbons (Fig. S32)." While this explanation may be plausible, the mechanistic picture remains unclear. The proposed mechanism relies on microdroplet formation, which presumably requires the polymer to be in a molten state. However, polypropylene typically melts at ~160–170 °C, yet significant conversion is reported at 125 °C. If the polymer is not molten under these conditions, it is difficult to rationalize how the proposed microdroplet-mediated mechanism operates. This represents a mechanistic inconsistency that must be addressed. Additionally, providing DSC data for all tested polymers would be highly valuable, both to justify the chosen reaction temperatures and to clarify phase behavior under operating conditions.*

Response: We thank the reviewer for this important mechanistic comment. DSC data for all tested polymers have been provided in the revised Fig. S32. We acknowledge that the PP used in our experiments exhibits a melting range of 147-156 °C as determined by DSC (Fig. S32), which is indeed above the nominal reaction temperature of 125 °C. However, we propose the following mechanistic rationale to address this apparent inconsistency.

First, as demonstrated in Figs. S6-7 and Fig. 2D, hydroxyl radicals are generated within the system even in the absence of polymer, attributed to microdroplet formation at the gas-liquid interface at elevated temperatures and pressure. This indicates that •OH generation is not strictly contingent on a liquid-liquid (water-oil) interface; rather, surface oxidation of solid or thermally softened polymer particles can be initiated by

•OH species generated at the highly energized gas-water interface.

Second, since the oxidation reaction (via gas-water interfacial radical) is exothermic, the localized thermal effect arising from the exothermic oxidation may facilitate further softening, creating a positive feedback loop that promotes oxidative chain scission.

Third, the Vicat softening point of PP (~80-120 °C) is well below the reaction temperature of 125 °C, indicating that the polymer surface undergoes significant softening and deformation under the combined effects of temperature and mechanical agitation. This may generate sufficient polymer-water interfacial area to sustain •OH-mediated oxidative attack even in the absence of complete melting. In contrast, the low conversion of PE at 100 °C (Fig. S19) reflects the combined effect of insufficient •OH generation at the gas-liquid interface at this temperature (Fig. S6) and the high crystallinity of PE (~60-80%), which prevents meaningful thermal softening below its melting point.

Fourth, the intrinsically higher reactivity of PP toward •OH-mediated hydrogen abstraction provides an additional chemical basis for its conversion at sub-melting temperatures. The PP backbone contains tertiary C–H bonds at every repeat unit, which are significantly more susceptible to •OH attack than the secondary C-H bonds in PE due to the greater stability of the resulting tertiary carbon radical—stabilized by hyperconjugation with three adjacent alkyl substituents—compared to the secondary radical formed upon H-abstraction from PE. This difference in radical stability translates directly into a lower activation energy for the hydrogen abstraction step, enabling efficient •OH-mediated oxidation of PP even under conditions of partial softening rather than complete melting.

Additionally, PS with a T_g of ~93 °C (Fig. S32) is well above its softening point at the reaction temperature of 220 °C, confirming the validity of the proposed pathway when the polymer is in a fully softened state. To further illustrate the role of thermal softening, photographs of PS before and after heating to 125 °C are provided in Fig. R2, clearly showing significant deformation and flow of the polymer.

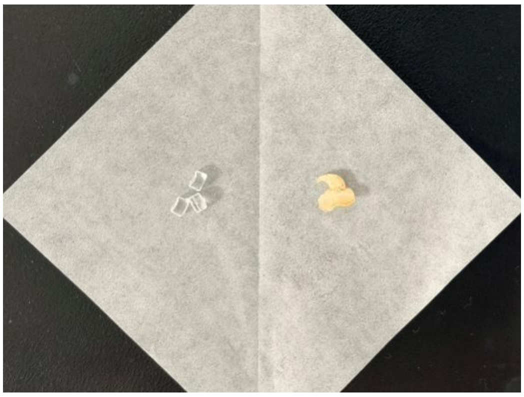

Fig. R2. Photographs of PS pellets before (left) and after (right) heating to 125 °C.

Taken together, these observations reconcile the apparent mechanistic inconsistency: the process can be initiated by gas-liquid interfacial •OH generation and surface oxidation enabled by thermal softening of PP, with the exothermic nature of the reaction driving a self-reinforcing oxidation cycle.

The specific revisions are outlined below:

Page 10, line 4 in the revised manuscript:

The process was extended to other polymers by tuning the temperature to match their dispersion behavior (Fig. 4C, D). Since the oxidation is exothermic, the operating temperature can be effectively set near or below the polymer's melting point (Fig. S32), as thermal softening combined with mechanical agitation ensures sufficient interfacial contact to initiate •OH-mediated oxidation.

Page 41 in the Supporting Information:

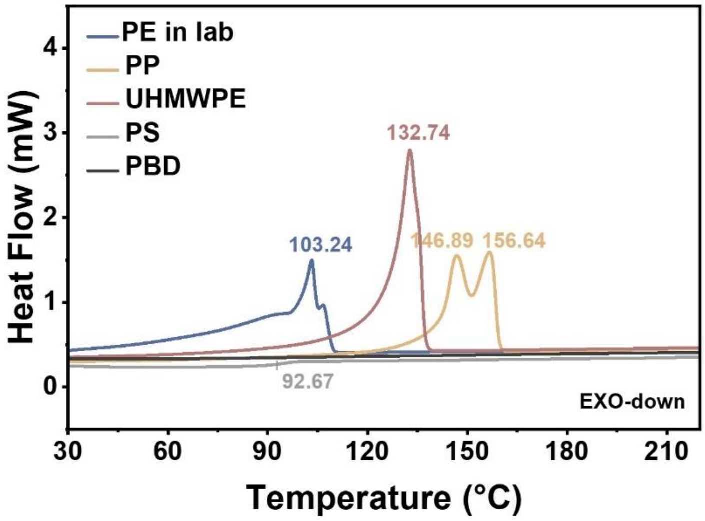

Fig. S32. DSC curves of various polymers in lab.

Comment 4.1. *Technoeconomic Analysis: Although the authors have expanded the discussion of the technoeconomic analysis, the underlying assumptions are still not*

described with sufficient depth or transparency. Several key parameters remain vague, and critical inputs are either insufficiently justified or not explicitly stated. Greater clarity and a more rigorous presentation of assumptions are necessary to enable proper evaluation of the robustness and credibility of the analysis.

Feedstock sourcing: It is unclear how and from where the polyethylene feedstock is sourced. Because the proposed process relies on a relatively pure polyethylene stream rather than mixed plastic waste, collection, sorting, and transportation costs should be explicitly included. Numerous technoeconomic studies of plastic chemical recycling have shown that these upstream logistics are among the largest cost drivers in the overall process, including work by Beckham et al. (Energy Environ. Sci., 2023, 16, 3638–3653) and Hernández et al. (ACS Sustainable Chemistry & Engineering, 2023, 11, 7170–7181). These costs must either be incorporated into the model or clearly justified if excluded.

Response: We thank the reviewer for raising this important point. We fully recognize that feedstock sourcing and upstream logistics costs are critical considerations in technoeconomic analyses of plastic recycling processes. While we acknowledge the significance of the logistics drivers highlighted by Beckham *et al*. and Hernández *et al.*, we opted for a feedstock price of ~$0.83/kg. This value significantly exceeds the $0.30-0.60/kg range typically cited for waste PE streams in those studies, as well as the $0.60/kg benchmark used in other high-profile TEA models (e.g., Science 381, 666-671 (2023); Science 381, 660-666 (2023)). This pricing assumption represents a conservative intermediate estimate that implicitly incorporates basic sorting, cleaning, and handling costs, while avoiding the over-optimism of assuming access to raw, low-cost mixed waste. Consequently, the economic advantages demonstrated in our analysis are established under more stringent feedstock cost conditions than those typically used for mixed waste PE. Should lower-cost waste PE be utilized, the overall economic attractiveness of our process would further increase. To further confirm the robustness of our model, we performed a sensitivity analysis (Fig. S36), which demonstrates that even if the feedstock price increases to $1.0/kg, the process remains economically viable. We have added an explicit statement of this assumption and its justification to the revised manuscript.

The specific revisions are outlined below:

Page 11, line 11 in the revised manuscript:

To ensure conservative and transparent assumptions, the PE feedstock was priced at ~$0.83/kg. This price represents an intermediate estimate that reflects the costs of

sorting, cleaning, and transportation, as it aligns with the lower bound of virgin PE market prices and consistently exceeds the waste PE costs used in recent chemical recycling studies (~$0.30-0.60/kg) [52,53]…

Page 45 in the Supporting Information:

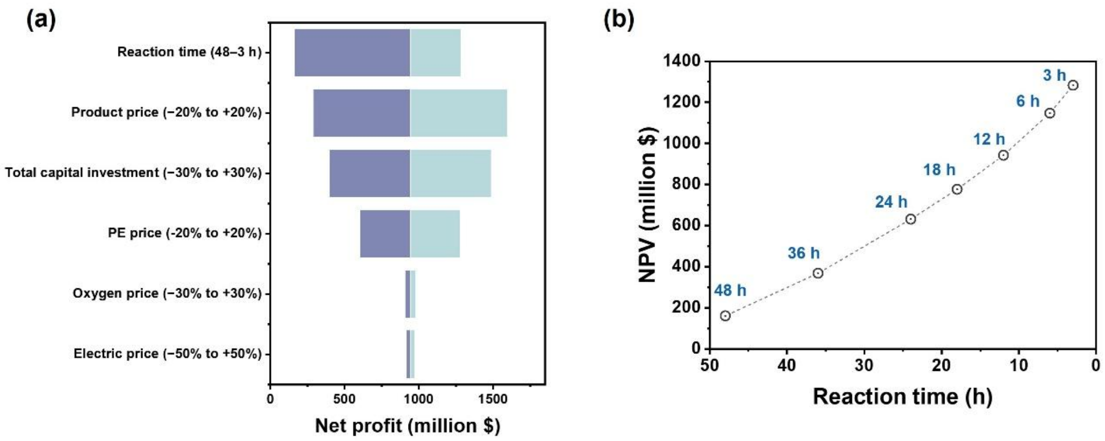

Fig. S36. (a) One-way sensitivity analysis of net present value (NPV). (b) Sensitivity analysis of net present value (NPV) as a function of reaction time.

Comment 4.2. *MEA-based CO_2 capture: The authors state that residual CO2 is captured using an MEA-based absorption unit; however, no explicit capital or operating costs are presented. It is unclear whether this unit is included within the "Gas separator" category. MEA-based CO2 capture typically requires approximately 3.5–5.0 GJ per tonne of CO_2 for solvent regeneration, significantly affecting both CAPEX and OPEX. It is unclear whether these energy demands and associated costs have been accounted for.*

Response: We sincerely thank the reviewer for this meticulous observation. We acknowledge that while the MEA-based CO_2 capture unit was included in our environmental impact model (LCA), its specific capital (CAPEX) and operating (OPEX) costs were inadvertently omitted from the initial technoeconomic analysis (TEA). We have now fully incorporated these costs into the revised TEA to ensure transparency and rigor.

Guided by the reviewer's suggestion and industry benchmarks, we have updated the model with the following parameters:

Energy Consumption: We adopted a conservative regeneration energy requirement of 4.0 GJ per tonne of CO_2, utilizing ~70,000 tonnes/year of low-pressure steam.

Operating Costs: The associated electricity for auxiliary equipment (pumps/blowers, ~2×10^6 kWh/year) and MEA solvent makeup (estimated at 1.0

kg/tonne CO_2 at $1,500/tonne) have been explicitly added.

Capital Investment: The CAPEX for the MEA absorption and stripping columns has been integrated into the Capital Investment (Table S8).

These additions result in a total annual operating cost increase of approximately $2.5M (a ~4% increase), raising the total OPEX to $64.27M. Importantly, as shown in the updated Table S6-10, this revision does not materially alter the primary economic conclusions: the payback period remains highly competitive at ~3.3 years, and the internal rate of return (IRR) remains robust. To maintain a conservative estimate, we continue to exclude potential revenue from the sale of captured industrial-grade CO_2.

The specific revisions are outlined below:

Page 11, line 22 in the revised manuscript:

At a representative capacity of 60 kt yr^{-1}, the plant requires a total capital investment of $144 million (Tables S6–S10), yielding an annual after-tax net profit of $72.1 million and a payback period of ~3.3 years without government subsidies (Fig. 5D). Crucially, the industrial model assumes a 12 h residence time, predicated on a continuous plug-flow reactor (PFR) design (Fig. S35). Unlike the unoptimized batch system used in our laboratory scale-up—which required 48 h due to mass-transfer limitations—the PFR utilizes a heated screw feeder and distributed gas injection to maximize interfacial renewal and radical flux. Sensitivity analysis (Fig. S36) confirms that the process remains economically viable even if the residence time extends to 48 h, with the net present value (NPV) remaining positive across this entire range. The breakeven capacity is ~9.0 kt yr^{-1}, demonstrating operational resilience to feedstock volatility and supporting decentralized deployment at sub-nominal scales. While product pricing and capital expenditure remain the primary drivers of profitability, the strong influence of residence time on NPV highlights the importance of reactor engineering in minimizing capital intensity.

Page 54-57 in the Supporting Information:

Tables S6-10.

Note on CO_2 Capture Unit: The MEA absorption system is designed for a 50% capture efficiency. Solvent regeneration energy is set at 4.0 GJ/t CO_2, sourced from low-pressure steam and electricity. MEA loss is accounted for at a rate of 1.0 kg/t CO_2. All associated utility and capital costs are reflected in the updated financial indicators.

Comment 4.3. *Retention time: In the industrial upscaling framework, a 12 h residence time is assumed. However, the authors' own scale-up experiments required 48 h to achieve performance comparable to laboratory-scale results. This discrepancy is*

substantial and must be justified quantitatively. The choice of a 12 h residence time appears optimistic and may materially affect projected economics.

Response: We thank the reviewer for this critical engineering perspective. We clarify that the 48 h residence time observed in our current scale-up experiments reflects the performance of an unoptimized, large-volume batch reactor, where significant heat and mass transfer limitations (specifically oxygen diffusion and interfacial renewal) have not yet been addressed.

To bridge this gap, our TEA is based on a designed Plug-Flow Reactor (PFR) concept for continuous industrial operation (Fig. S35b). The PFR configuration offers several fundamental advantages that justify the 12 h residence time target:

Continuous Interfacial Renewal: The use of a heated screw feeder ensures a consistently high surface-area-to-volume ratio through continuous microdroplet generation.

Enhanced Mass Transfer: Segmented O_2 supply and precise temperature control along the reactor length eliminate the "dead zones" and oxidant starvation common in large batch systems.

Recognizing the inherent uncertainty at this stage, we have performed a comprehensive sensitivity analysis (Fig. S36). As shown in Fig. S36b, while the NPV is sensitive to residence time (varying from ~$1,283M at 3 h to ~$161M at 48 h), the process remains economically viable with a positive NPV even under the most conservative 48 h scenario. This demonstrates that our economic case is fundamentally robust, even if the anticipated engineering optimizations in the PFR framework are only partially realized. We have added these justifications and the stress-test data to the revised manuscript.

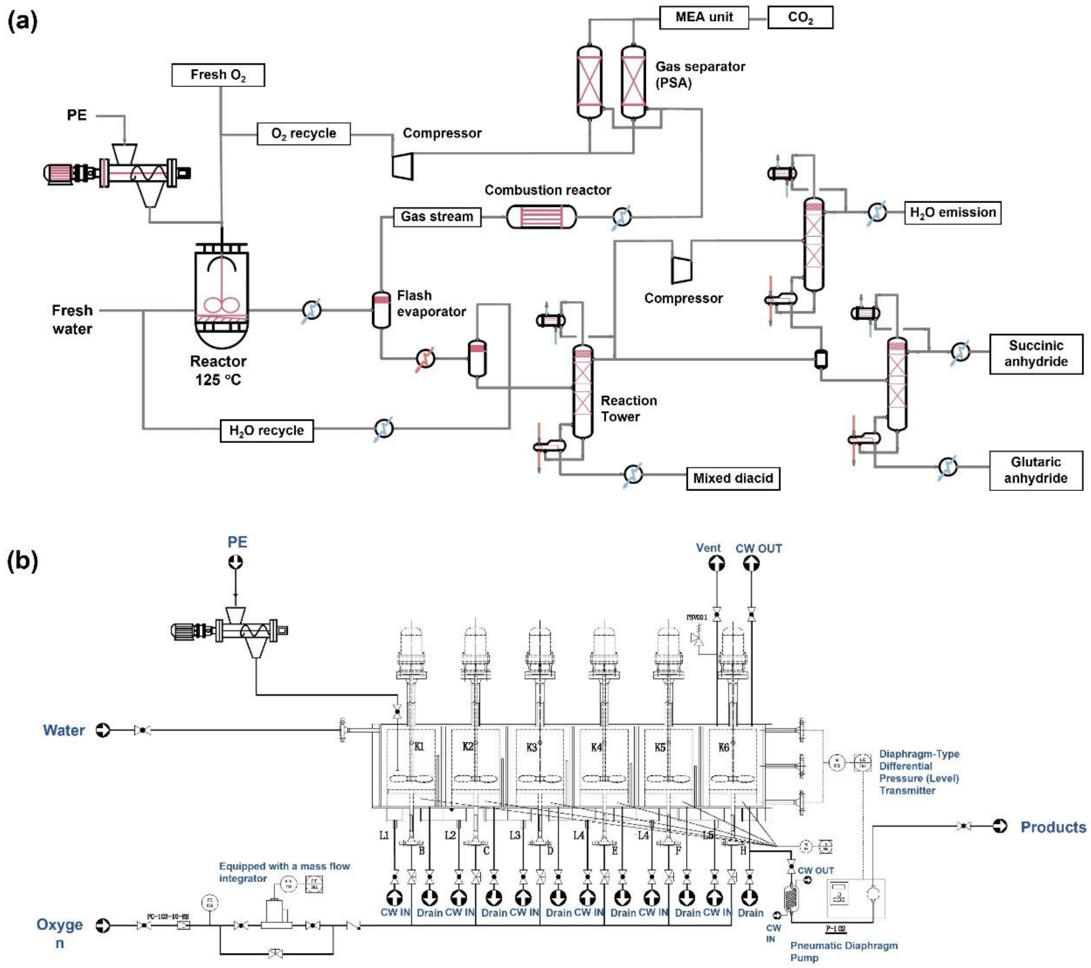

Fig. S35. (a) Proposed process flow diagram to produce diacid from waste PE via catalyst-free oxidation. (b) Process flow diagram of the plug-flow reactor designed for continuous microdroplet oxidation of polymers.

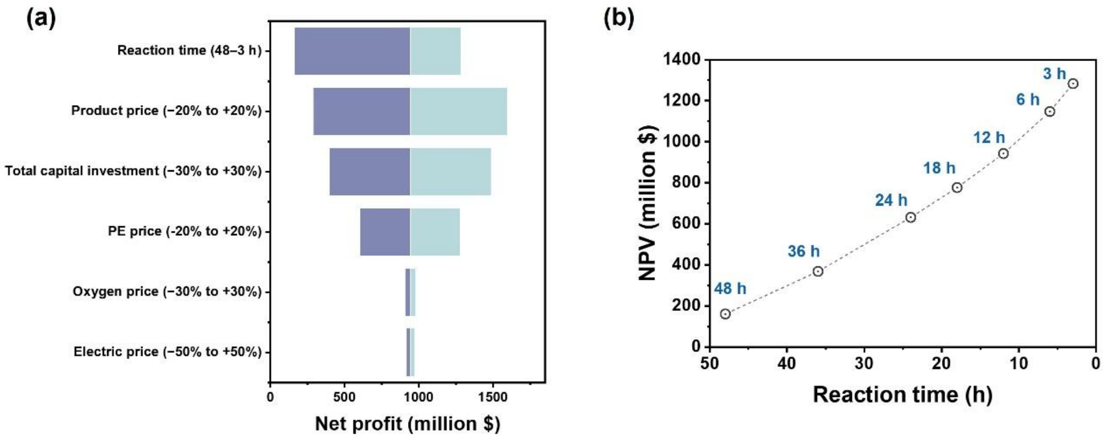

Fig. S36. (a) One-way sensitivity analysis of net present value (NPV). (b) Sensitivity analysis of net present value (NPV) as a function of reaction time.

The specific revisions are outlined below:

Page 12, line 2 in the revised manuscript:

Crucially, the industrial model assumes a 12 h residence time, predicated on a continuous plug-flow reactor (PFR) design (Fig. S35). Unlike the unoptimized batch system used in our laboratory scale-up—which required 48 h due to mass-transfer limitations—the PFR utilizes a heated screw feeder and distributed gas injection to maximize interfacial renewal and radical flux. Sensitivity analysis (Fig. S36) confirms that the process remains economically viable even if the residence time extends to 48 h, with the net present value (NPV) remaining positive across this entire range.

Page 5 in the Supporting Information:

Reactor design and throughput optimization: The TEA utilizes a 12 h residence time assumption based on a continuous PFR configuration. This design addresses the limitations of batch reactors by enabling: (i) continuous microdroplet generation, (ii) segmented thermal management, and (iii) distributed oxygen supply. These features are engineered to substantially improve kinetic performance relative to non-optimized batch systems. The economic impact of varying residence times from 3 h to 48 h is detailed in the sensitivity analysis in Fig. S36.

Page 45 in the Supporting Information:

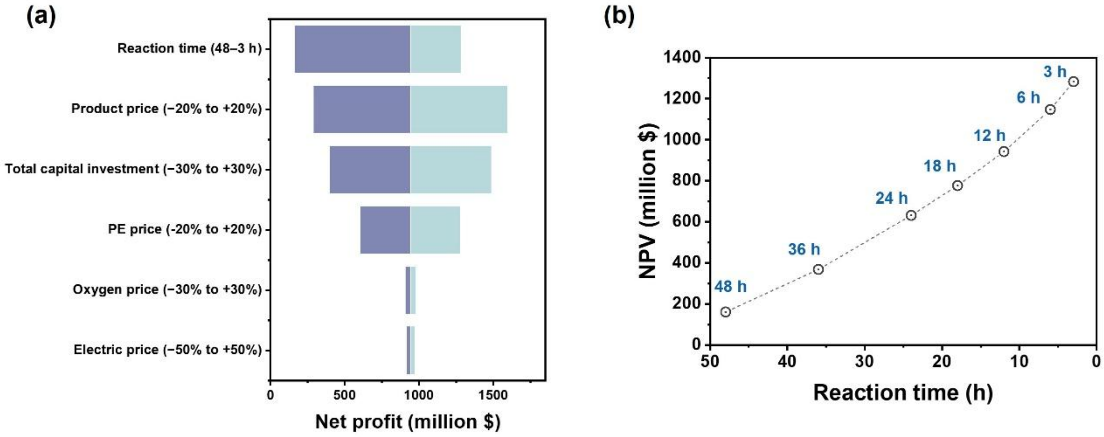

Fig. S36. (a) One-way sensitivity analysis of net present value (NPV). (b) Sensitivity analysis of net present value (NPV) as a function of reaction time.

Comment 4.4. *Water recycle assumption: The authors state that recovered water is recycled with a 1% purge stream to prevent impurity accumulation. The basis for selecting 1% is not explained. This assumption should be justified, and its impact on process performance and economics should be analysed.*

Response: We thank the reviewer for this insightful comment. We agree that the initial 1% purge stream assumption required a more rigorous justification. Upon quantitative re-evaluation of the mass balance, we found that a purge stream is, in fact, unnecessary for this specific process, leading us to optimize the design as follows:

The primary concern regarding water recycling is the potential accumulation of dissolved gaseous byproducts (CO_2 and CO). However, our process includes dedicated gaseous outlet streams (flash drums) where the vast majority of these species are removed. A sensitivity analysis comparing a 0% purge (total recycle) vs. a 1% purge showed a negligible difference in steady-state impurity concentrations (only ~0.02% variance in dissolved gas mass fractions). Furthermore, since the primary liquid-phase byproducts (short-chain organic acids) are target products that are recovered during the subsequent distillation/dehydration steps, they do not "accumulate" as inert impurities in the recycled water.

Consequently, we have removed the purge stream from the revised process design. This optimization significantly enhances the environmental and economic profile of the technology by:

Lowering GWP: The Global Warming Potential (GWP) improved from 1.06 to 0.90 kg $CO_{2\text{-eq}}$ (Fig. 5E) due to reduced wastewater treatment requirements and lower carbon loss.

Improving Economics: Operating costs were further reduced as reflected in the updated TEA (Tables S7-10).

We believe this streamlined design, now grounded in quantitative mass balance, fully addresses the reviewer's concern while presenting a more sustainable upcycling framework.

The specific revisions are outlined below:

Page 12 in the revised manuscript:

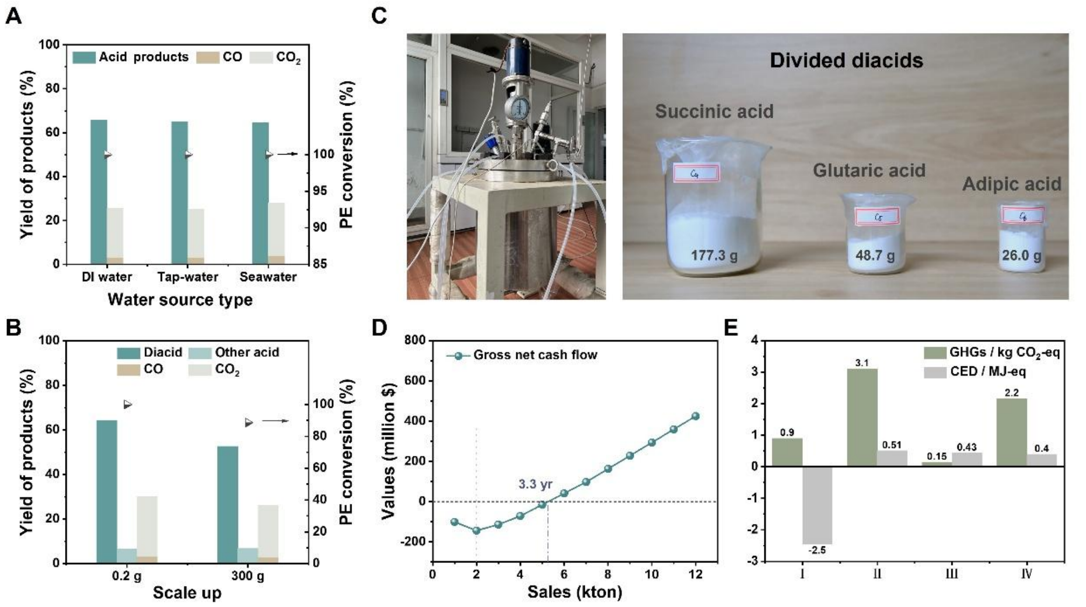

Fig. 5 | Application and technoeconomic analysis of the catalyst-free PE oxidation

strategy. (**A**) Performance evaluation in seawater matrices. (**B**) The scale up experiment on catalyst-free oxidation of PE. (**C**) Reactor and separated products of the scale up experiment. (**D**) The static payback period of this technology model (60 kton). The construction period was set to two years. (**E**) Greenhouse gas emissions (GHGs) and cumulative energy demand (CED) for the treatment of 1 kg of plastic via: (I) the proposed upcycling technology, (II) incineration, (III) landfilling, and (IV) the conventional market-mix technology.

Page 44 in the Supporting Information:

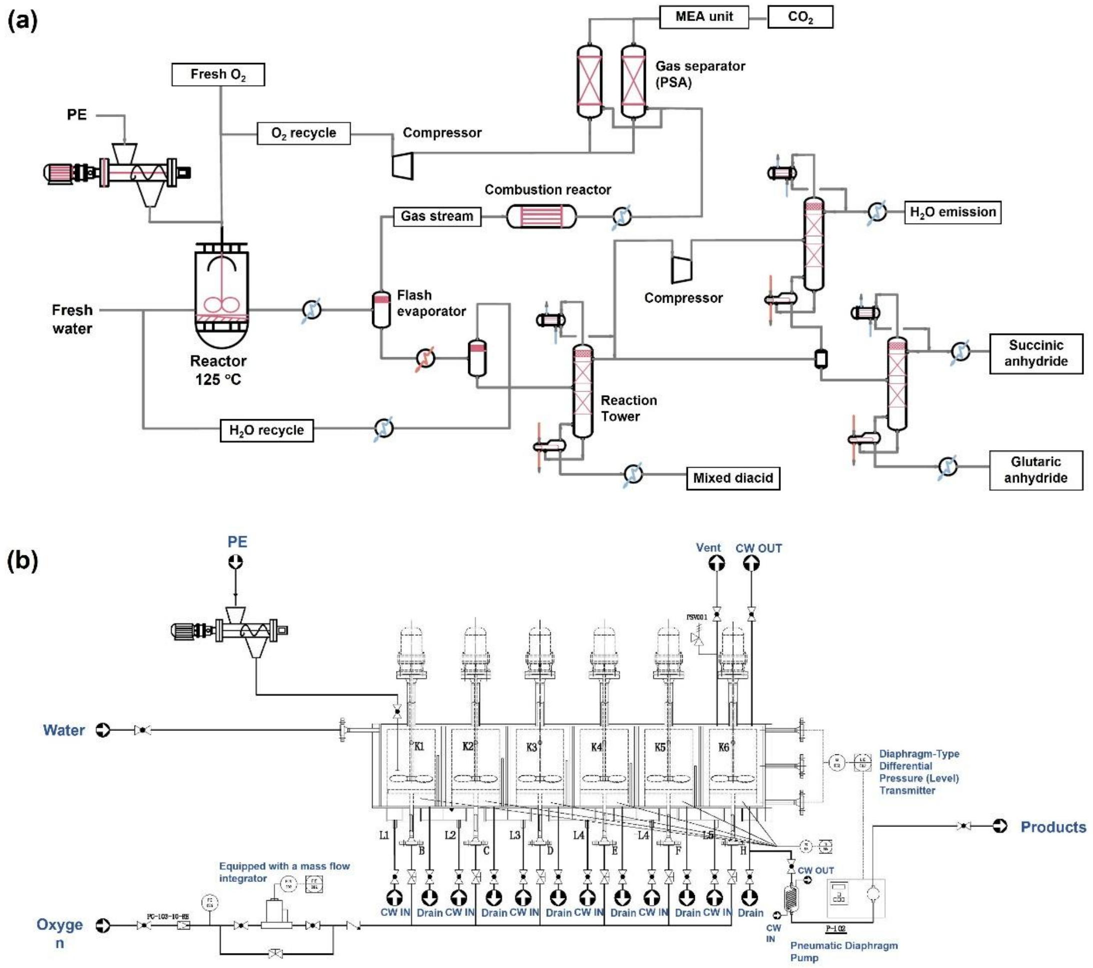

Fig. S35. (a) Proposed process flow diagram to produce diacid from waste PE via catalyst-free oxidation. (b) Process flow diagram of the plug-flow reactor designed for continuous microdroplet oxidation of polymers.

Page 55-57 in the Supporting Information:

Tables S7-10.

Comment 4.5. *Table S8 – "Gas separator" unit: It is unclear which specific operations are included in the "Gas separator" unit. A detailed breakdown is required.*

Response: We thank the reviewer for this request for greater transparency. We have

now provided a detailed engineering breakdown of the gaseous product treatment section. In the revised Table S8, we have clarified that the "Gas separator" refers to a Pressure Swing Adsorption (PSA) system designed for O_2/CO_2 separation. The PSA unit is composed of five primary functional components:

Feed Gas Compressor: To pressurize the inlet stream to the optimal adsorption pressure.

Adsorption Vessels: A multi-bed configuration (packed with Zeolite 13X) for selective CO_2 retention.

Control Manifold: High-frequency switching valves to enable continuous cyclic operation (adsorption/equalization/desorption).

Vacuum Pump: For adsorbent regeneration via pressure swing.

Buffer Tanks: For stabilizing the recycled O_2 and separated CO_2 flows.

Additionally, to protect the adsorbent from thermal degradation and moisture, we have explicitly included the upstream Cooling Heat Exchanger and Knock-out Drum (to maintain ~30 °C) in the capital cost estimation.

As detailed in our response to Comment 4.2, the MEA-based CO_2 capture unit is now listed as a separate downstream operation to avoid overlap. We have also updated the Process Flow Diagram (Fig. S35) with explicit stream labels and unit identifiers to ensure the sequential logic from the combustion furnace to the PSA and MEA units is unambiguous.

The specific revisions are outlined below:

Page 44 in the Supporting Information:

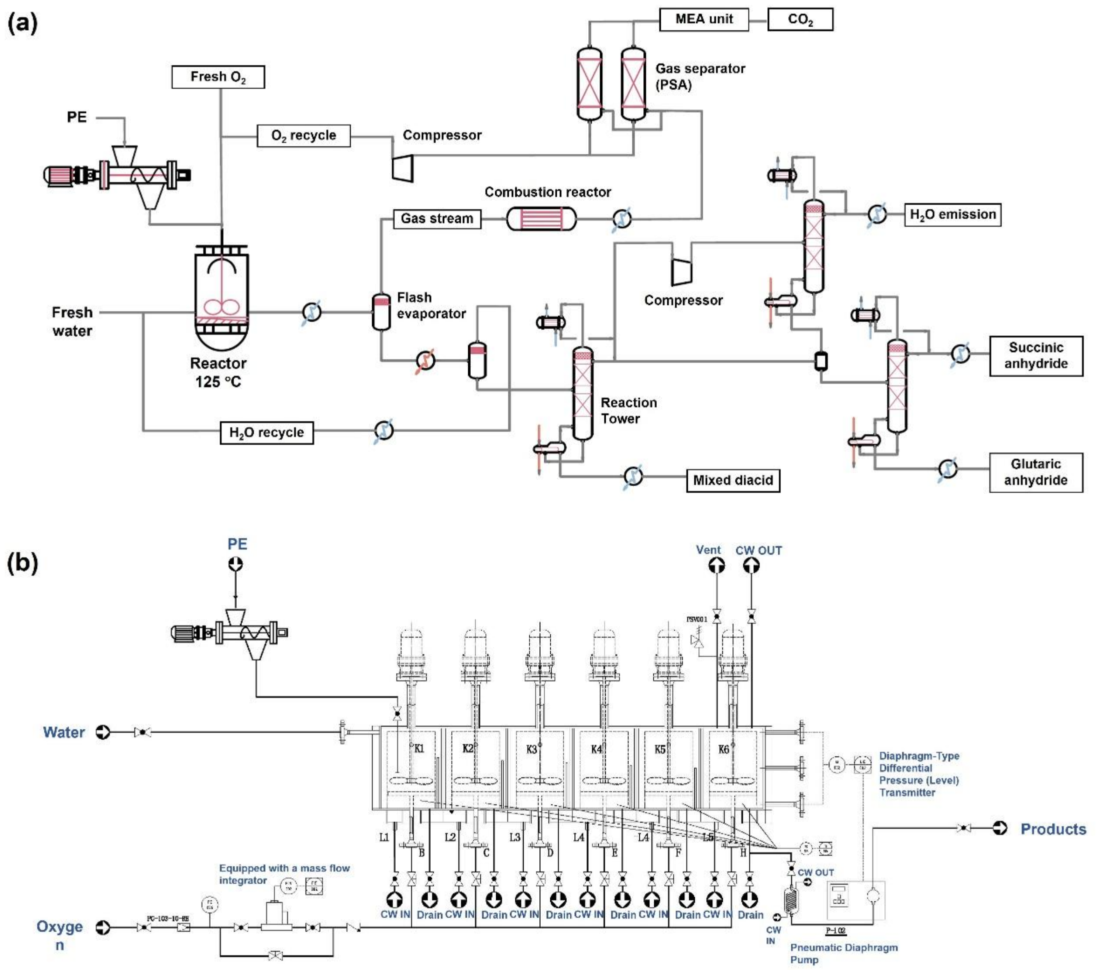

Fig. S35. (a) Proposed process flow diagram to produce diacid from waste PE via catalyst-free oxidation. (b) Process flow diagram of the plug-flow reactor designed for continuous microdroplet oxidation of polymers.

Page 54-57 in the Supporting Information:

Tables S6-10.

Comment 5.1. *Lifecycle Assessment: Similar to the TEA, several assumptions underlying the LCA remain unclear or insufficiently justified.*

o The authors state that "Using a gate-to-gate attributional framework, the analysis shows that chemical upcycling reduces CO2 emissions by ~50% and ~66% relative to market-mix treatment and incineration." However, it is unclear what the conventional "market-mix" technology includes—does it refer to mechanical recycling, pyrolysis, or another pathway? No details are provided. Importantly, mechanical recycling, the most mature and widely implemented recycling pathway, is not included in the comparison.

Response: We thank the reviewer for this important comment. The "market-mix" treatment referred to in our LCA corresponds to the "market group for waste

polyethylene" dataset in the ecoinvent 3.11 database (Europe without Switzerland, 2018 – 2024), which represents a production-volume-weighted average of current real-world waste polyethylene treatment pathways, including sanitary and unsanitary landfilling, municipal incineration, and uncontrolled disposal. This dataset reflects the actual treatment mix across European markets rather than any assumed fixed ratio. We have added an explicit description of this assumption and its basis to the revised SI to improve transparency.

Regarding mechanical recycling, we acknowledge its importance as the most widely implemented recycling pathway and have added it as an additional reference point here (Fig. R3). However, we respectfully note that a direct comparison between chemical upcycling and mechanical recycling requires careful interpretation: mechanical recycling is a physical process that preserves polymer chain integrity without chemical bond breaking and reformation, and therefore inherently involves lower energy input and carbon emissions. Critically, however, mechanical recycling is accompanied by progressive degradation of material properties, rendering it a downcycling process. We fully recognize the inherent advantages of mechanical recycling as a first-preference option; however, when downcycled plastics can no longer meet the performance requirements of their intended applications, chemical recycling represents a complementary and necessary solution to recover value from otherwise unrecyclable end-of-life streams. These two pathways therefore serve fundamentally different functions in the plastic waste management hierarchy, and we believe the comparison with market-mix treatment and incineration remains the more appropriate baseline for evaluating the environmental benefit of our approach.

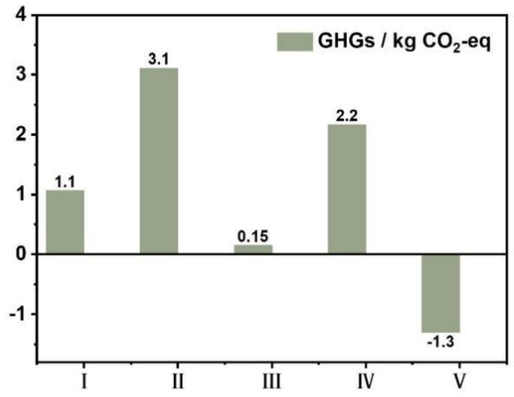

Fig. R3. Greenhouse gas emissions (GHGs) for the treatment of 1 kg of plastic via: (I) the proposed upcycling technology, (II) incineration, (III) landfilling, (IV) the conventional market-mix technology and (V) mechanical recycling.

The specific revisions are outlined below:
Page 8 in the Supporting Information:

Furthermore, energy recovery from incineration and landfilling in conventional scenarios were included, with their respective treatment shares derived from the production-volume-weighted market mix as defined in the ecoinvent 3.11 "market group for waste polyethylene" dataset (Europe without Switzerland, 2018-2024).

Comment 5.2. *It appears that pretreatment steps (e.g., collection, sorting, and transportation) are either omitted or assumed to be equivalent across all technologies. This assumption is unrealistic. Processes such as landfilling or incineration typically require less preprocessing than a technology that depends on a relatively pure, single-stream polyethylene feedstock. Assuming identical pretreatment burdens may bias the comparison in favour of the proposed technology. These system boundaries and assumptions should be explicitly defined and justified.*

Response: We thank the reviewer for this important observation. We have revised the LCA system boundary description accordingly.

For the conventional baseline scenarios (landfilling and incineration), only waste transportation is included as a pretreatment step, as both processes accept mixed, unsorted plastic waste with minimal preprocessing. This is a conservative assumption that does not disadvantage the baseline scenarios.

For the proposed chemical upcycling process, a comprehensive pretreatment chain is explicitly modeled within the system boundary, encompassing waste PE collection, transportation, sorting, washing, drying, shredding, and pelletization, consistent with the feedstock purity requirements of the process. These pretreatment burdens are therefore fully attributed to the proposed technology in the LCA (Table S11: Market for polyethylene, pellets, recycled {RER} | Cut-off).

To make these differences fully transparent, we have added explicit descriptions of the system boundary assumptions for each scenario to the revised SI, with separate process flow diagrams provided in the updated Fig. S37. We believe these clarifications adequately address the reviewer's concern and confirm that the LCA comparison is not biased in favor of the proposed technology.

The specific revisions are outlined below:
Page 7 in the Supporting Information:

Pretreatment requirements differ substantially across the three scenarios. For landfilling and incineration, only waste transportation is included, as these processes accept mixed

plastic waste with minimal preprocessing — a conservative assumption that does not disadvantage the baseline scenarios. For the chemical recycling process, a full pretreatment chain comprising transportation, sorting, washing, drying, shredding, grinding, and pelletisation is included, consistent with its feedstock purity requirements. System boundary diagrams for all three scenarios are provided in Fig. S37.

Page 46 in the Supporting Information:

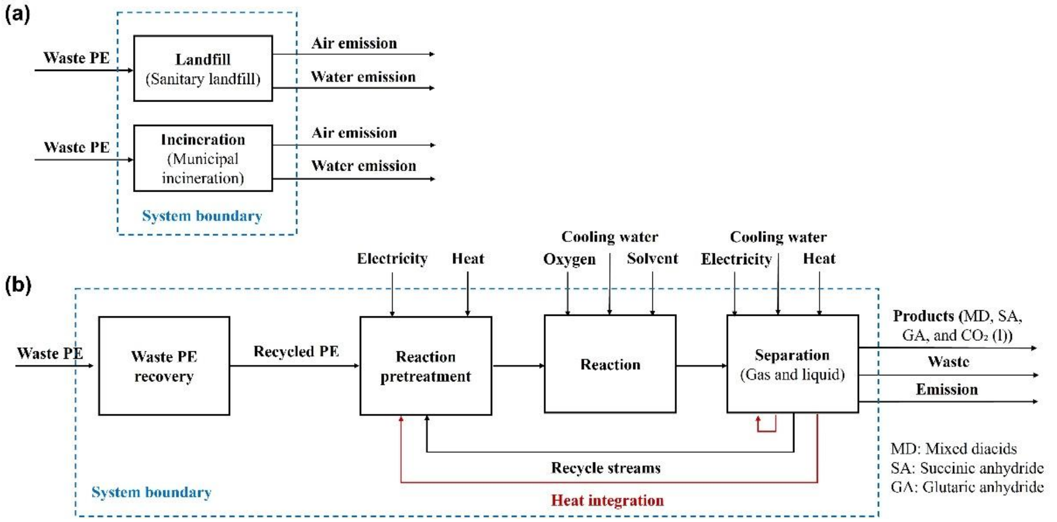

Fig. S37. System boundary for (a) conventional waste PE treatment scenarios (sanitary landfilling and municipal incineration) and (b) the proposed chemical upcycling route.

Comment 5.3. *As in the TEA, the energy demand and emissions associated with MEA-based CO2 capture (including regeneration) must be clearly incorporated into the LCA. It is currently unclear whether these contributions have been accounted for.*

Response: We thank the reviewer for this important clarification request. The energy demand and emissions associated with MEA-based CO_2 capture (including solvent regeneration) are indeed fully incorporated into the LCA. In Table S11, the input entry "Carbon dioxide production, liquid {RER} | Cut-off" represents the entire MEA capture process, including all upstream energy consumption and associated emissions, as documented in the ecoinvent database entry (carbon dioxide production, liquid - Europe - carbon dioxide, liquid | ecoQuery). The dataset includes the material and energy input and emissions for the production of liquid carbon dioxide out of waste gases from different production processes. Estimated water consumption and infrastructure are also included.

To improve clarity, we have revised the comment and add the footnote 1 for the "Carbon dioxide production, liquid {RER} | Cut-off" entry in Table S11 to explicitly state that this inventory entry encapsulates the energy demand and emissions associated

with MEA-based CO_2 capture, including solvent regeneration.

The specific revisions are outlined below:

Page 58 in the Supporting Information:

Carbon dioxide production, liquid {RER} | Cut-off [1]	0.39	kg	Mass and energy flow for MEA-based CO_2 capture (including regeneration)

[1] This background dataset represents the MEA-based CO_2 capture technology.

Comment 6. *Rebuttal, Comment B: The authors argue that "Density-based sorting can efficiently separate low-density polyolefins (PE, PP, PS) from higher-density plastics such as PET, PVC, and PU. PET can be depolymerized via established glycolysis or hydrolysis routes under milder conditions and is better processed separately." While this may hold for virgin resins, consumer-grade plastics frequently contain fillers in significant quantities, which can increase density and compromise separation efficiency. This concern motivated our earlier question regarding the process tolerance to mixed or imperfectly sorted streams. The robustness of the system under such realistic conditions should be demonstrated or more critically discussed.*

Response: We thank the reviewer for this follow-up comment. We acknowledge that consumer-grade plastics frequently contain inorganic fillers that can alter density and compromise the efficiency of density-based separation. As discussed in our previous response (Comment 9), when density-based separation becomes ineffective under such realistic conditions, more advanced sensor-based sorting technologies are available at the commercial scale. In particular, systems such as Autosort™ FLAKE integrate near-infrared (NIR) spectroscopy, high-resolution imaging, metal detection, and AI-assisted classification, and are capable of discriminating and automatically separating PET, PVC, PP, PE, PS, PA, and other common polymer types including polymer alloys. The technical specifications of such commercial equipment further underscore their industrial viability: with working widths of 1,000-2,000 mm, 2-4 chute configurations, and a throughput capacity of up to 6 tonnes per hour, these systems are already deployed in operational waste plastics recovery facilities and are well-suited to handle imperfectly sorted or filler-containing streams. We have expanded this discussion in the revised manuscript to more critically address the robustness of the feedstock preparation step under realistic sorting conditions.

The specific revisions are outlined below:

Page 6 in the Supporting Information:

Feedstock logistics: Establishing robust preprocessing systems compatible with heterogeneous commercial plastic waste streams, including advanced spectroscopic sorting (e.g., Autosort^{TM} FLAKE integrate near-infrared spectroscopy, high-resolution imaging, metal detection, and AI-assisted classification) for polymer identification and separation, followed by cleaning.

Comment 7. *It would strengthen the manuscript to incorporate results from realistic feedstocks (e.g., Fig. S30 and Fig. R2/R8) into Fig. 4, to demonstrate process robustness. Likewise, results from Fig. R3 should be included in the main text or Supporting Information to substantiate claims regarding multicomponent waste tolerance.*

Response: We thank the reviewer for this suggestion. In response, we have incorporated the results from realistic feedstocks into the revised Fig. 4. Additionally, the results from Fig. R3 regarding multicomponent waste tolerance have been included in the revised Supporting Information. We believe these additions substantially strengthen the demonstration of the process's practical applicability.

The specific revisions are outlined below:

Page 10 line 12 in the revised manuscript:

The method also degraded challenging rubber-based materials and multilayer packaging films (Fig. 4D). … Post-consumer packaging bags were similarly processed at 220 °C for 12 h, yielding diacids as the dominant products (45.4%), with the metallized barrier layer recovered as an Al-rich solid residue confirmed by XRD. Mixed real-world polyolefin waste streams were also co-processed under optimized conditions (Fig. S34), achieving satisfactory conversion and diacid yields, further confirming the practical applicability of the approach.

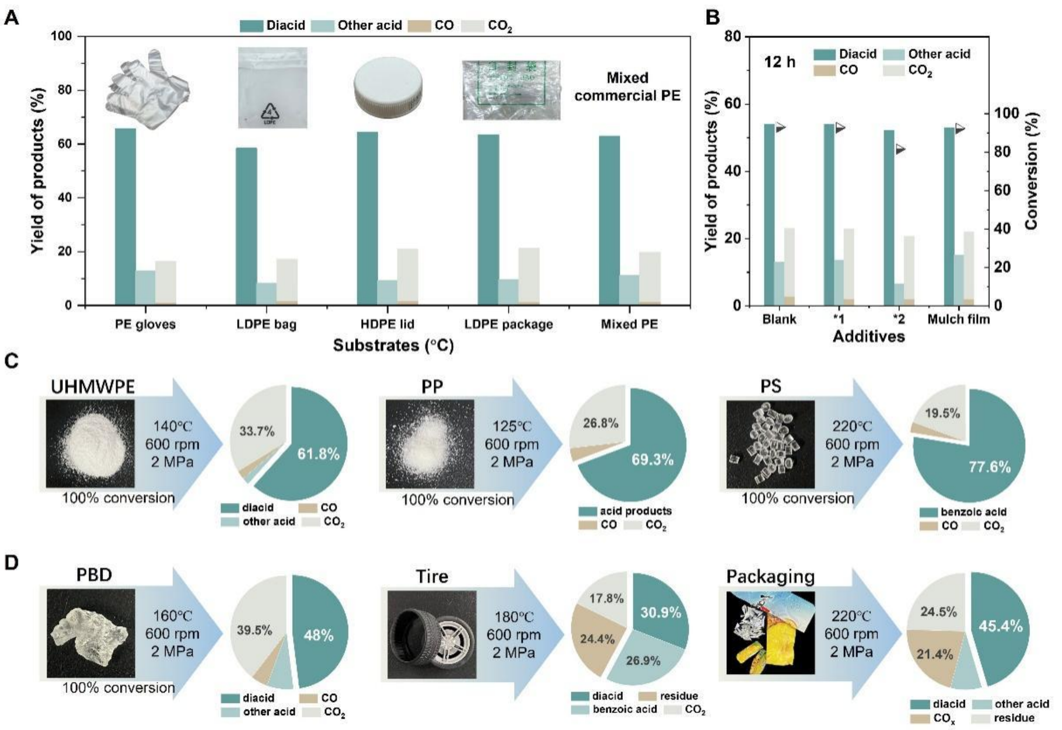

Fig. 4 | The substrate expansion of the catalyst-free PE oxidation strategy. Performance evaluation using (**A**) different commercial PE: PE gloves, LDPE bag, HDPE lid, LDPE package and mixed commercial PE; (**B**) PE under different conditions of additives (*1: Manually add 0.5 wt% BHT, 0.5 wt% Bis(2,2,6,6-tetramethyl-4-piperidyl)sebacate (HALS), 0.4 wt% Irganox 1010 and 1 wt% N-(1,3-dimethylbutyl)-N'-phenyl-p-phenylenediamine (6PPD); *2: twice the manually added amount of *1; Mulch film: agricultural film containing HALS); (**C**) different polyolefins: UHMWPE, PP, PS; (**D**) rubber products: PBD, real-life tire and multilayer packaging films.

Page 43 in the Supporting Information:

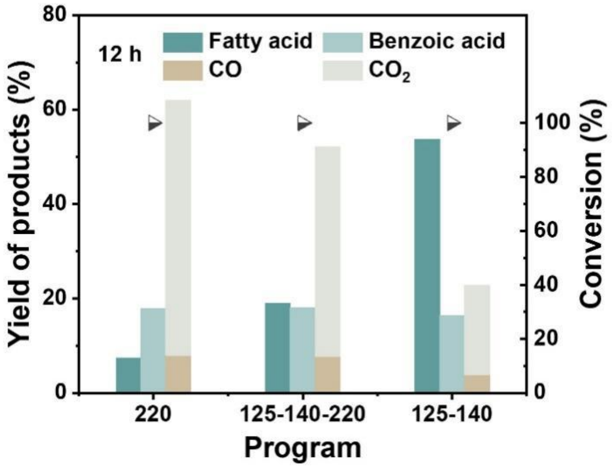

Fig. S34. The product yields from mixed polyolefins (PE, PP, PS) under different heating protocols.

Direct operation at 220 °C led to extensive CO_2 formation, with low diacid yields. A stepped heating protocol (125 °C 3 h, 140 °C 3 h, followed by 220 °C 6 h) similarly increased CO_2 formation, despite a higher diacid yield. In contrast, a milder two-stage protocol (125 °C for 6 h followed by 140 °C for 6 h) provided a more favorable outcome: fatty acid accounted for ~56% of the products, while CO_2 formation was substantially suppressed and benzoic acid yield remained comparable.

In mixed polyolefin blends, PE melts first upon heating and undergoes partial oxidative degradation to generate low-molecular-weight wax-like oligomers, which wet and partially swell the surfaces of higher-melting components such as PP and PS, facilitating their co-dispersion into the aqueous phase as polymer-in-water microdroplets under mechanical agitation. The exothermic nature of PE oxidation further elevates the local temperature, facilitating the processing of higher-melting polymers at nominally sub-melting conditions.

Comment 8. *Rebuttal, Comment E: The manuscript would benefit from the inclusion of a brief discussion of scale-up challenges and engineering considerations.*

Response: We thank the reviewer for this constructive suggestion. In response, we have added a dedicated discussion of scale-up challenges and engineering considerations to the SI (Industry up-scaling framework section), covering reactor design for continuous microdroplet generation, droplet intensification strategies at larger throughput, and key controllable operational parameters. We believe this addition adequately addresses the reviewer's comment.

The specific revisions are outlined below:

Page 5 in the Supporting Information:

At larger scales, established industrial mixing and contacting strategies can be employed:

Continuous plug-flow feeding: A plug-flow configuration with a melt/feeding module and inline emulsification stabilizes droplet formation, limits solids inventory, and allows precise residence-time control, facilitating scale-up.

High-shear and turbulent agitation: A combined impeller strategy can be used: (i) a high-shear impeller (e.g., serrated turbine) in the lower reactor zone fragments molten polymer into fine droplets; (ii) an upper axial-flow impeller circulates material to the shear zone, minimizing dead zones; (iii) gas entrainment along the shaft enhances O_2 or air distribution, improves gas-liquid mixing, reduces slurry density, and promotes sustained oil–water contact.

Additional interfacial intensification: If required, Venturi or other hydrodynamic mixers can generate intense shear and turbulence to produce fine droplets and renew interfacial area. Small amounts of emulsifiers can further stabilize dispersed droplets. Such approaches are widely applied in industrial gas–liquid and solid–liquid–gas processes and are directly compatible with molten-polymer-in-water systems.

Operationally, the key controllable parameters are polymer melting/softening temperature, shear intensity, stirring rate, and oxygen (or gas) management. Collectively, these measures allow replication and intensification of the microdroplet interfacial environment observed in the lab at larger throughput, while maintaining energy efficiency and operational safety using established engineering practices.

Comment 9. *In response to comment 11, the authors suggest that "In mixed polyolefin blends, the low-melting component (PE) softens and melts first and acts as a solvent phase into which higher-melting polymers (PP, PS) dissolve." However, it is well established that polymers are often immiscible even when they possess similar hydrocarbon backbones. For example, polyethylene and polypropylene are generally thermodynamically immiscible in the melt and typically form phase-separated blends in the absence of compatibilizers or specially designed copolymers. Furthermore, in the present system, these polymers are dispersed in water, which would make homogeneous dissolution even less plausible. As written, the explanation appears inconsistent with established polymer physics. The authors should either provide direct evidence supporting this claim or revise the mechanistic interpretation accordingly.*

Response: We thank the reviewer for this important comment and acknowledge that the term "dissolve" used in our previous response was imprecise. We have revised the mechanistic interpretation accordingly.

We propose that the role of PE is not to act as a thermodynamic solvent for PP or PS, but rather to generate low-molecular-weight wax-like oligomers upon partial oxidative degradation, which mediate the dispersion of higher-melting polymers into the aqueous phase through wetting and swelling interactions. To support this, we conducted model experiments using C_{17} alkane as a proxy for the low-molecular-weight wax-like fraction. As shown in Fig. R4, heating C_{17} alkane with PS results in a transparent solution, confirming that low-molecular-weight alkanes can solubilize PS under thermal conditions; upon cooling, PS re-precipitates and the solution turns white and turbid, consistent with thermodynamic phase separation. For PP, immersion in C_{17} alkane at 125 °C results in a gel-like state, indicative of solvent-induced swelling of the

PP amorphous domains rather than true dissolution—a well-established phenomenon in semicrystalline polymers where small-molecule penetrants preferentially swell the non-crystalline regions, reducing surface tension and increasing interfacial accessibility. Under reaction conditions, the in situ-generated low-molecular-weight wax-like fraction is therefore expected to wet and partially swell the surfaces of PP and PS particles, facilitating their co-dispersion with molten PE into the aqueous phase as polymer-in-water microdroplets under mechanical agitation. The exothermic oxidation of PE further elevates the local temperature, reinforcing this co-dispersion process.

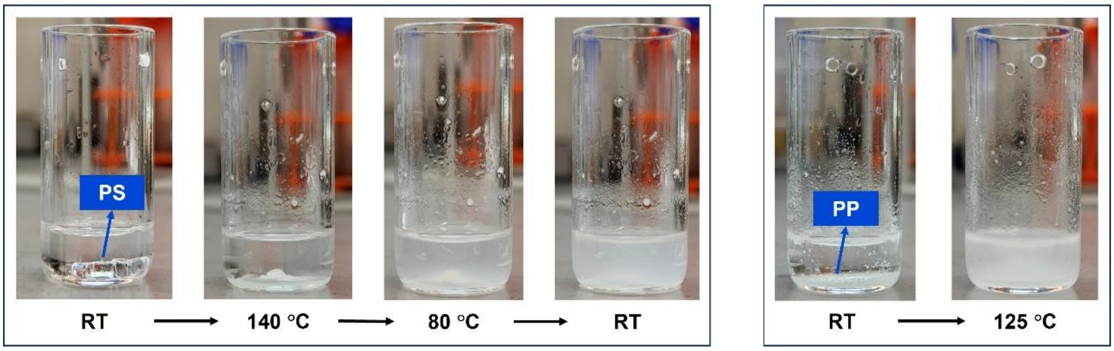

Fig. R4. Photographs demonstrating the interaction of C_{17} alkane with PS (left panel) and PP (right panel) as model experiments for the wax-mediated co-dispersion mechanism.

Direct experimental evidence for the enhanced reactivity of higher-melting polymers in mixed feeds is provided by time-resolved product yield data under the optimized segmented temperature program (125 °C for 6 h followed by 140 °C for 6 h). When PS alone was subjected to these conditions, no benzoic acid was detected after 12 h. In contrast, when PS was co-processed with PE and PP in a mixed feed under otherwise identical conditions, benzoic acid was clearly detected with yield increasing over the course of the reaction (Fig. R5), directly demonstrating that the presence of PE and PP promotes the oxidative conversion of PS at temperatures far below its standalone reaction temperature. This observation is fully consistent with the proposed wax-mediated co-dispersion mechanism and confirms that the mixed polyolefin system benefits from synergistic interactions between components.

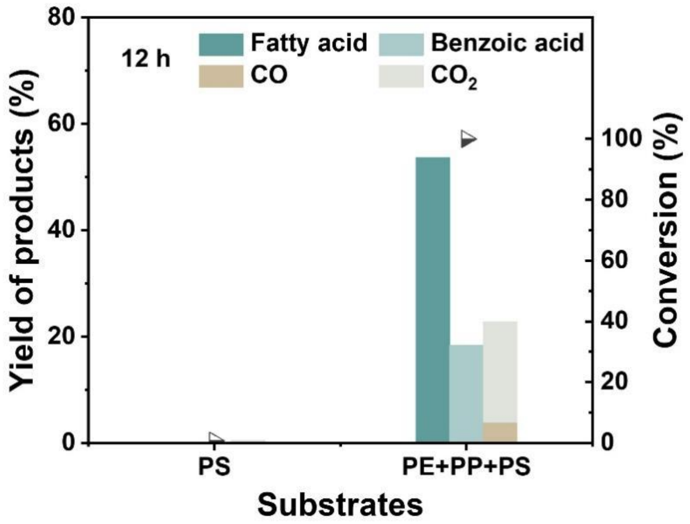

Fig. R5. The product yields from PS alone and mixed polyolefins (PE, PP, PS).

The specific revisions are outlined below:

Page 43 in the Supporting Information:

In mixed polyolefin blends, PE melts first upon heating and undergoes partial oxidative degradation to generate low-molecular-weight wax-like oligomers, which wet and partially swell the surfaces of higher-melting components such as PP and PS, facilitating their co-dispersion into the aqueous phase as polymer-in-water microdroplets under mechanical agitation.

Comment 10. *Rebuttal, Comment 14: In the FTIR spectra, only carbonyl peaks are labeled. O–H and C–O stretching bands should also be clearly indicated. Furthermore, the provided NMR spectra lack peak assignments. Protons associated with hydroxyl, aldehyde, and other functional groups typically resonate beyond 3 ppm, yet this region is not shown. The spectra should be expanded, properly annotated, and included in the Supporting Information to allow rigorous evaluation.*

Response: We thank the reviewer for this comment. In response, we have revised the FTIR spectrum to include comprehensive peak assignments beyond the carbonyl region, now explicitly labeling the broad O-H stretching band at 3196 cm^{-1} (carboxylic acid), C-H stretching at ~2900 cm^{-1}, C=O stretching at 1777 cm^{-1} (anhydride) and 1708 cm^{-1} (carboxylic acid/ketone), $-CH_2-$ bending at ~1462 cm^{-1}, C-O stretching at 1169 cm^{-1}, and O-H out-of-plane bending at 940 cm^{-1}. No band near 1630 cm^{-1} (δ H-O-H) is observed, excluding water as the source of the O-H signals. The ^{1}H NMR spectra have been expanded to cover the full 0-12 ppm range and are now presented as two complementary panels. In the 3-12 ppm panel, peaks are assigned to -CHO (~9.7 ppm, aldehyde proton), olefinic protons arising from β-scission of the PE backbone (~5.1

ppm, internal -CH=CH-; ~4.8 ppm, terminal $=CH_2$), and -CH-OH (~3.6-4.4 ppm, hydroxyl-bearing methine/methylene protons), providing direct spectroscopic evidence for key oxidation intermediates including hydroperoxides and aldehydes. In the 0-3 ppm panel, peaks are assigned to $-CH_2$-COOH (methylene protons in the α-position relative to the carboxylic acid group, ~2.3-2.5 ppm), $-CH_2$-CO- (methylene protons in the α-position relative to a carbonyl group, ~2.1 ppm), $-CH_2$- (methylene protons in the β-position relative to a carbonyl group, ~1.6 ppm), $-CH_2$- (inactivated backbone methylene protons of residual PE, ~1.2 ppm), and $-CH_3$ (terminal methyl protons, ~0.8 ppm). These revised spectra have been incorporated into the Supporting Information. Collectively, the annotated FTIR and NMR data provide rigorous spectroscopic characterization of the oxidation intermediates, fully addressing the reviewer's concern.

Functional group	FTIR (cm^{-1})	^{1}H NMR (ppm)
O-H (carboxylic acid)	3196 (broad), 940	—
C=O (carboxylic acid)	1708	2.3-2.5 (CH_2-COOH)
C=O (anhydride)	1777	(no special proton peak)
C=O (aldehyde)	1735	9.7 (-CHO)
C-O stretch	1169	3.6-4.4 (-CH-OH)
Olefin (-CH=CH-, $=CH_2$)	(overlapped by C-H/O-H)	5.1, 4.8
PE backbone ($-CH_2$-)	1462	1.2, 1.6, 0.8

The specific revisions are outlined below:

Page 33 in the Supporting Information:

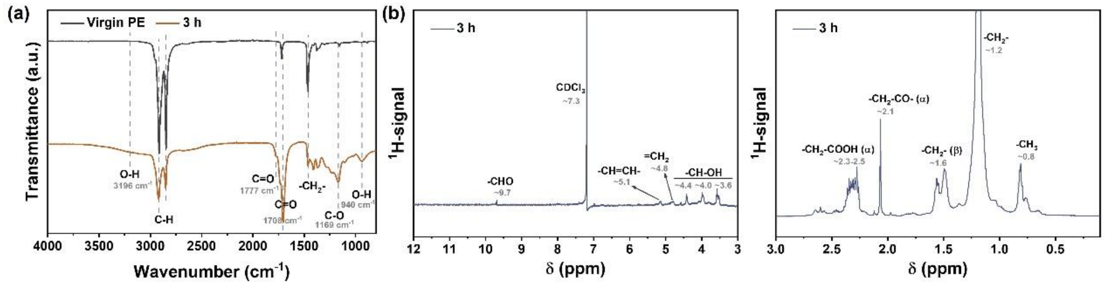

Fig. S23. (a) The IR spectrum of virgin PE and the solid residue after 3 h. (b) The ^{1}H-NMR spectrum of the solid residue after 3 h.

The oxidized polymer shows multiple emerging carbonyl bands, including C=O stretching at 1777 cm^{-1} (anhydride), 1735 cm^{-1} (aldehyde), and 1708 cm^{-1} (carboxylic

acid/ketone), alongside a broad O-H stretching band at 3196 cm^{-1} and O-H out-of-plane bending at 940 cm^{-1} (no band near 1630 cm^{-1}, excluding water), collectively confirming the introduction of oxygenated functionalities along the polymer backbone. The C-O stretching band at 1169 cm^{-1} further indicates the formation of alcohol intermediates, while attenuation of the -CH_2- scissoring band at 1462 cm^{-1} relative to virgin PE is indicative of backbone oxidation and chain scission. [13] These FTIR assignments are fully consistent with the ^1H NMR results, which reveal aldehyde protons at ~9.7 ppm, olefinic protons at ~5.1 (-CH=CH-) and ~4.8 ppm (=CH_2) arising from β-scission of the PE backbone, hydroxyl-bearing methine protons at ~3.6-4.4 ppm, and α-methylene protons adjacent to carboxylic acid groups at ~2.3-2.5 ppm. [14] Notably, the olefinic signals are of very low intensity, indicating that it is a minor pathway.

Comment 11. *Fig. S34: There appears to be a typo "Electic price" should be corrected to "Electric price."*

Response: We thank the reviewer for carefully noting this typographical error. We have corrected "Electic price" to "Electric price" in the revised Fig. S36.

The specific revisions are outlined below:

Page 45 in the Supporting Information:

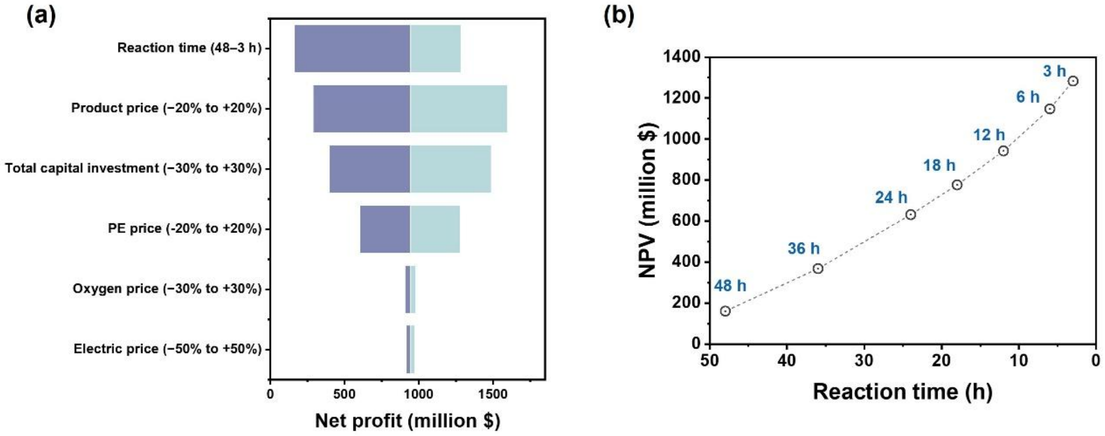

Fig. S36. (a) One-way sensitivity analysis of net present value (NPV). (b) Sensitivity analysis of net present value (NPV) as a function of reaction time.

Comment 12. *Fig. S29b: The DSC thermograms presented correspond to the first heating cycle. For polymers, it is standard practice to report DSC data from the second heating cycle (Heat – Cool – Heat) to erase any prior thermal history and obtain reproducible melting and crystallization behavior.*

Response: We thank the reviewer for this important comment. In response, we have re-measured the DSC thermograms using a Heat-Cool-Heat protocol and now report the

second heating cycle in the revised Fig. S30. The key thermal transition temperatures show no significant deviation from those reported in the original manuscript.

The specific revisions are outlined below:

Page 39 in the Supporting Information:

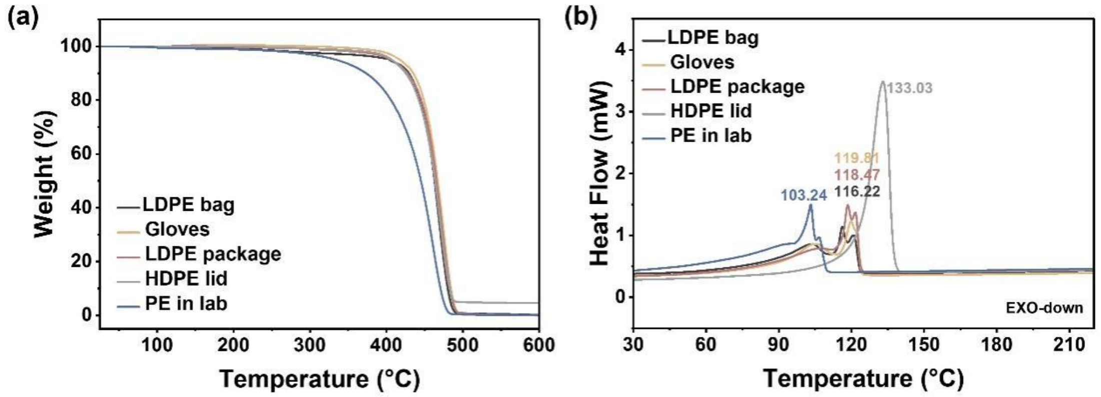

Fig. S30. (a) TG curves and (b) DSC curves of commercial PE and PE standard sample in lab. The test results of the five samples exhibited significant overlap, indicating that the thermal decomposition behaviors of the four commercial PEs are essentially analogous to that of standard PE in lab.

…The laboratory PE standard exhibits a slightly lower apparent melting temperature than the commercial PE samples, which can be explained by differences in molecular weight.

Comment 13. *Abstract, Line 29: The term "fatty acids" should be replaced with "carboxylic acids," as the reported products include a range of monoacids, diacids, and benzoic acid depending on the feedstock.*

Response: We thank the reviewer for this correction. We have replaced "fatty acids" with "carboxylic acids" to more accurately reflect the full range of products obtained, including monoacids, diacids, and benzoic acid depending on the feedstock.

The specific revisions are outlined below:

Page 1 line 26 in the revised manuscript:

Here, we report a catalyst-free upcycling strategy that leverages in situ generation of hydroxyl radicals at microdroplet interfaces [5-8] to enable oxidative cleavage of diverse waste plastics-from polyolefins to rubbers-into carboxylic acids under mild conditions.

Comment 14. *Abstract, Lines 39-42: The reported numerical values should be updated to reflect the revised 60 kton/year basis.*

Response: We thank the reviewer for this comment. We have updated all numerical values in the Abstract to reflect the revised 60 kton/year processing basis in the revised manuscript.

The specific revisions are outlined below:

Page 2 line 9 in the revised manuscript:

Techno-economic analysis projects a post-tax profit of $72.1 million annually and a 3.3-year payback period for a 60 kton/year facility, highlighting its industrial potential.

Point-by-point response to reviewers' comments

Title: "Catalyst-Free Valorization of Waste Plastics via Microdroplet-Interfacial Radical Scission"

Corresponding Author: Prof. Graham J. Hutchings

The reviewer's comments are shown in black; our response is shown in blue.

All revisions in the MS are highlighted in yellow.

Reviewers #2 & 3

Comments to the Author:

We thank the authors for carefully considering our previous comments and for revising the manuscript accordingly. After reviewing the revised manuscript and the response-to-reviewers document, we believe that most of our concerns have been satisfactorily addressed, with the exception of a few points. Once these are adequately resolved, we recommend the manuscript for publication.

Response: We sincerely thank the Reviewer for the thorough re-evaluation of our revised manuscript and for the constructive and encouraging assessment. We are pleased that the majority of concerns raised in the previous round have been satisfactorily addressed. In the following, we provide point-by-point responses to the remaining comments and have revised the manuscript accordingly. We hope that the revised version fully meets the Reviewer's expectations and that the manuscript is now suitable for publication in Nature.

Comment 1. *Rebuttal, Comment 3: The authors attempt to explain the reactivity of PP based on its Vicat softening point, citing a range of ~80–120 °C, which is below the reaction temperature of 125 °C. However, values reported in the literature for PP are typically higher (~130–170 °C). This discrepancy raises the question of whether the reported value is specific to the PP used in this study. Given that the molecular weight of the PP employed here appears to be very low (~5 kDa), this may be possible. It would be useful to clarify this point. Additionally, it would be useful to comment on whether the same rationale would apply to isotactic PP of higher molecular weight, and whether the reaction would still proceed under the given conditions? The explanation provided*

for PS is satisfactory.

Response:We sincerely thank the Reviewer for this exceptionally perceptive and constructive comment. The Reviewer makes an excellent point. The initial range we cited from general literature did not adequately account for the specific characteristics of our material, and we greatly appreciate the Reviewer highlighting the critical role of molecular weight (Mw).

Following the Reviewer's insightful guidance, we have taken two decisive steps to clarify these points:

1. Direct Measurement of the Low-Mw PP: We have now directly measured the Vicat softening point of the specific low-Mw PP (~kDa) used in our study, and the results have been added to Extended Data Fig. 8m. As the Reviewer insightfully anticipated, the primary Vicat softening point of this sample is ~150 °C. However, the measurement captured a distinct initial probe response at ~120 °C, corresponding to the onset of partial melting—a critical stage where the probe no longer slips freely, signaling the incipient softening of the polymer matrix. This observation is fully consistent with the DSC onset temperature (~120 °C) for this specific low-Mw sample. To clarify this for the readers, we have explicitly noted in Extended Data Fig. 8m that the Vicat softening point varies with the molecular weight of PP, and the reaction temperature should ideally be aligned near this onset/softening threshold to ensure sufficient polymer mobility and interfacial contact.

2. Validation with High-Mw Isotactic PP: To address the Reviewer's excellent question regarding whether this rationale applies to higher-molecular-weight feedstocks, we performed additional experiments using a commercial isotactic PP with a significantly higher molecular weight (Mw ~250,000 Da) under identical conditions (Extended Data Fig. 8n). At the original temperature of 125 °C, the conversion was noticeably reduced compared to the low-Mw PP, as the higher-Mw polymer remained rigid. Remarkably, when we modestly increased the reaction temperature to 140 °C, full conversion was successfully restored, delivering comparable product yields.

These new results beautifully validate the Reviewer's hypothesis. They confirm that the same mechanistic rationale applies universally to higher-Mw PP feedstocks, albeit requiring a correspondingly higher operating temperature to achieve the necessary thermal softening and sufficient contact at the gas-water interface.The manuscript and supplementary figures have been updated accordingly to reflect these insights. We believe these additions have significantly strengthened the mechanistic rigor of our work, and we are deeply grateful to the Reviewer for prompting this improvement.

The specific revisions are outlined below:

Page 8 in Extended Data:

l-m, PP with different molecular weights and tacticity needs different operating temperatures for complete conversion, owing to their differing thermal softening behavior. For higher-molecular-weight isotactic PP, an elevated reaction temperature is required to achieve high conversion.

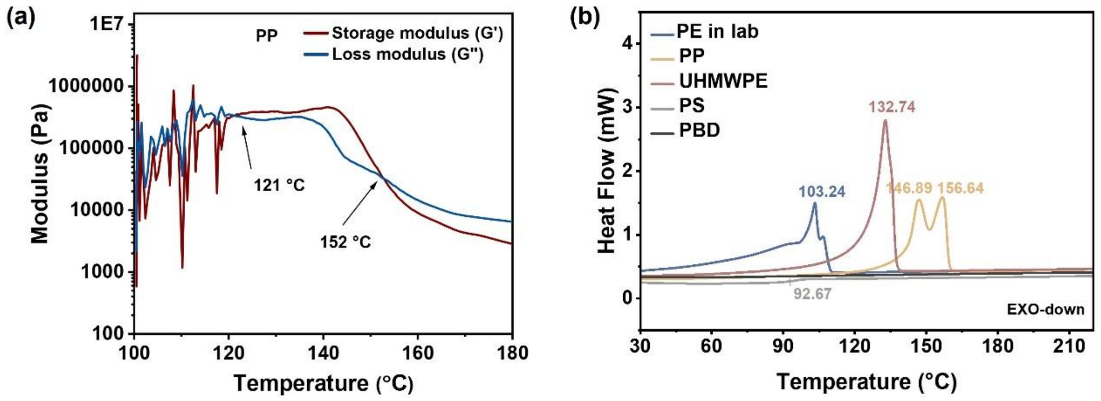

Extended Data Fig. 8l. (a) Dynamic mechanical analysis (DMA) of PP. (b) DSC melting curves of all polyolefin substrates employed.

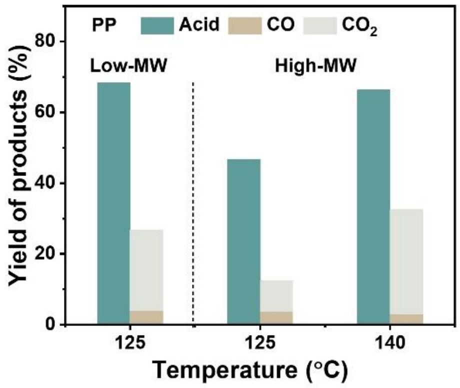

Extended Data Fig. 8m. The yield of products of low-MW PP and high-MW isotactic PP oxidation under different temperatures after 12 h.

Comment 2. *Rebuttal, Comment 5.1: I thank the authors for the clarification regarding the market-mix technology and for providing additional comparison with mechanical recycling. While I understand the authors' position that direct comparisons between chemical upcycling and mechanical recycling must be made with care, given that mechanical recycling preserves polymer chain integrity and therefore typically involves lower energy input and carbon emissions, I note that the other benchmarks used (landfilling, incineration, and market-mix scenarios) are likewise not directly comparable. A more rigorous comparison would ideally involve other chemical*

recycling or upcycling technologies that generate value from polyethylene (e.g., pyrolysis, gasification, catalytic cracking, hydrocracking). That said, I acknowledge that such an analyses may be beyond the scope of the present study. My original recommendation to include mechanical recycling was motivated by its status as one of the most mature and widely implemented waste management strategies. Its inclusion would provide a valuable baseline, enabling readers to better contextualize the performance of the proposed chemical approach. At the same time, elucidating the inherent limitations of mechanical recycling would further strengthen this context. Although mechanical recycling may exhibit lower energy and carbon intensity, this does not detract from the potential significance of the proposed technology. Rather, incorporating it as a benchmark would enhance the transparency and contextual robustness of the analysis. I therefore maintain my recommendation that mechanical recycling be included in the comparison.

Response: We are deeply grateful to the Reviewer for this exceptionally thoughtful, balanced, and constructive guidance. We sincerely appreciate the Reviewer's elegance in articulating both the contextual value of mechanical recycling (MR) as a baseline and the inherent complexities of cross-technology comparisons.

The Reviewer is entirely correct that including MR provides a crucial, mature benchmark that enhances the transparency and robustness of our environmental assessment.

We completely agree with the Reviewer regarding the methodological challenges this introduces. In life cycle assessment (LCA) practice, integrating MR as a baseline indeed presents unique complexities because MR yields intermediate recycled pellets rather than final functional chemicals. To construct a rigorous and meaningful comparison, one must carefully account for:

1. Downstream application pathways for the recycled pellets;

2. Quality degradation factors (downcycling) relative to virgin materials;

3. Regulatory constraints that restrict recycled PE from food-contact or medical applications.

Spurred by the Reviewer's compelling recommendation, we embraced this challenge and have now successfully integrated MR into our comparative LCA framework (Extended Data Fig. 10 and Supplementary Text 2), employing highly transparent and conservative assumptions to ensure a fair comparison:

1. Feedstock Composition: We assumed the waste PE stream reflects the European market mix (58% LDPE and 42% HDPE) as a realistic proxy.

2. Quality Discount Factors: To rigorously account for property degradation and market/regulatory limitations, we applied standard substitution factors of 0.50 for LDPE and 0.65 for HDPE, in strict accordance with established plastic waste management literature.s

The updated results yield highly encouraging insights: even under these conservative credit assumptions, our proposed chemical upcycling technology achieves a climate mitigation performance highly comparable to that of traditional MR (-0.30 vs. -0.35 kg CO_2-eq, as shown in **Extended Data Fig. 10**).

As the Reviewer perceptively noted, this comparison does not detract from our technology; rather, it beautifully highlights the complementary value of our approach. While MR excels in low-energy loop retention for certain sectors, our upcycling process offers a vital alternative for waste fractions that MR cannot upgrade, transforming them into high-value chemical products.

We thank the Reviewer once again for maintaining this recommendation. This additional analysis has immensely elevated the transparency, depth, and contextual robustness of our manuscript.

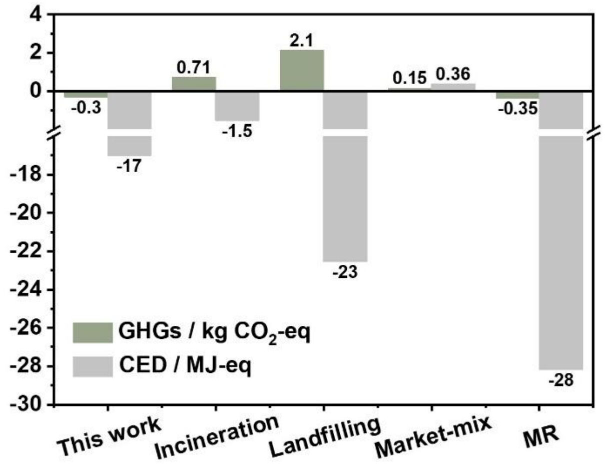

Extended data Fig. 10b. Greenhouse gas emissions (GHGs) and cumulative energy demand (CED) for the treatment of 1 kg of plastic via: the proposed upcycling technology, incineration, landfilling, the conventional market-mix technology, and mechanical recycling (MR).

Comment 3. *Rebuttal, Comment 9: The authors propose that "the role of PE is not to act as a thermodynamic solvent for PP or PS, but rather to generate low-molecular-weight, wax-like oligomers upon partial oxidative degradation, which mediate the dispersion of higher-melting polymers into the aqueous phase through wetting and swelling interactions." To support this hypothesis, the authors present model*

experiments using C17 alkane as a proxy for the low-molecular-weight wax-like fraction and demonstrate that C17 can successfully disperse PP and PS. While this result is not unexpected, given that small alkanes are known to be effective solvents for polymers, it raises a question regarding the nature of the intermediates formed under the proposed reaction conditions. Based on the proposed mechanism, one might instead expect more polar species (e.g., alcohols, acids, or aldehydes/ketones). It is therefore unclear whether the same dispersive behavior would persist for such intermediates given their higher polarity. That said, the evidence provided in Fig. R5 demonstrating that the reaction proceeds at lower temperatures in the presence of PE is compelling. Additional experiments may not be strictly necessary; however, the authors should clarify this point or appropriately qualify their mechanistic interpretation.

Response: We are deeply grateful to the Reviewer for this exceptionally sharp and profound insight regarding the polar nature of the PE oxidation intermediates. The Reviewer raises a fundamental question about whether oxygenated species (such as acids and aldehydes) retain the same dispersive capacity as nonpolar alkanes.

Although the Reviewer kindly noted that additional experiments might not be strictly necessary, we felt that this brilliant point deserved a direct, empirical validation to ensure the absolute rigor of our mechanistic framework.

Therefore, we eagerly performed heating-dissolution tracking experiments using two representative polar model compounds that mimic the actual oxidative scission intermediates generated in our system: octadecanedioic acid (a long-chain dicarboxylic acid) and nonanal (an aliphatic aldehyde).

The new experimental results, now integrated into Extended Data Fig. 8n, provide compelling confirmation:

1. Both PP and PS exhibit substantial swelling and partial dissolution in each of these oxygenated media at 140 °C.
2. This directly demonstrates that the essential wetting, swelling, and dispersive capabilities are safely maintained despite the higher polarity introduced by the oxygenated functional groups.

These findings beautifully enrich our mechanistic interpretation. They confirm that as the PE chains undergo progressive oxidative scission, the emerging pool of fragments—regardless of whether they are nonpolar oligomeric waxes or polar oxygenated intermediates—collectively and seamlessly function as an effective dispersing medium. This medium systematically reduces the mass-transfer barriers for the higher-melting polymer matrices (PP and PS), guiding them into the active aqueous interface.

We have refined the text in the revised manuscript to appropriately qualify and detail this nuance. We sincerely thank the Reviewer for steering us toward this critical clarification, which has vastly improved the depth and credibility of our proposed mechanism.

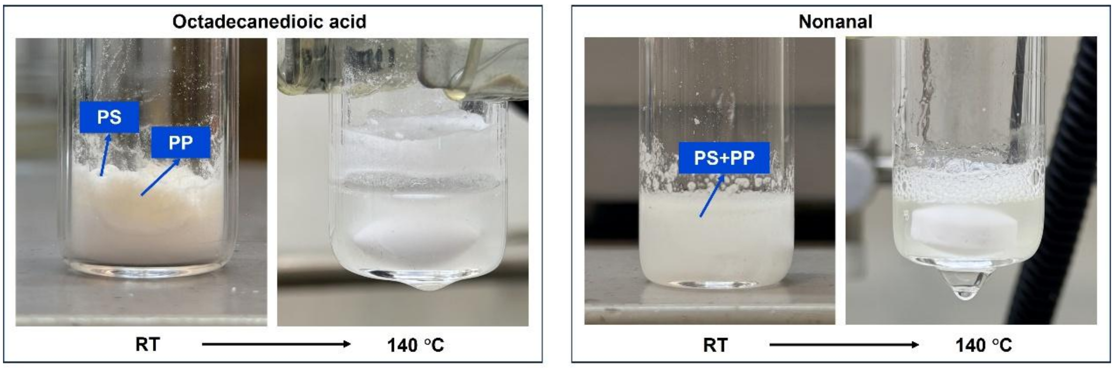

Extended data Fig. 8n. Photographs showing the interaction of PS and PP with polar oxidation-product analogues at room temperature (RT) and 140 °C. Left panel: PS and PP in octadecanedioic acid; right panel: PS and PP mixture in nonanal.
